# Practical Guide and Best Practices for Diffusion NMR Processing With GNAT

**DOI:** 10.1002/mrc.70110

**Published:** 2026-05-10

**Authors:** Tadeu Luiz Gomes Cabral, Guilherme Dal Poggetto, Claudio F. Tormena, Mathias Nilsson

**Affiliations:** ^1^ Chemistry Institute University of Campinas ‐ UNICAMP Campinas São Paulo Brazil; ^2^ Analytical Research & Development Merck & Co. Inc. Rahway New Jersey USA; ^3^ Department of Chemistry University of Manchester Manchester UK

## Abstract

Diffusion‐NMR, including diffusion‐ordered spectroscopy (DOSY), is a powerful technique for measuring self‐diffusion and inferring molecular size, shape, aggregation, and intermolecular interactions in solution. Its noninvasive nature and ability to separate components in complex mixtures have made DOSY widely used across chemistry, biochemistry, and materials science. However, accurate and reproducible diffusion measurements depend not only on careful experimental design but also on robust, transparent, and consistent data processing. Despite the growing adoption of diffusion‐NMR, widely adopted protocols, practical tutorials, and broadly accepted guidelines for DOSY data analysis remain limited, contributing to variability in reported diffusion coefficients and reduced comparability between studies. The General NMR Analysis Toolbox (GNAT) is a free, open‐source platform for visualizing and analyzing diffusion‐NMR data. In this article, we present a clear, step‐by‐step guide to processing diffusion‐NMR data with GNAT, emphasizing best practices, quality control, and common pitfalls. The workflow covers data import, preprocessing (including baseline/phase considerations and signal selection), diffusion fitting and model choice, validation of results, and recommendations for reporting key parameters and uncertainty. Although written with new users in mind, this guide also serves as a reference for experienced practitioners seeking greater consistency and rigor in DOSY analysis. By promoting standardized, reproducible workflows and encouraging the use of open‐source tools, this work aims to improve the transparency, quality, and comparability of diffusion‐NMR studies.

## Graphical abstract

A practical and reproducible workflow for diffusion‐NMR (DOSY) data analysis is introduced through a step‐by‐step GNAT tutorial. By establishing clear processing guidelines, validation strategies, and reporting standards, this work aims to reduce variability in diffusion measurements and promote more transparent, comparable, and reliable DOSY studies across the NMR community.

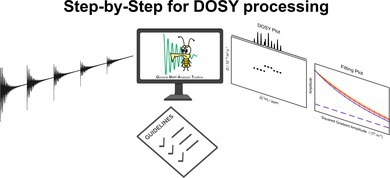

## 1 Introduction

**FIGURE 1:**
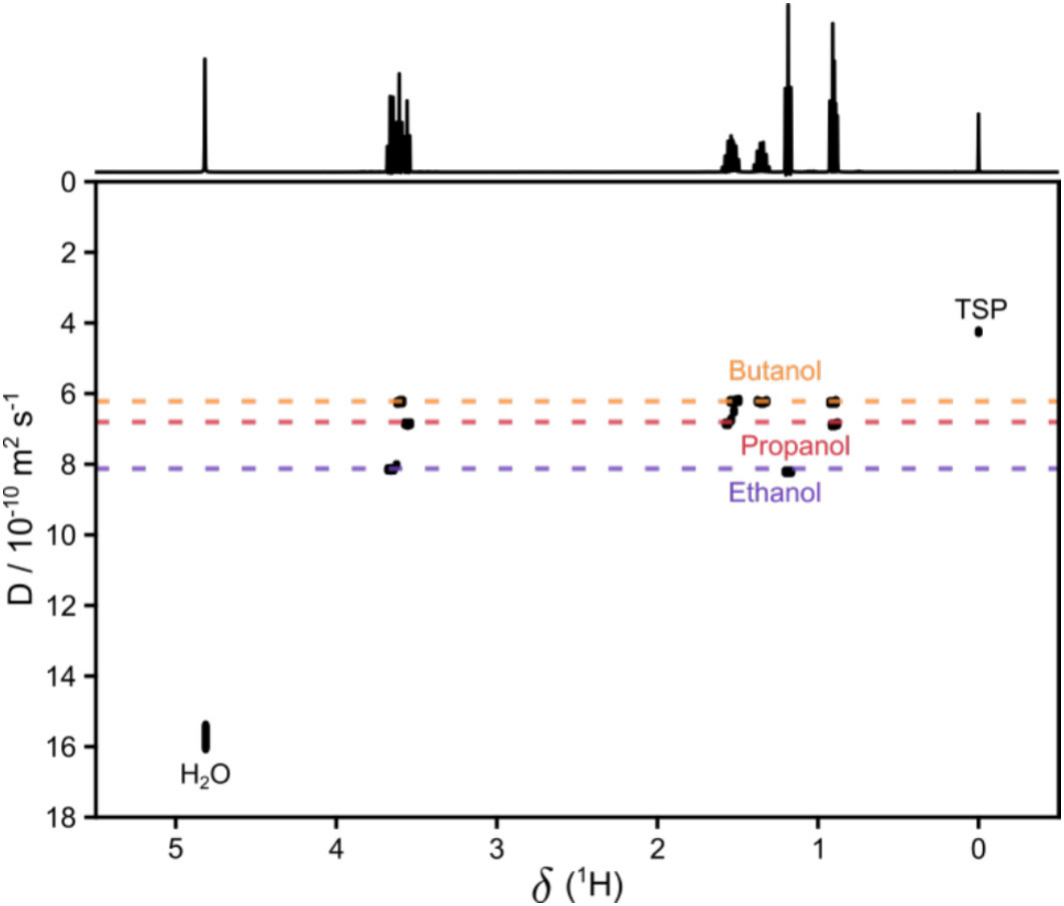
^1^H‐DOSY plot featuring the least attenuated 1D spectrum at the top, for a mixture of ethanol, butanol, and 1‐propanol in D_2_O and TSP as a reference. The spectra of the mixture components are separated in the diffusion dimension.

Diffusion‐NMR is widely used to study molecular size, shape, aggregation, and intermolecular interactions in solution [1, 2, 3, 4, 5]. When diffusion‐NMR is applied to mixture analysis, it is often referred to as diffusion‐ordered spectroscopy (DOSY), in which diffusion encoding allows the separation and identification of chemical species in a mixture based on their translational diffusion coefficients [6, 7]. Strictly speaking, DOSY is one way of visualizing such diffusion‐NMR data, but the name is often used synonymously with diffusion‐NMR—we will use DOSY as interchangeable with diffusion‐NMR in this work. There are many other ways of processing diffusion NMR data [8, 9, 10], but DOSY remains the main workhorse for mixture analysis and is the focus of this publication. It can often be helpful to combine DOSY processing with other methods, such as SCORE [11, 12, 13], DECRA [14, 15, 16, 17], and ILT [10, 18]. Unlike traditional NMR methods that provide, for example, chemical shift or coupling information, DOSY, when used for mixture analysis, can recover individual component spectra (virtual separation) by correlating diffusion behavior with molecular size, shape, and interactions with the surrounding environment [5, 19, 20, 21]. This approach is illustrated in Figure 1 and in the literature [4, 5, 22, 23]. Diffusion‐NMR can also be used for analyzing supramolecular assemblies [24, 25, 26, 27, 28], polymers [2, 29, 30, 31], and for molecular mass determination [32, 33, 34, 35, 36].

A DOSY experiment utilizes pulsed field gradients (PFGs) to encode diffusion‐related signal attenuation, allowing for the generation of a pseudo two‐dimensional spectrum (Figure 1) where one axis represents the conventional chemical shift and the other corresponds to the diffusion coefficient, where the peak is centered on the fitted diffusion coefficient and the width of the peak is determined by the statistics of the fit [6, 37, 38, 39]. In practice, a 2D DOSY plot is obtained by processing a series of one‐dimensional NMR spectra acquired with progressively increasing gradient strengths. The attenuation of the signal is described by the Stejskal–Tanner equation [40]:

(1) $$S_{g}=S_{0}e^{-D\gamma^{2}\delta^{2}g^{2}\Delta^{\prime }}$$

where $S_{g}$ is the signal amplitude, *S*_0_ is the signal intensity in the absence of diffusion, *D* is the diffusion coefficient, *γ* is the gyromagnetic ratio of the nuclear spins, *δ* is the gradient pulse width, g is the gradient amplitude, and $\Delta^{\prime }$ is the diffusion encoding time corrected for the effects of finite gradient pulse width, to obtain the diffusion coefficient values [41].

To obtain reliable diffusion coefficients, it is essential to follow a systematic protocol that considers several critical acquisition and processing parameters involved in the DOSY experiment. Typically, the acquisition of high‐quality diffusion data begins with proper thermal equilibration of the sample inside the magnet. After inserting a new sample, it is advisable to allow several minutes (typically 10–20 min) for temperature stabilization before initiating any acquisition protocols. Subsequently, routine spectrometer adjustments (such as shimming, lock correction, and tuning) should be carefully performed to ensure magnetic field homogeneity and signal stability. An optimized lock frequency is particularly important as the PFGs dephase the signal and “kill” the lock.

It is important to use a spectral width that is sufficiently large to accommodate the entire signal region with ample baseline margin. A clean baseline, extending roughly (at least) one‐third of the spectrum on each side, facilitates effective baseline correction during processing. The gradient increment selection is another important parameter: a typical DOSY experiment employs between 10 and 30 gradient levels, but a higher number is recommended when targeting a wide range of diffusion coefficients or when employing advanced processing techniques. The gradient increment scheme (e.g., linear, quadratic, or exponential steps between the gradients) should be selected based on the diffusion range and sample characteristics. For most applications, a quadratic step (equal steps in the squared gradient amplitude) provides a good starting point.

An additional critical consideration is the signal attenuation across the gradient increments. For standard DOSY experiments (where a monoexponential fit is used), the signal should decay by approximately 80% between the first and last gradient increments to achieve optimal fitting accuracy. For more complex analyses, such as multiexponential fitting or multivariate approaches, a higher attenuation (up to 95%) can be advantageous [42]. It is also recommended to begin with a minimum gradient strength of 10% of the maximum value (higher than the often‐set default of 2%). Too low gradient strengths often yield poor results due to inefficient coherence selection and nonlinearity in the gradient. The optimal upper limit depends on the pulse sequence employed—for the Oneshot [43] sequence used in this work, gradients above 80% may influence coherence selection and should therefore be avoided; the sequence is based on the bipolar‐pulse pair stimulated echo (BPPSTE) [6, 7] sequence, adding extra gradients to improve coherence transfer pathway (CTP) selection in such a way that using too high a gradient strength negates the improvement in CTP selection. There are several pulse sequences available for diffusion NMR, but for many purposes, BPPSTE or Oneshot is a good choice [7].

The number of scans per gradient level also makes a difference to the quality of the resulting diffusion coefficients—not just for the signal‐to‐noise ratio (SNR) but also as insufficient phase cycling gives incomplete diffusion decay (some of the magnetisation have not experienced the full PFG effect). As a rule of thumb for Oneshot experiments: a single scan can suffice for a quick screening (when diffusion encoding and coherence transfer pathway selection are implemented along orthogonal gradient axes a more accurate results can be achieved with a lower number of scans) [44, 45, 46], while four scans generally yield reliable data, and 16 scans provide a clean and good quality result (further improvement is still possible with 32, 64, 128, and 256, if lengthy experimental times can be tolerated). For the conventional BPPSTE pulse sequence, approximately four times more scans are typically required to achieve comparable results.

Diffusion NMR experiments are also susceptible to other experimental artifacts, including eddy currents [47], convection [48, 49], imperfect coherence problems [50], and *J*‐modulation [51]. These effects can be mitigated by using appropriate pulse sequences or experimental adaptations. For instance, convection effects can often be reduced by using NMR tubes with a diameter of 3 mm or less, by choosing solvents less susceptible to convection when practical, or, at a cost in sensitivity and increased phase cycling (for sequences based on the stimulated echo), by using convection‐compensated pulse sequences, such as double‐stimulated echo sequences or double diffusion encoding scheme [48]. As this work focuses primarily on data processing, only key aspects of the acquisition protocol are summarized here; more detailed acquisition strategies can be found in the literature [9, 17, 38, 52, 53].

A further source of systematic error in diffusion‐NMR experiments arises from the spatial nonuniformity of the PFGs. Although modern actively shielded gradient coils offer excellent linearity and stability, compromises between gradient strength, switching speed, and gradient noise levels often lead to residual nonuniformity across the active sample volume. As a result, different regions of the sample experience slightly different effective gradient amplitudes, causing deviations from the ideal exponential signal decay described by the Stejskal–Tanner equation (Equation 1). This effect becomes particularly significant at high gradient strengths or large signal attenuations, leading to systematic underestimation or overestimation of diffusion coefficients [54, 55, 56]. In practice, this varies widely between probes, and some standard probes have sufficient uniformity that it has only a minor effect in most applications. It is, however, prudent to check the state of your probe—the manufacturer should be able to provide that information.

Several approaches have been proposed to mitigate the effects of poor gradient uniformity. Simple strategies include using shorter sample heights or slice‐selection techniques to restrict the measurement to a smaller, more uniform region of the gradient field; however, both approaches severely compromise the SNR and spectral resolution [57, 58, 59, 60, 61, 62]. A more rigorous approach involves explicitly correcting for gradient nonuniformity by modifying the fitting model to account for the spatial gradient distribution within the sample. This correction enables accurate determination of the diffusion coefficient without sacrificing sensitivity. Incorporating this correction into the fitting procedure is especially important in multiexponential and multivariate DOSY analyses, where deviations from ideal exponential decay can otherwise be misinterpreted as artificial components or distortions in the diffusion dimension [9, 63]. Hence, accurate calibration and proper treatment of gradient nonuniformity are essential for obtaining reliable and reproducible diffusion coefficients, particularly when quantitative comparison between samples or reference materials is required.

Regarding data processing, the main focus of this work, several commercial and free software packages are available for processing DOSY data, such as the DOSY Toolbox [64], Bruker TopSpin (Dynamics Center), Mestrelab (Mnova), and JEOL Delta (JASON), among others commonly used in the field. The workflow will be similar in several of these software packages. In this tutorial, we have selected the GNAT software [65], which is based on the MATLAB environment. GNAT is open‐source, and for users without access to MATLAB, there is a free‐standalone, compiled version with full functionality.

This tutorial presents a recommended workflow for DOSY data processing with GNAT, providing a clear, step‐by‐step guide aimed primarily at new users. It demonstrates how general DOSY processing principles can be effectively implemented within the GNAT environment and highlights how parameters influence the quality and reliability of the resulting diffusion data. Comprehensive discussions of the theoretical background, complementary information, acquisition strategies, and the internal structure of GNAT are beyond the scope of this work and can be found in the cited literature and GNAT manual [66].

## 2 Experimental Procedures

In this publication, we use a relatively simple sample—a mixture of ethanol, 1‐propanol, and 1‐butanol in D_2_O with TSP as a reference. The sample is chosen so that the results are easily understood while still highlighting essential challenges. The sample was prepared by dissolving 16.6 mg of ethanol, 14.5 mg of 1‐propanol, and 20.8 mg of 1‐butanol in 750 μL of deuterated water (D_2_O), containing 0.05% v/v trimethylsilylpropanoic acid (TSP) as an internal reference. An additional 2 mg of TSP‐d4 was added to the solution to facilitate reference deconvolution [67, 68, 69].

The NMR experiments were conducted at 298 K in standard 5 mm NMR tubes using a Bruker Avance III spectrometer operating at 400.18 MHz for ^1^H, equipped with a BBI probe. This probe has a relatively uniform gradient, so no correction is needed for the applications in this report. The nominal calibrated *z*‐gradient strength for the probe is 54.5 G cm^−1^. The diffusion measurements were performed using the Oneshot pulse sequence [43] with 16 gradient increments, in which the gradient strength increased quadratically from 10% to 80% of the maximum calibrated value. For each increment, 64 scans were acquired, preceded by 16 dummy scans. Data were collected with 32 k points for the ^1^H frequency dimension. The diffusion encoding period (*Δ* = 0.08 s) and the gradient pulse duration (*δ* = 1100.0 μs) were selected to give a decay of about 75%. The total experiment time was approximately 1 h and 33 min.

The diffusion data were analyzed using GNAT v1.2.3 and v2.1 software (running in MATLAB R2019a). Fourier transformations (FTs) were applied using different window functions, followed by manual and individual phase and baseline corrections. The diffusion coefficients and their associated uncertainties were determined by fitting a monoexponential decay function derived from the modified Stejskal–Tanner equation (Equation 1), applied to selected peaks identified via peak picking.

## 3 Step‐by‐Step Outline for DOSY Processing

This section presents a simple outline for DOSY processing in GNAT. More detail on important topics is provided in the subsequent section, Key Concepts, Best Practices, and Common Pitfalls, below (referred to as Key Concepts), as well as in the Supporting Information (Sections 1–3). The basic functionality, such as zooming and plot control, of GNAT is described in more detail in the GNAT manual [66].

### 3.1 Acquisition

Before processing diffusion data, it is essential to address specific aspects of the acquisition protocol. These preliminary considerations, such as gradient calibration, pulse sequence parameters, and sample stability, directly impact the reliability, accuracy, and reproducibility of the diffusion measurements. Here, we present an outline that will work for most simple cases; other sources discuss this in more detail [9, 70, 71, 72, 73].

In general, several parameters must be optimized in DOSY acquisition to obtain reliable and comparable results, including the pulse sequence, solvent (and viscosity), gradient strength range, diffusion delay (*Δ*), gradient pulse duration (*δ*), and the number and distribution of gradient increments. Further details on the acquisition can be found in the cited references [9, 70, 71, 72, 73]. Below, we summarize the key points relevant to this tutorial.

Precise temperature control is crucial to avoid chemical shift variations and convection effects during the acquisition of the 1D NMR spectra series. Equally important is accurate calibration of the gradient coil strength, as precise gradient amplitude values are required to ensure correct estimation of diffusion coefficient values.

Optimizing the gradient pulse duration (*δ*—p30 in the sequence used) and diffusion time (*Δ*—d20) to achieve approximately 75% signal attenuation between the first and last gradient amplitudes is a good choice for accurate fitting during data processing. Typically for small molecules in dilute solution, the *δ* ranges from 800 to 3000 μs, and *Δ* ranges from 20 to 300 ms, depending on factors such as molecular size and solvent viscosity, which affects the expected diffusion coefficients. Naturally, smaller molecules generally require lower diffusion encoding and larger, more slowly diffusing species require stronger encoding. There is also a compromise to be struck between *δ* and *Δ*, where the diffusion encoding is quadratic for the former and linear for the latter (Equation 1). During *δ*, *T*_2_ relaxation and *J*‐modulation are active, while for *Δ*, *T*_1_ relaxation is active, and therefore a longer *Δ* is often the choice when *T*_1_ ≫ *T*_2_, which should be optimized to achieve the desired signal attenuation. If the attenuation is too small, the decay curve becomes poorly defined, while excessive attenuation increases the noise level in the latter increments. Thus, the approximately 75% criterion provides a sensible balance, ensuring accurate determination of diffusion coefficients in DOSY experiments.

Additionally, proper lock phase correction is important to prevent instability in the lock signal throughout the acquisition.

### 3.2 Import the Data

Once diffusion‐NMR data have been acquired, they can be imported into GNAT for processing. GNAT supports raw data from various sources, including Bruker, Varian, and JEOL. In this tutorial, we illustrate the procedure using data acquired on a Bruker spectrometer.

**FIGURE 2:**
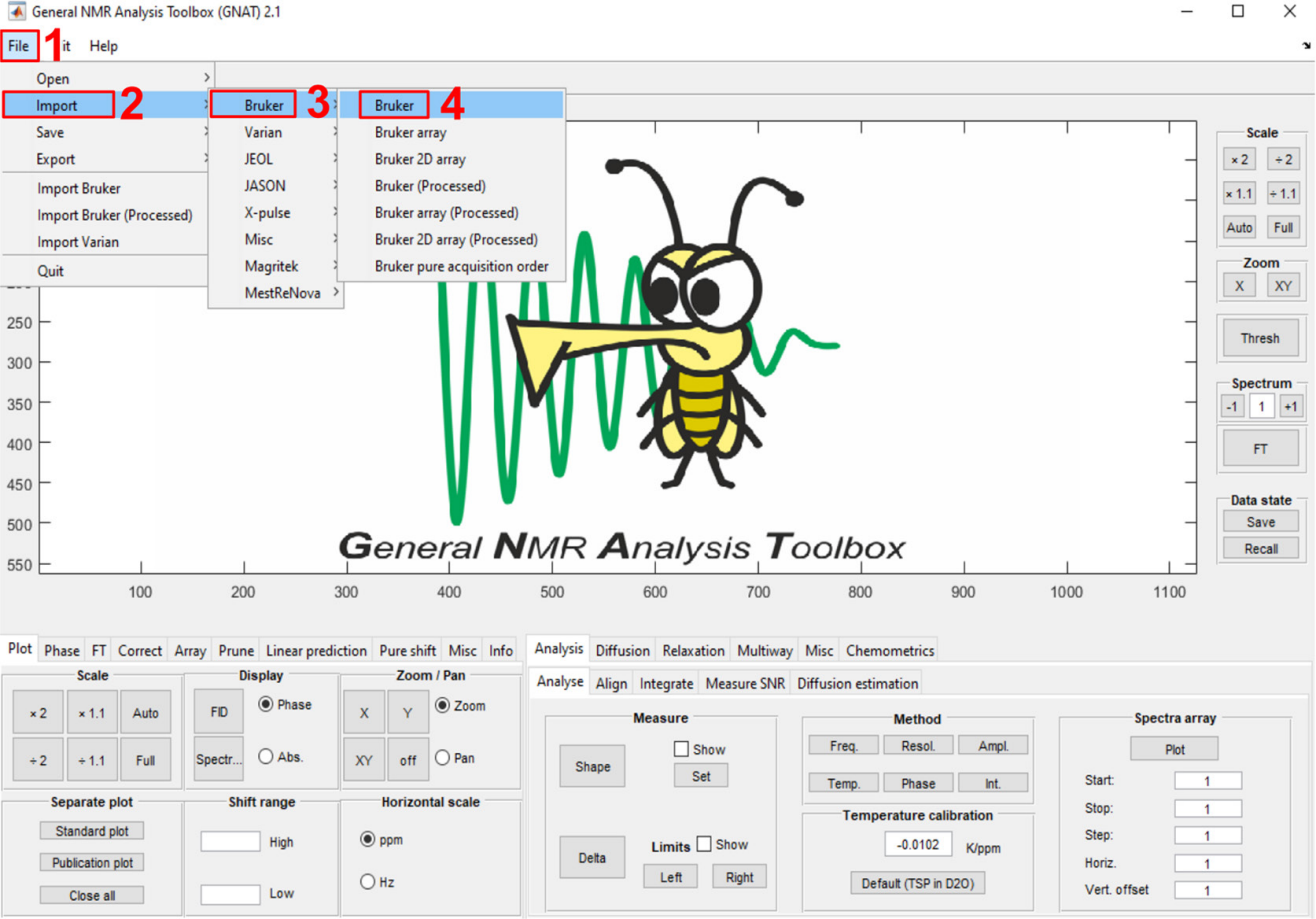
Importing NMR data into GNAT.

To import the diffusion dataset into GNAT, follow the steps outlined below and depicted in Figure 2. Processed Bruker data can also be imported, in which case Steps 1–4 may not be necessary. For additional details on GNAT, including the available controls, buttons, and processing functionalities, refer to the manual available at: https://nmr.chemistry.manchester.ac.uk/. Note that the figures illustrating the step‐by‐step DOSY processing are provided at a larger size in the Supporting Information (Section 3).

- Navigate to: File → Import → Bruker → Bruker
- Select the folder containing the diffusion experiment.
- Click Select Folder to load the dataset.

### 3.3 FT

After importing the FIDs from the diffusion experiment, you can use the *Plot* tab to scale, visualize, and inspect both the FID and the resulting NMR spectrum.

**FIGURE 3:**
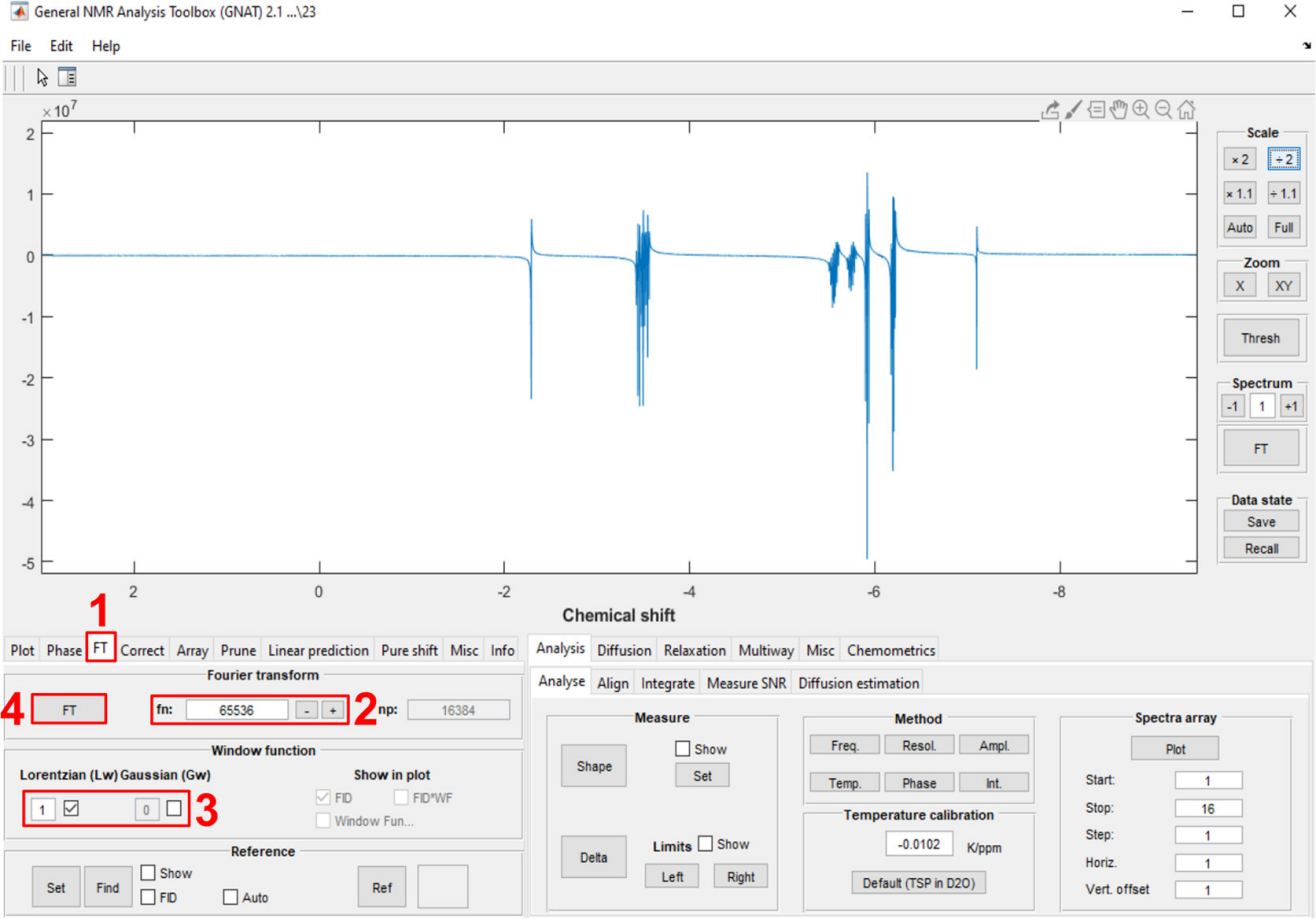
Fourier transformation of a FID into an NMR spectrum using 64k data points and a 1‐ Hz Lorentzian window function.

To transform the FID into a spectrum using the Fourier transform, navigate to the FT panel (see Figure 3). Here, you can specify the desired processing parameters, such as the number of complex points, apply Lorentzian shape/width (Lw) and Gaussian shape/width (Gw) functions, and set the line width in Hz. Follow the steps below to perform the FT:

- Open the *FT* panel.
- Set *fn* (number of complex points [Fourier number]) used in the transformation as desired
- Enable the *Lw/Gw* box and choose the desired values for the line‐broadening/window function.
- Click the *FT* button to apply the FT.

Ideally, to get the most out of DOSY data, the peaks should be resolved in the spectral domain, or the results can be more difficult to interpret [74]. In all but the simplest cases, there is likely to be some overlap, requiring careful interpretation. To minimize overlap, it can be useful to apply resolution enhancement, such as the Lorentz to Gauss transformation. Ideally, this should be done with Reference deconvolution (see below), but using standard window functions also works well. More details on resolution enhancement and reference deconvolution are given in the Key Concepts section below. To avoid spectral overlap, a family of more advanced DOSY methods is available [11, 75, 76, 77, 78] but this is beyond the scope of this publication.

### 3.4 Phase Correction

Properly phased spectra are important to ensure correct DOSY results. A phase error can translate to confusing DOSY spectra. A common situation is that one gets frequency‐dependent diffusion coefficients within a multiplet—a “slanting” multiplet. The problems with phase errors are discussed in more detail in the Key Concepts section. It is not just the general phase correction that matters. In diffusion data, it is not uncommon to observe a gradient‐dependent phase error, which is detrimental to analysis. In GNAT, this can be corrected by adjusting the phases for the spectra of individual gradient levels. In practice, this is often done in two steps: (i) phase correction applied globally to all increments to get a good starting point and (ii) phase correction individually for each increment. Both steps can be performed using either manual or automatic phase correction, with manual correction offering greater control for fine adjustment of the phase.

**FIGURE 4:**
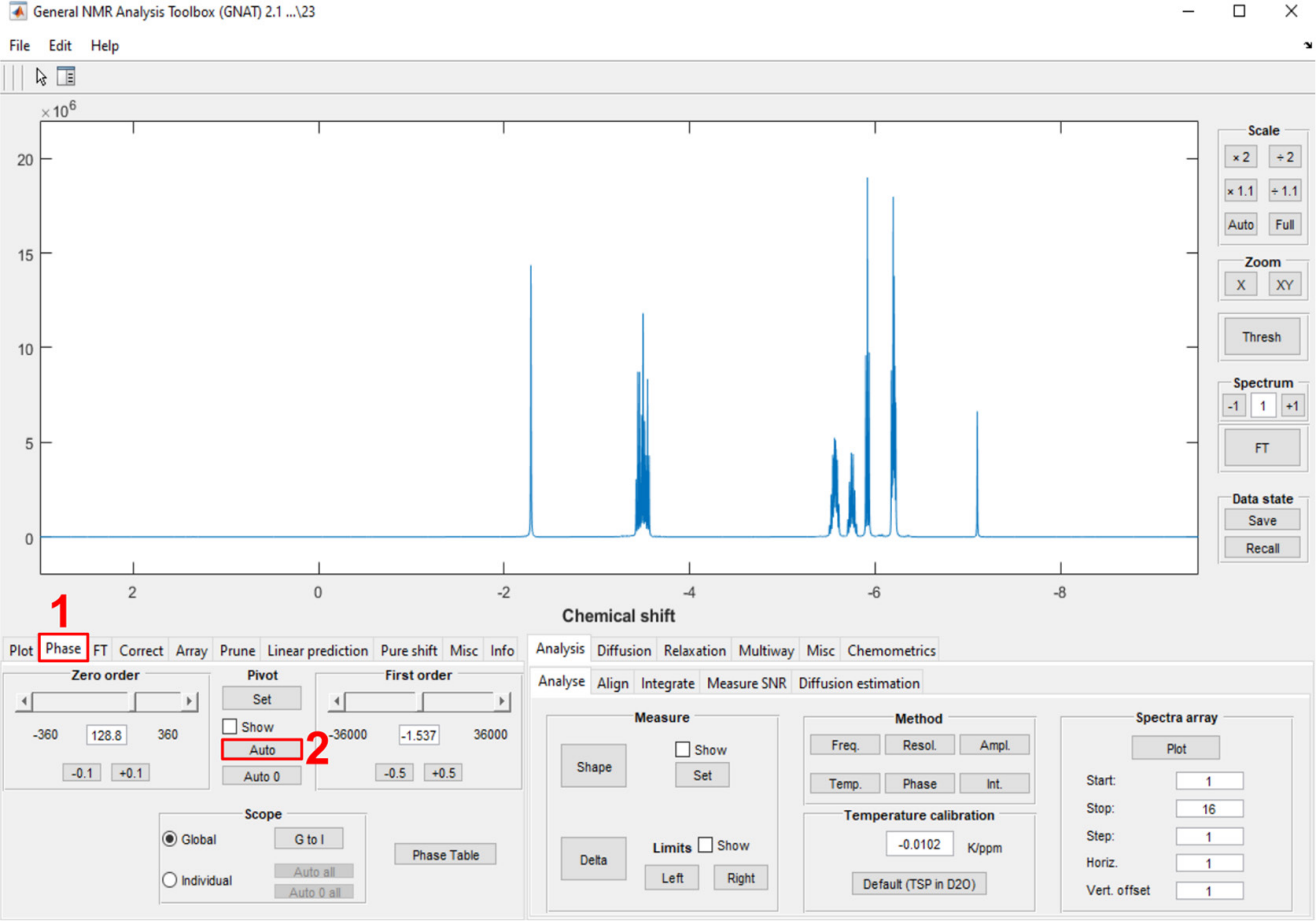
Automatic phase correction across all diffusion increments.

**FIGURE 5:**
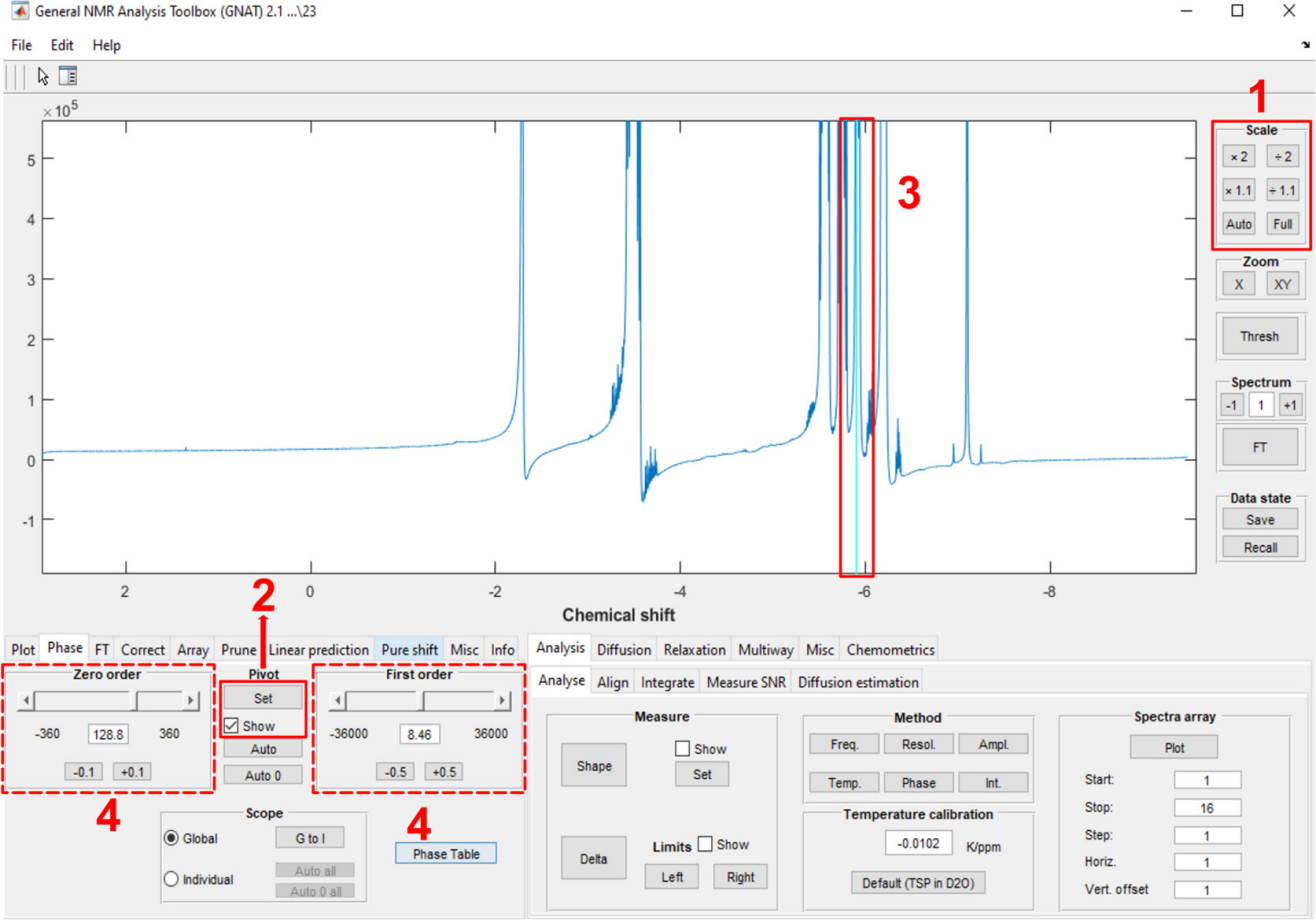
Manual phase correction applied across all diffusion increments.

**FIGURE 6:**
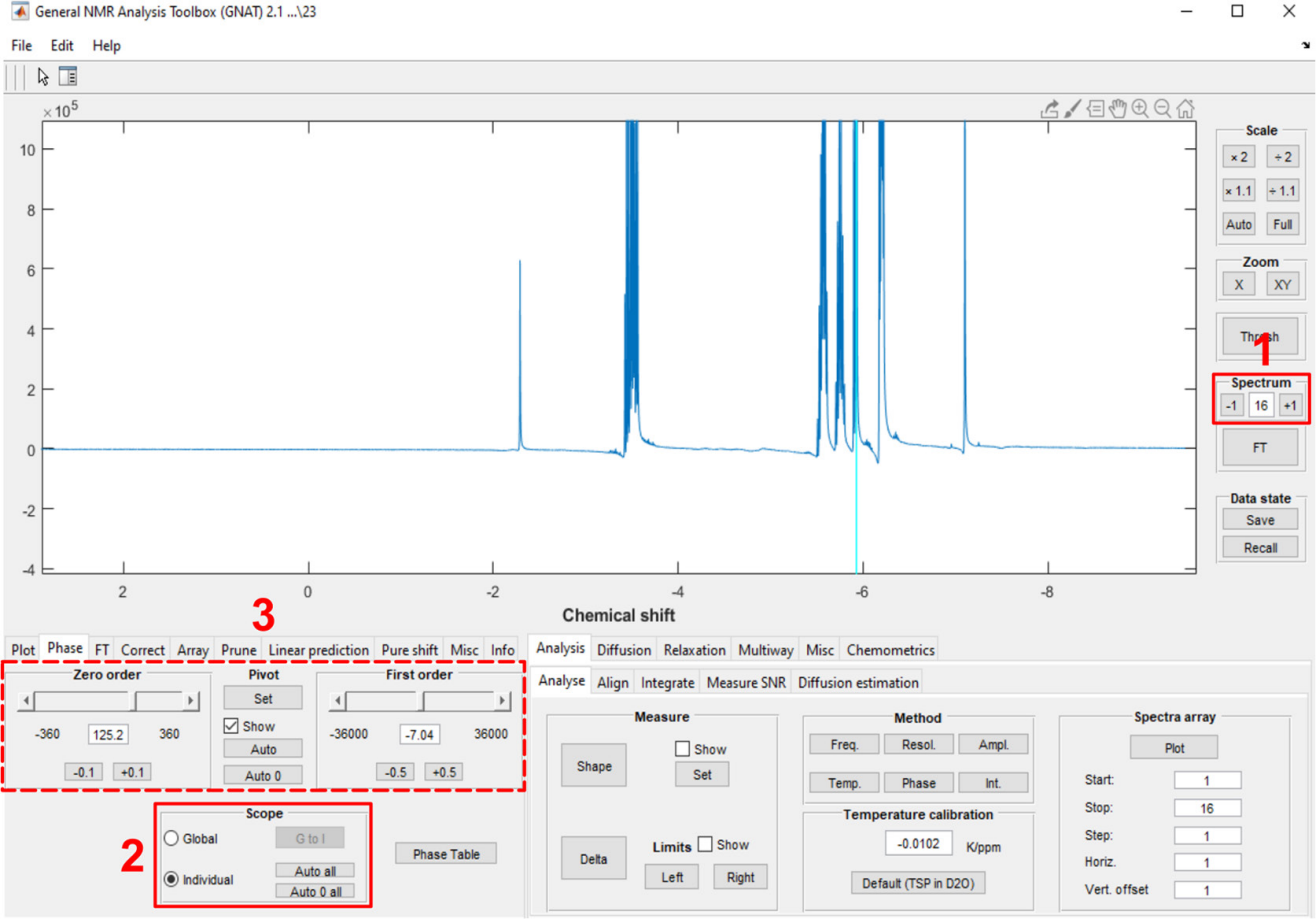
Manual phase correction applied individually to each gradient increment.

A detailed step‐by‐step guide for phase correction, with corresponding illustrations, is shown in Figures 4, 5, 6.

- Automatic phase: Open the *Phase* panel → click *Auto*.
- Additional manual phase correction is often needed: In the *Pivot* field, click *Set* to define the phase pivot point.
- Adjust the *Zero order* and *First order* phase parameter using the corresponding arrow buttons or by entering values directly into the numerical input fields.
- Next step is to adjust the individual gradient levels, moving between different increments in the *Array* tab (or in the shortcut—right part of the GUI). Click *G to I* to copy the Global phase parameters to all the individual gradient levels. Select the *Individual* mode in the *Scope* area. Adjust the zero and first order phase for each gradient increment individually.

### 3.5 Baseline Correction

**FIGURE 7:**
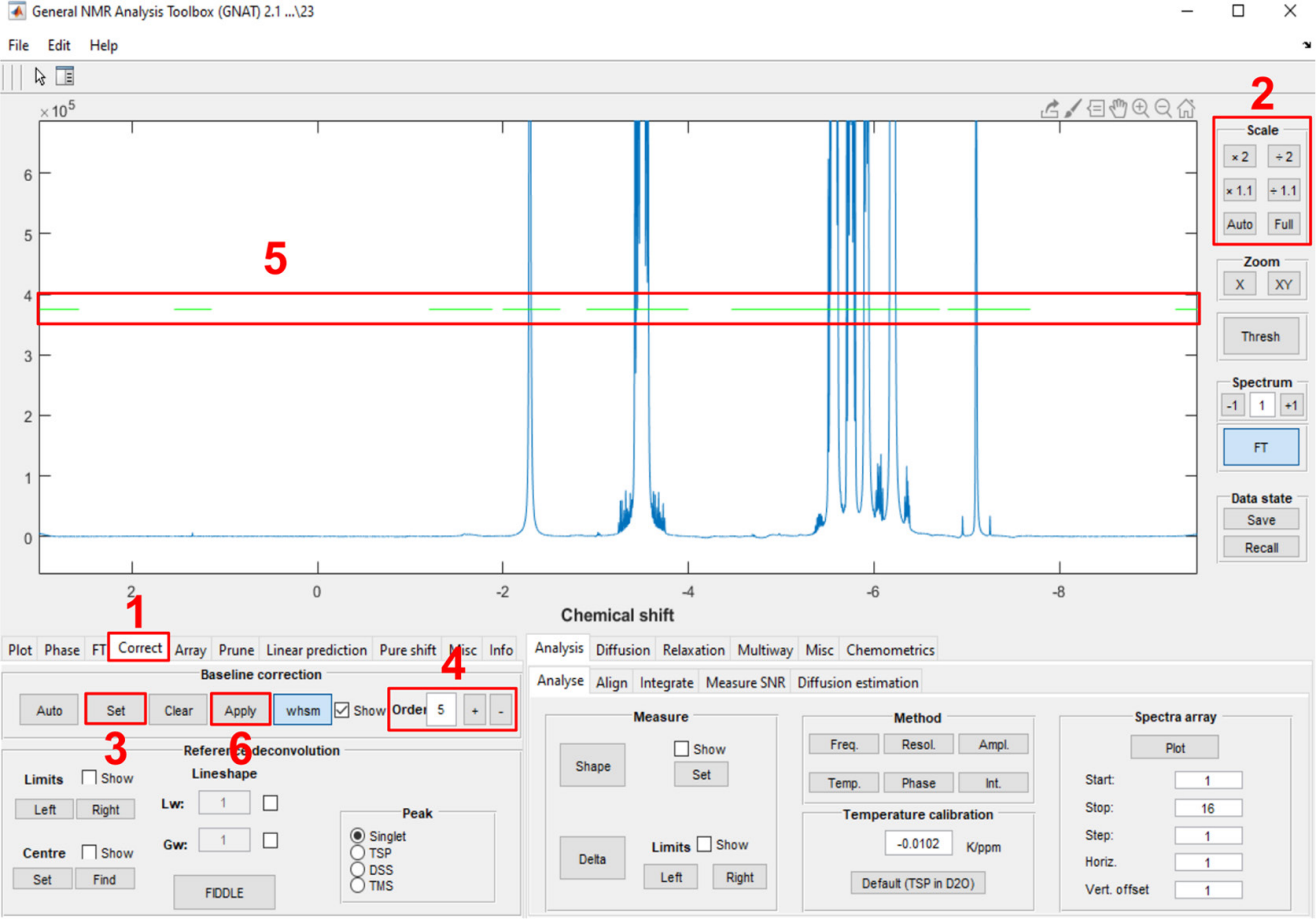
Manual baseline correction.

Correcting the baseline is the next step in the processing workflow. If the baseline is not flat or offset, the diffusion coefficients will be distorted because the fitting assumes a zero baseline offset. In a multiplet, this can manifest as different diffusion coefficients for different multiplet components. More details can be found in the Key Concepts section. The baseline correction procedure is described below and illustrated in Figure 7.

- Navigate to the *Correct* panel.
- Re‐scale the spectrum to visualize the baseline clearly.
- Select the *Order* for the baseline correction. This decides which order of polynomial is to be used (3–5 is often ideal)
- Click on *Set* to define the baseline.
- On the spectrum, select regions that represent the baseline. Define the baseline by clicking points from left to right where the baseline is presented. It is often useful to define the edges of the spectrum as peaks rather than a baseline because of the potential steep curvature in those regions due to modern digital signal processing.
- Click *Apply* to perform the correction.
- The automatic baseline correction can also be performed by selecting the desired order and clicking in *Auto* to apply the correction.

### 3.6 Chemical Shift Referencing

**FIGURE 8:**
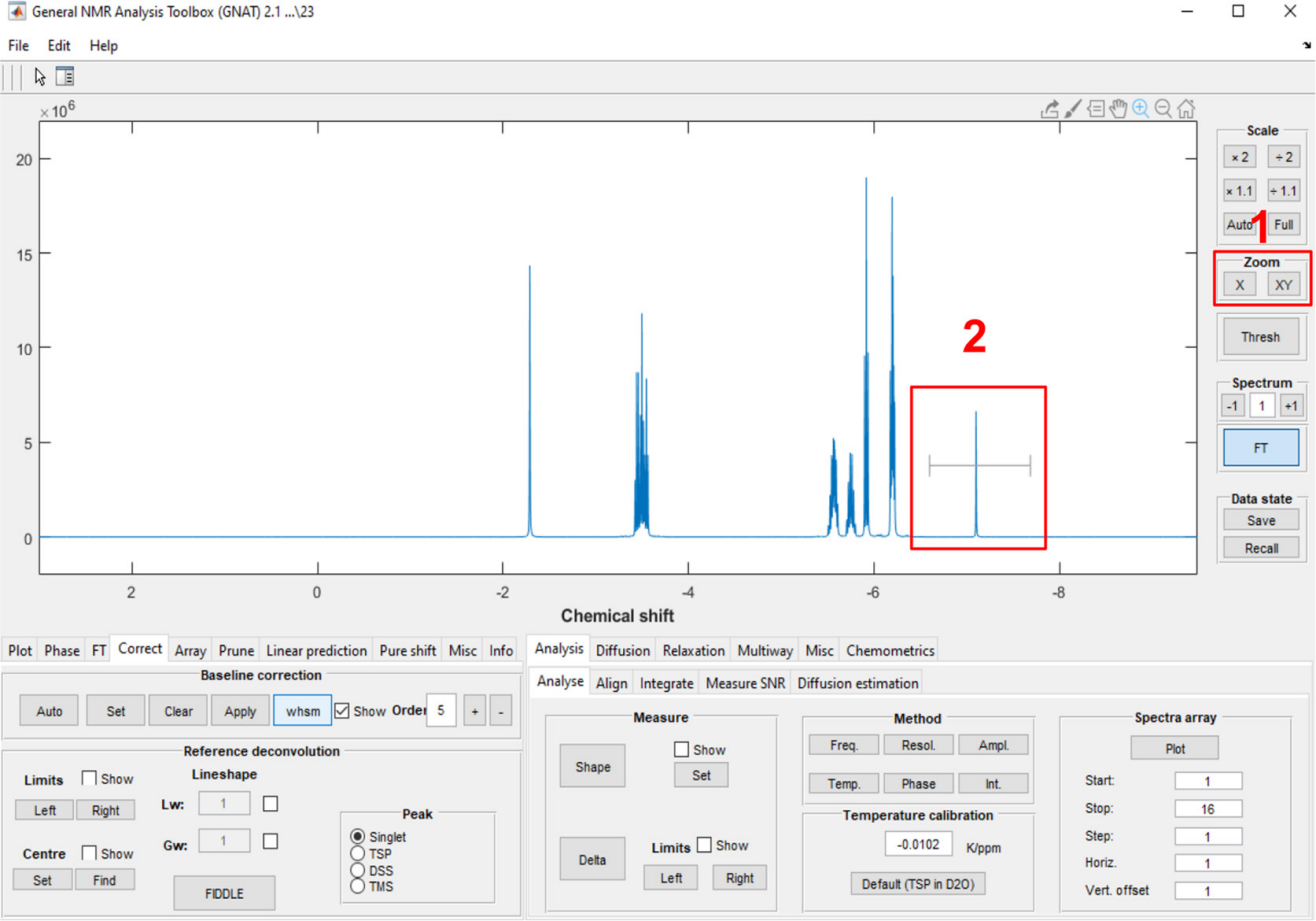
Expanding the reference signal region (red square in the spectrum).

**FIGURE 9:**
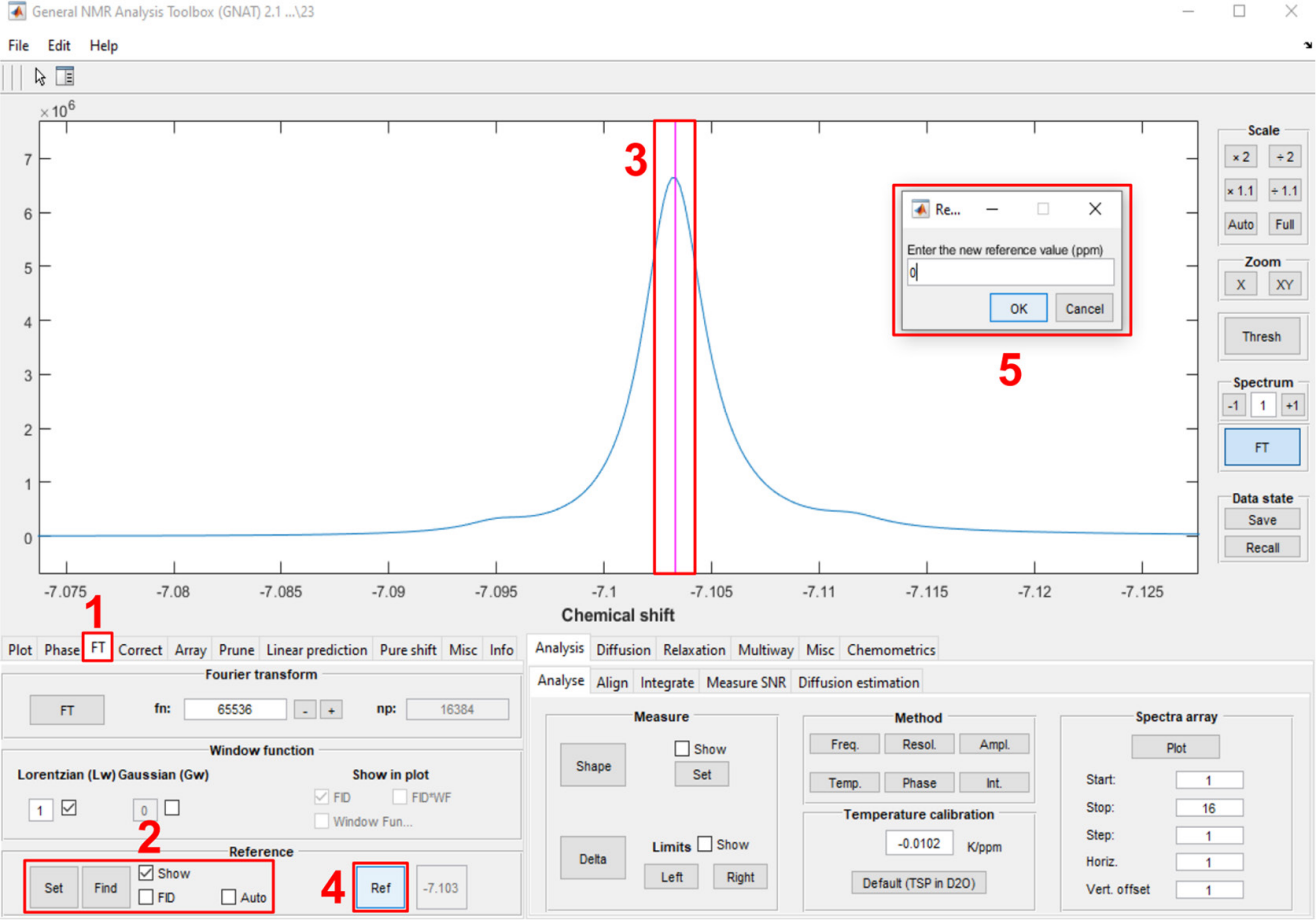
Referencing TMS to 0 ppm.

A procedure for referencing is described below and illustrated in Figures 8 and 9.

- Use the *Zoom* tool to expand the region with the reference peak (e.g., TMS or TSP).
- Open the *FT* panel and click *Set* to select the center of the reference signal (the *Find* button helps to find the peak maximum).
- Press the *Ref* button to set the chemical shift (in this example, 0.00 ppm).

### 3.7 Reference Deconvolution (Optional)

When possible, the application of reference deconvolution to NMR data is recommended [67, 68, 79], as it can help to correct experimental imperfections, such as lineshape distortions and phase errors, thereby improving the accuracy and reliability of the diffusion analysis.

**FIGURE 10:**
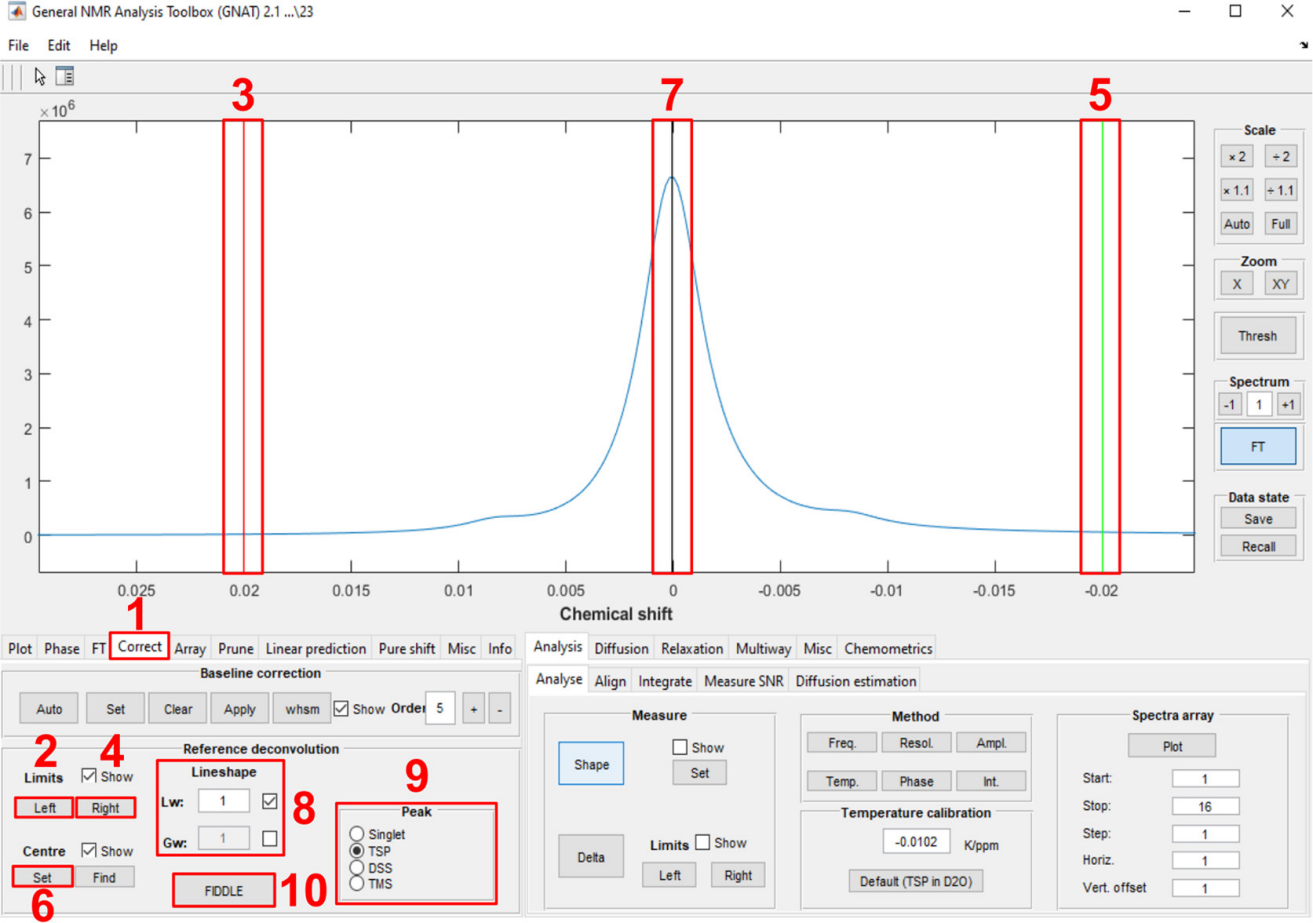
Step‐by‐step process of reference deconvolution.

The reference signal (TMS, TSP, or DSS) or a well‐resolved singlet should have a good SNR across all diffusion increments (rule of thumb: similar to or more than the compounds of interest). Note that for slowly diffusing species, it can be difficult to find a good reference compound. If a suitable signal is present, proceed with the steps outlined below and illustrated in Figure 10:

- Expand the region with the reference peak (e.g., TMS, TSP, or a singlet).
- In the *Correct* panel, click the *Left* and *Right* buttons to select the limits of the reference signal and then click the *Set* button to select the center. The reference region should include the whole peak and some baseline. If the selection is too narrow and includes a significant part of the reference signal without any baseline, the peak shapes obtained after deconvolution will be distorted. If too much baseline is included, it deteriorates the SNR of the deconvoluted spectrum. If the reference peak is narrow, the region can be set inside any ^13^C satellites; otherwise, the satellites are accounted for in the algorithm.
- Choose the desired line shape using the *Lw/Gw* input boxes. In the example presented, a 1‐Hz Lorentzian shape was applied.
- In the *Peak* field, select the appropriate reference signal type for your spectrum.
- Click *FIDDLE* to perform deconvolution.

Using reference deconvolution, the user chooses the final lineshape, but the same rules as for resolution and sensitivity enhancement with windows functions apply; that is, it is a trade‐off between sensitivity and resolution. So, if one is too greedy with the resolution, the resulting spectrum will be noisy. For reference deconvolution, the effect of noise deconvoluted from the reference peak becomes apparent. Trial and error, examining all gradient levels to ensure they all appear acceptable, is a practical approach. This is further discussed in Key Concepts.

Before proceeding to the next section, note that in the Peak tab, the selected option determines the reference signal used by the FIDDLE algorithm. When “TSP” is selected, the user manually chooses the experimental TSP signal, which is converted to the time domain via inverse FT and combined with an ideal synthetic FID to generate a reference‐deconvoluted FID, thereby suppressing experimental imperfections before transformation back to the spectral domain. The same procedure applies when “singlet, DSS or TMS” is selected.

### 3.8 Generating the DOSY Plot

**FIGURE 11:**
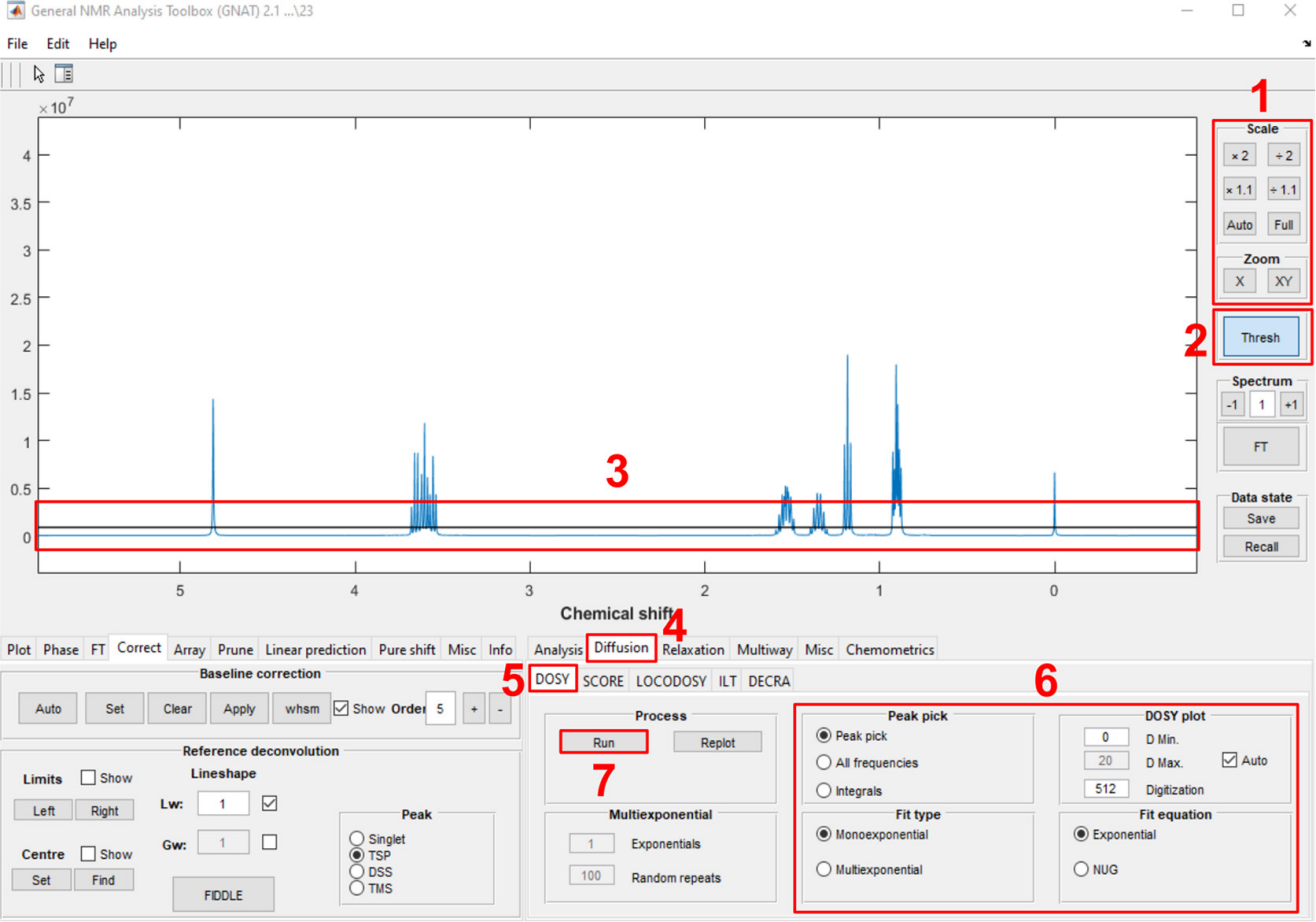
Steps for generating the DOSY plot.

A common procedure for generating a DOSY plot is described below and illustrated in Figure 11. More details about advanced options can be found in the GNAT manual [66].

**FIGURE 12:**
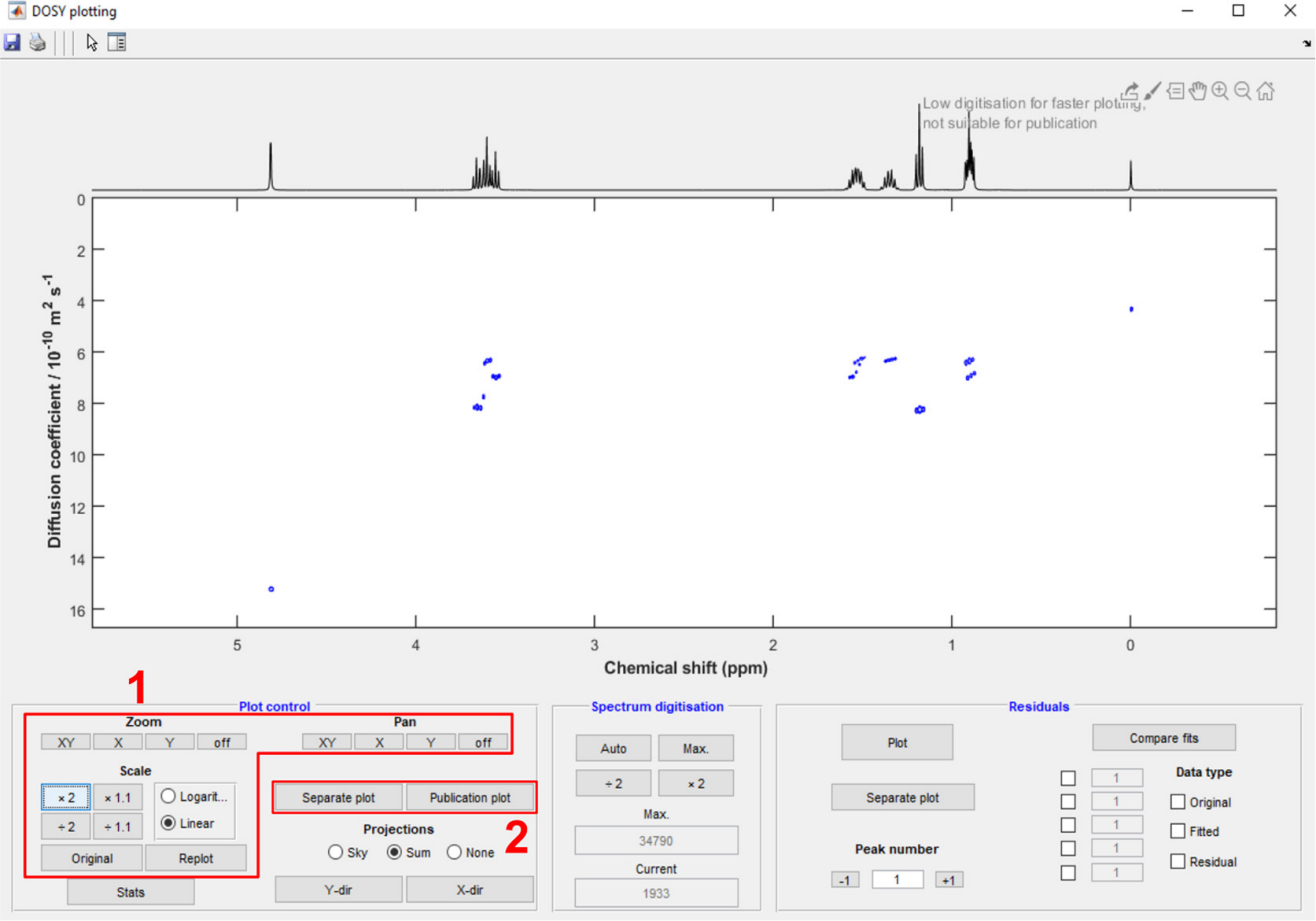
Functions to adjust the appearance of the DOSY plot, as well as options to generate a separate plot for reports and publications.

- Use the *Scale/Zoom* tools to navigate to the region of interest in the spectrum.
- Click on *Thresh* and set a threshold in the spectrum. Only signals above this line will be considered for diffusion fitting.
- Open the *Diffusion* panel and then click on the *DOSY* subpanel.
- Choose *Peak Pick* as the analysis method. As fitting options, select *Monoexponential* as the fitting type and *Exponential* under the *Fit Equation* field. These settings typically provide a reliable estimation of the diffusion coefficients; however, users can also explore alternative choices, such as *All Frequencies* or *Integrals*, as well as different fitting approaches (e.g., *multiexponential fitting* or *NUG*), all of which are available in GNAT, and described in the manual.
- Click *Run* to generate the DOSY plot. A new GUI window will open (illustrated in Figure 12), displaying the resulting diffusion plot.
- Note that the pulse sequence type, gradient shape, and relevant parameters (e.g., delays, constants, and gradient ramp) used for the estimation of diffusion coefficients can be verified by navigating to *Edit* (left‐hand side of the GNAT window) → *Settings*. This opens the settings window, where, in the Diffusion tab, diffusion‐related parameters can be viewed and modified if necessary. Additionally, the identified pulse sequence and extracted parameters are displayed in the MATLAB command window upon importing the experiment into GNAT, allowing the user to confirm that the information has been correctly recognized.

### 3.9 Analyzing the DOSY Plot

#### 3.9.1 General Plot Preparation

**FIGURE 13:**
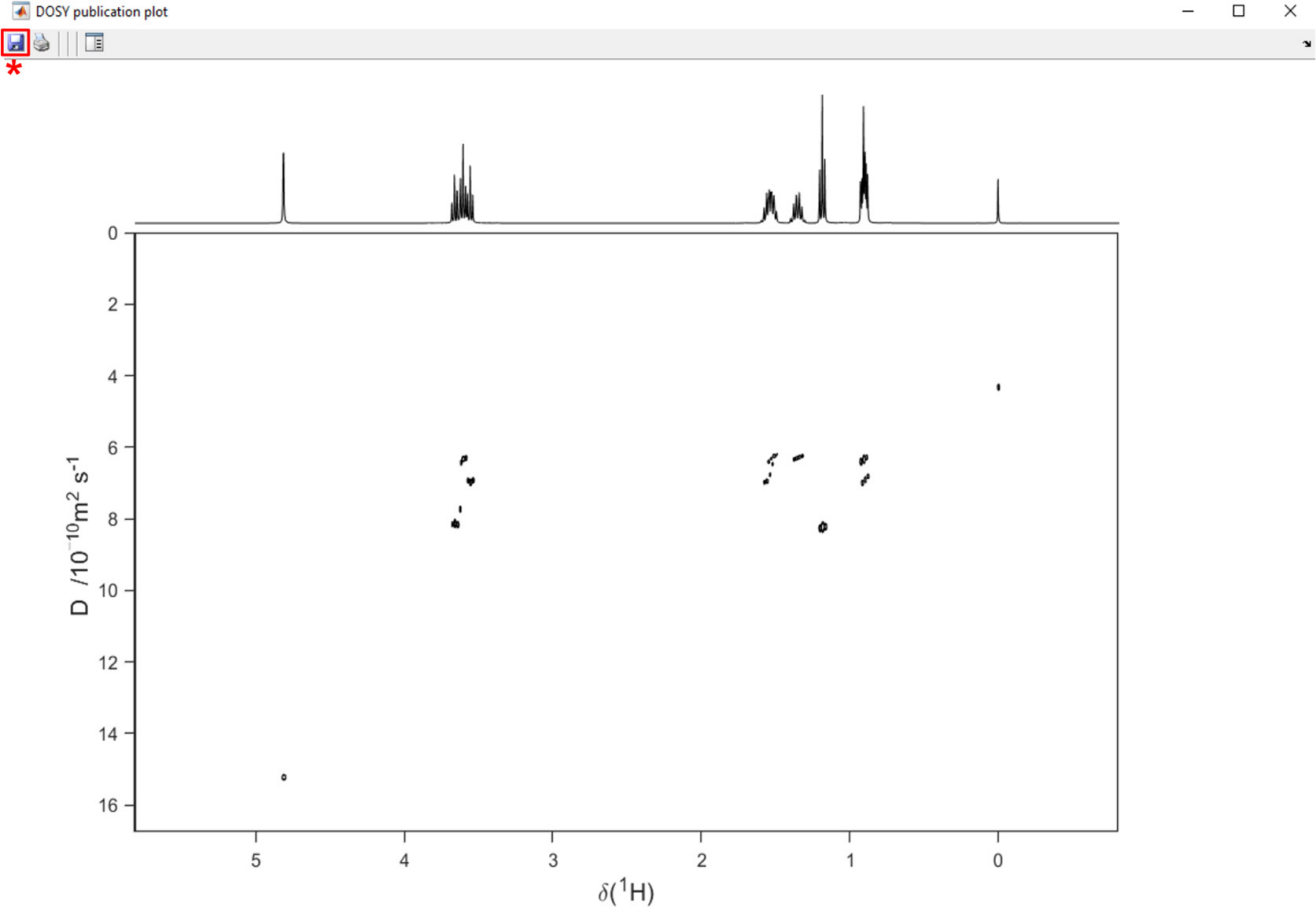
A DOSY plot in publication format. The disk icon (highlighted) is used to save and export the diffusion plot in multiple file formats.

**FIGURE 14:**
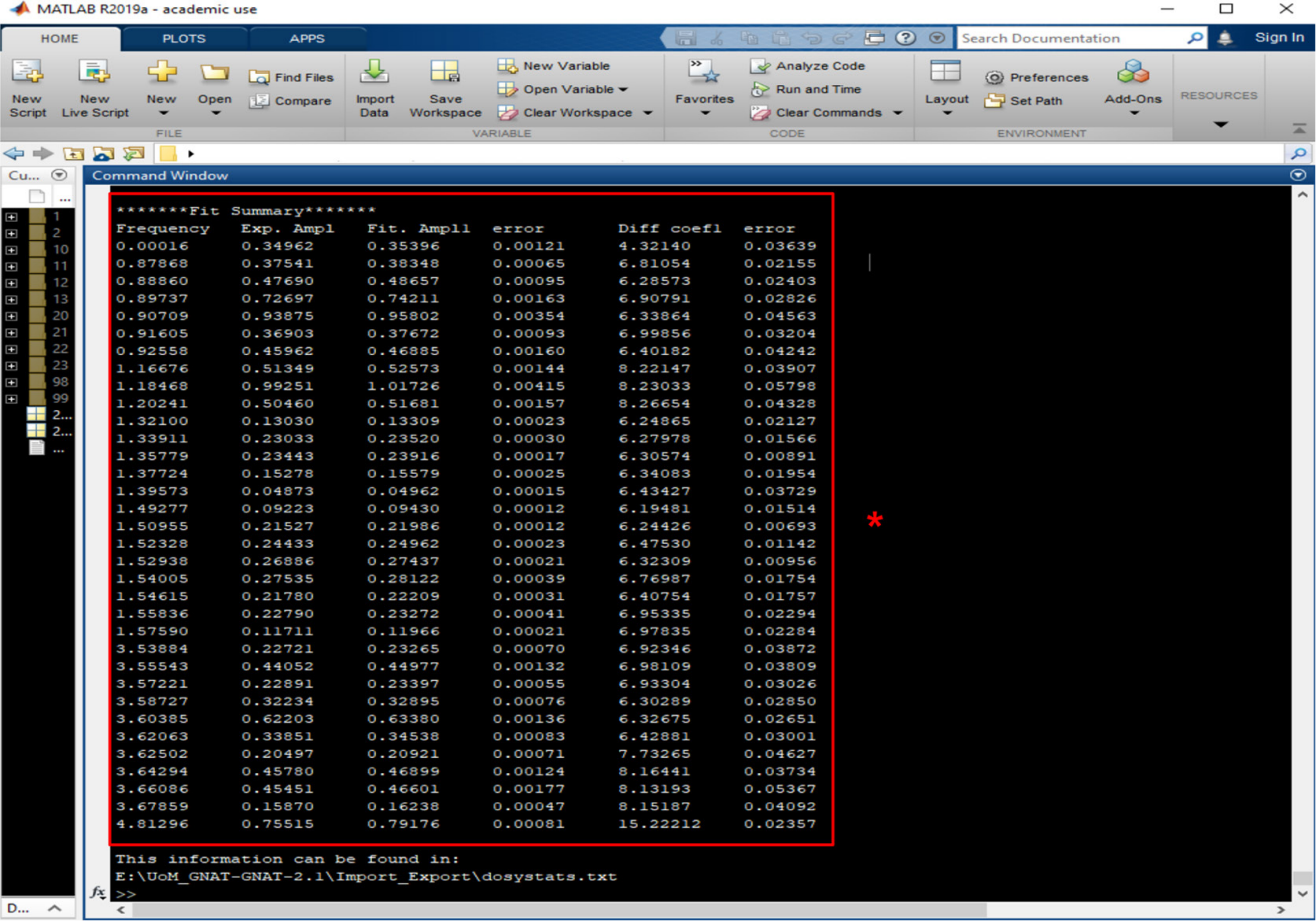
Terminal display of diffusion coefficients and statistical results from the fitting process.

The DOSY plot is accessed in a separate GUI, where diffusion coefficients and associated statistical parameters can also be inspected by following the steps outlined below and illustrated in Figures 12, 13, 14. Contour plots can be rendered slowly, so the plot data is downscaled to match the screen resolution better. If the (automatic) downscaling is too severe, the spectra can become distorted, in which case a higher digitization should be chosen.

- Use the *Zoom/Scale/Pan* controls to adjust the visual appearance of the DOSY plot (as shown in Figure 12).
- Click on *Publication Plot* or *Separate Plot* to open a version of the DOSY plot suitable for publishing or sharing (Figure 12).
- The user can (as shown in Figure 13) save and export the diffusion plot in multiple formats (e.g., *pdf*, *png*, and svg) by clicking the *disk icon* located at the top‐left corner of the window.
- Individual diffusion coefficients and associated fit statistics are displayed in the terminal of the program (as depicted in Figure 14). These can also be exported by clicking *Stats* under the *Plot* controls of the DOSY Plotting tab (as presented in Figure 12).

#### 3.9.2 Fitting Analysis

**FIGURE 15:**
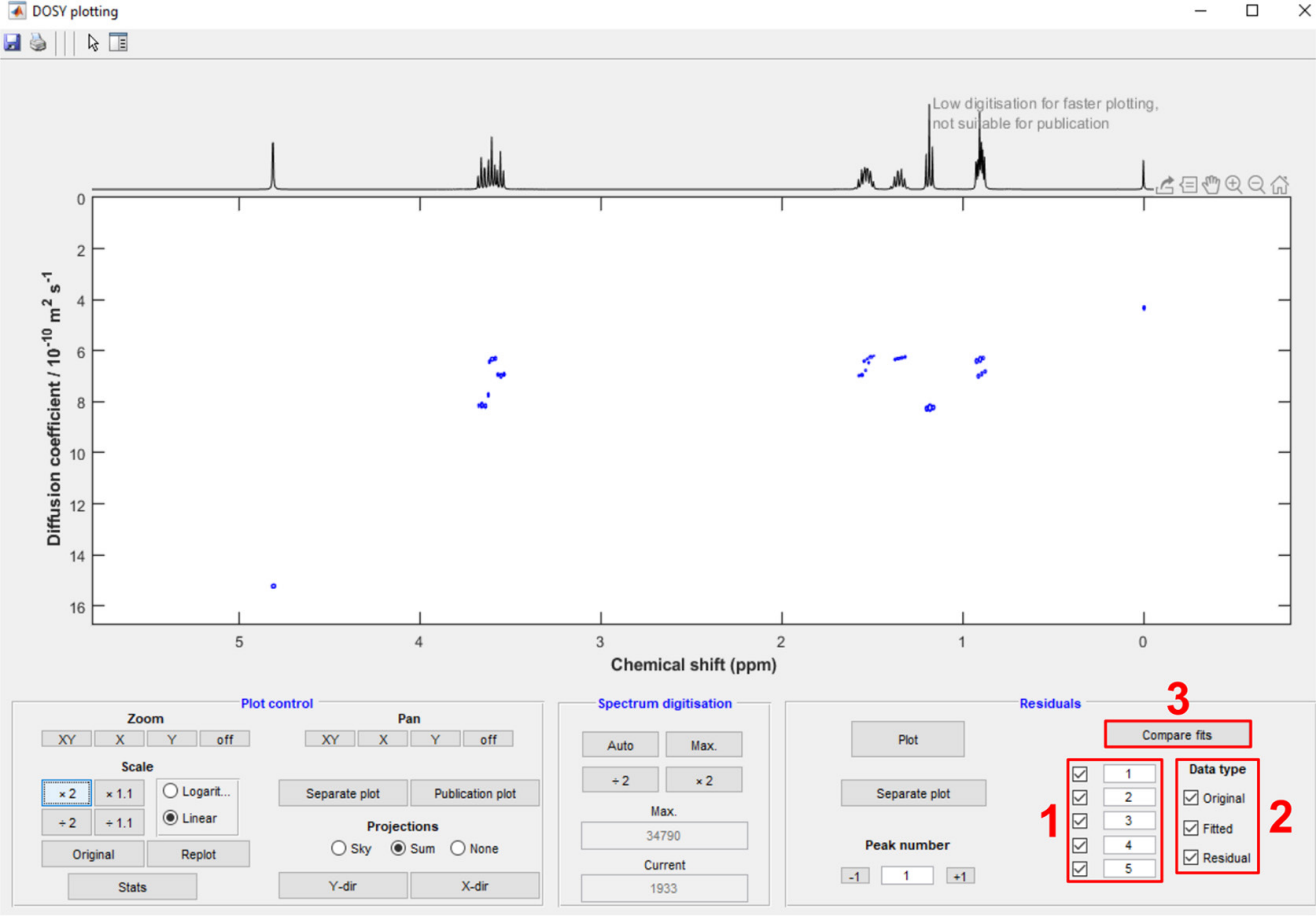
Access to plots with further information on the diffusion fitting.

**FIGURE 16:**
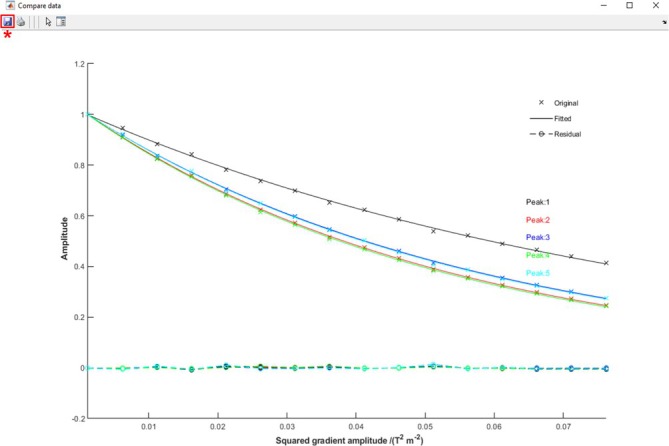
Fits and residuals for individual peaks.

The DOSY GUI provides detailed plots of the fits and residuals to help evaluate the quality and reliability of the diffusion fitting. The plots are accessed by following the steps below (also illustrated in Figures 15 and 16):

- In the *Residuals* section, enable the *checkboxes* and enter the number corresponding to the signal of interest.
- Activate the desired selection of *Original*, *Fitted*, and *Residual* in the *Data type* area, and press *Compare Fits* to display the results (Figure 16).

The presented procedure is a simple standard protocol for processing diffusion‐NMR experiments using GNAT and should be similar to those in other software packages. While alternative processing approaches may be valid and even preferable in specific contexts, this guide provides a solid starting point, especially for beginners. We encourage users to explore different processing strategies based on their acquisition conditions and the complexity of the sample. Processing choices may vary, particularly when dealing with overlapping peaks, noisy spectra, or complex mixtures of components, but this is a vast topic and beyond the scope of this tutorial.

## 4 Key Concepts, Best Practices, and Common Pitfalls

In general, common mistakes during diffusion‐NMR data processing potentially lead to inaccurate diffusion coefficients and/or misinterpretation of results. Being aware of these pitfalls is essential to ensure reliable and reproducible analysis.

Processing choices can significantly influence not only the absolute values of the diffusion coefficients but also their relative trends. This section highlights critical aspects of DOSY data processing, with particular focus on the impact of phase and baseline correction, as well as the choice of window functions, all of which can strongly affect the quality, accuracy, and reliability of the final diffusion data.

### 4.1 Phase Correction

Inaccurate phase correction can introduce significant errors in the estimated diffusion values and lead to incorrect interpretation. Automatic phase correction often works less well for diffusion data, as the spectra often suffer from *J*‐modulation, which is not accounted for in most phasing algorithms (including the one in GNAT). It is common practice to apply phase correction globally, across all gradient increments simultaneously. However, this approach does not consider phase variations that can occur between individual increments. In many diffusion‐NMR experiments, there is an inherent gradient‐dependent phase error that requires correction for reliable results. See the Supporting Information (Section 5) for further information on phase correction.

These residual phase inconsistencies can compromise the accuracy of the extracted diffusion coefficients and, perhaps even more critically, may lead to misinterpretations of the diffusion behavior, particularly in systems where the differences in diffusion coefficients are subtle, such as in a mixture of isomeric compounds [4, 5].

To address this issue, phase correction should be applied individually to each increment of the diffusion experiment. This is often corrected automatically when using reference deconvolution.

### 4.2 Baseline Correction

The effects of a curved and/or offset baseline in the DOSY analysis manifest as erroneous diffusion coefficients, because the standard fitting assumes a zero baseline. It would be possible to include a baseline offset as a fitting parameter, but this increases the uncertainty in the fitted parameters, and it is almost always preferable to perform a baseline correction (see Supporting Information—Section 6 for further details on baseline correction methods). The errors generally come in three forms: (i) too high or too low diffusion coefficients, (ii) signals from the same compound can report different diffusion coefficients, and (iii) different multiplet components (from a single compound) report different diffusion coefficients.

### 4.3 Window Functions and Reference Deconvolution

In NMR, we typically have some freedom to choose the lineshape in our final spectrum. Most commonly, this is done by applying window functions to the FID. There is a tradeoff between sensitivity (SNR) and resolution, and the best compromise depends on the particular problem one is investigating. In DOSY, the choice of lineshape can make a big difference to the resulting plot and its interpretation. The higher the SNR, the better data fit (to a practical limit [80]), as long as the signals are resolved, but having resolved signals is important for a simple interpretation.

The experimental line shape depends on spectrometer stability and magnetic field homogeneity (shimming). Many of these discrepancies can be corrected using reference deconvolution (RD), in which the lineshape errors are deduced from a reference signal [67, 68, 69, 79]. As part of the reference deconvolution process, the user has the freedom to set the line shape analogously to using window functions. If a suitable reference compound is present, it is preferable to use RD to correct the lineshape and decide the compromise between resolution and sensitivity (see Supporting Information—Sections 4 and 7–9).

**FIGURE 17:**
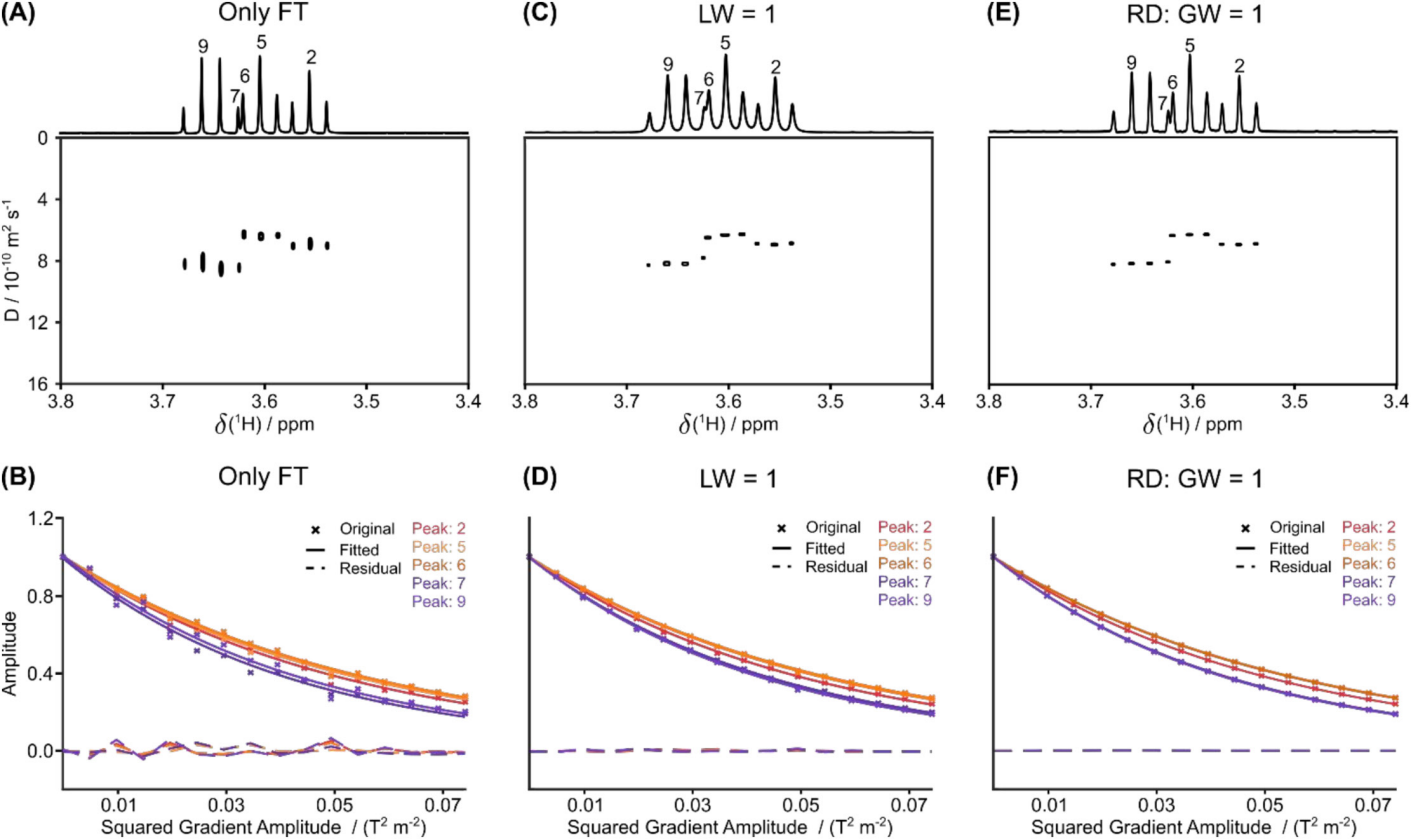
DOSY plots (top) and corresponding signal fitting plots (bottom) for a mixture of ethanol, propanol, and butanol in D_2_O, focusing on the spectral region from 3.4 to 3.8 ppm, processed using three different methods: (A,B) Fourier transform only; (C,D) Lorentzian window function with 1‐Hz line broadening; and (E,F) reference deconvolution to a target lineshape a 1‐Hz Gaussian. The highlighted signals correspond to specific fitted peaks: Peak 2 (propanol), Peaks 5 and 6 (butanol), and Peaks 7 and 9 (ethanol).

The effect of different window functions and RD is shown in Figure 17. A more comprehensive comparison is available in the Supporting Information (Sections 4, 7, and 8). Figure 17A,B shows the resulting DOSY plot and the fitting data with the raw experimental line width. It is still interpretable for an experienced spectroscopist, but there is room for improvement: the fit error is higher than necessary, resulting in broad peaks (in the diffusion dimension) and a spread of fitted diffusion coefficients. By applying a 1‐Hz Lorentzian line broadening, we see in Figure 17C,D that the fit errors have improved significantly due to the increase in S/N (1‐Hz line broadening here is close to matched filtration, which gives maximum S/N—see Section 7 in the Supporting Information). However, the peak positions are still somewhat confusing in the diffusion dimension. The overlap around 3.62 ppm causes the overlapping multiplet components to have diffusion coeffects in between those of the two involved alcohols. A better result is seen in Figure 17E,F, where a combination of resolution enhancement and RD has been used. The overlap has now been resolved, and the more consistent line shapes (across the gradient increments) afforded by the RD significantly reduced the fit error.

**FIGURE 18:**
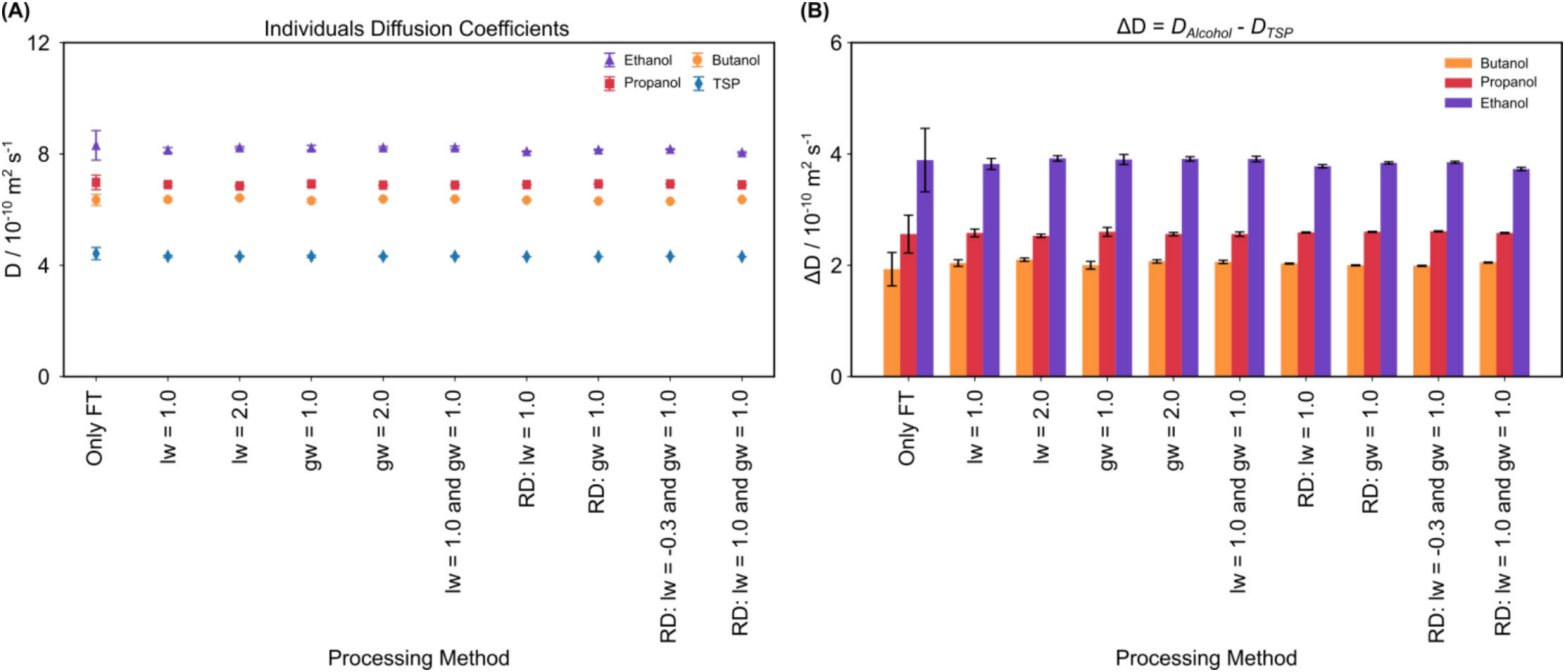
(A) Diffusion coefficients of ethanol, propanol, and butanol in their mixture in deuterated water, along with their associated errors across 10 different processing methods. (B) The trends in the relative diffusion differences (*ΔD*_ethanol, propanol, or butanol_ = *D*_ethanol, propanol, or butanol_ − *D*_TSP_) and corresponding propagated errors between ethanol and TSP, propanol and TSP, and butanol and TSP across the different processing methods.

Figure 18 gives an overview of different processing strategies (more detailed information in the Supporting Information—Sections 4 and 8), where the ability to discriminate between different compounds is illustrated. Using the raw experimental line shape (only FT in Figure 18) yields the highest errors in the estimated diffusion coefficients. The inclusion of a window function improves the accuracy of these estimations, particularly in datasets with lower SNRs, and in such cases, increasing the line broadening tends to minimize uncertainty further. However, above a certain signal‐to‐noise threshold, the improvement becomes minimal, and it should be noted that systematic errors, such as those from convection, are not improved and corrected by line broadening.

Moreover, individual diffusion coefficients may fluctuate across different processing conditions (Figure 18A). In this context, if the primary goal is not the accurate determination of absolute self‐diffusion coefficients but rather the resolution of complex mixtures (as is often the case), we recommend focusing on diffusion trends (e.g., the relative differences between the compounds' diffusion coefficients) rather than the absolute values themselves. It is advisable to average the diffusion coefficients across multiple signals of the same compound whenever possible, propagate their associated errors accordingly, and then analyze the trends.

As shown in Figure 18B, the diffusion trends remain consistent across different processing conditions. For example, the differences in diffusion between each alcohol and the reference compound are qualitatively similar regardless of the chosen processing method, and only the errors are minimized. It is worth noting that the reference deconvolution results showed the lowest associated and propagated errors, thereby significantly improving resolution in DOSY experiments and enhancing the reliability and accuracy of compound separation in complex mixture analysis.

## 5 Conclusion

This tutorial has presented practical guidance on processing diffusion‐NMR data using GNAT, with an emphasis on supporting researchers who are new to diffusion experiments. Our aim is to help users process DOSY data with confidence and obtain reproducible and accurate results. While the discussion focused on DOSY approaches for resolving complex mixtures, the workflow, quality checks, and optimization strategies described are broadly relevant across diffusion‐NMR applications. By highlighting common pitfalls and outlining best practices, we hope to promote the routine generation of high‐quality DOSY spectra with reliable diffusion coefficients and consistent trends. More broadly, we anticipate that this tutorial will support both academic and industrial users by encouraging standardized, robust processing of diffusion data and contributing to the widespread, reliable adoption of diffusion NMR within the community.

## Author Contributions

**Tadeu Luiz Gomes Cabral:** conceptualization, data curation, formal analysis, investigation, methodology, visualization, writing – original draft. **Guilherme Dal Poggetto:** conceptualization, formal analysis, investigation, methodology, visualization, software, writing – review and editing. **Claudio F. Tormena:** conceptualization, formal analysis, funding acquisition, methodology, project administration, resources, supervision, validation, writing – review and editing. **Mathias Nilsson:** conceptualization, formal analysis, software, funding acquisition, methodology, project administration, resources, validation, writing – review and editing.

## Funding

This work was supported by Fundação de Amparo à Pesquisa do Estado de São Paulo (2020/10246‐0, 2021/05081‐5, 2023/07116‐6, 2022/11152‐5); LIRMN (RRID: SCR_027247); CEMUIQ‐UNICAMP; and the Engineering and Physical Sciences Research Council (EP/E057888/1, EP/E05899X/1, EP/R018790/1, EP/X035476/1, EP/Z534201/1).

## Conflicts of Interest

The authors declare no conflicts of interest.

## Supporting information


**Figure S1:** Importing diffusion‐NMR data into GNAT.
**Figure S2:** Application of a Fourier transformation.
**Figure S3:** Automatic phase correction.
**Figure S4:** Manual global phase correction.
**Figure S5:** Individual phase correction across diffusion increments.
**Figure S6:** Manual baseline correction.
**Figure S7:** Selection of reference signal region.
**Figure S8:** Chemical shift referencing.
**Figure S9:** Reference deconvolution workflow.
**Figure S10:** Generation of DOSY plot.
**Figure S11:** DOSY visualization interface.
**Figure S12:** Exporting DOSY plots.
**Figure S13:** Terminal displaying the diffusion coefficients and fitting statistics.
**Figure S14:** Access to further information on the fitting results.
**Figure S15:** Fits and residuals for selected peaks.
**Figure S16: A)** 400 MHz ^1^H‐DOSY plots and **B)** corresponding signal fitting plots for a mixture of ethanol, propanol, and butanol in D_2_O, focusing on the spectral region from 3.4 to 3.8 ppm, processed without any window function. In all cases, manual phasing for each increment and manual baseline correction were applied.
**Figure S17: A)** 400 MHz ^1^H‐DOSY plots and **B)** corresponding signal fitting plots for a mixture of ethanol, propanol, and butanol in D_2_O, focusing on the spectral region from 3.4 to 3.8 ppm, processed with a Lorentzian window function of 1 Hz. In all cases, manual phasing for each increment and manual baseline correction were applied.
**Figure S18: A)** 400 MHz ^1^H‐DOSY plots and **B)** corresponding signal fitting plots for a mixture of ethanol, propanol, and butanol in D_2_O, focusing on the spectral region from 3.4 to 3.8 ppm, processed with a Lorentzian window function of 2 Hz. In all cases, manual phasing for each increment and manual baseline correction were applied.
**Figure S19: A)** 400 MHz ^1^H‐DOSY plots and **B)** corresponding signal fitting plots for a mixture of ethanol, propanol, and butanol in D_2_O, focusing on the spectral region from 3.4 to 3.8 ppm, processed with a Gaussian window function of 1 Hz. In all cases, manual phasing for each increment and manual baseline correction were applied.
**Figure S20: A)** 400 MHz ^1^H‐DOSY plots and **B)** corresponding signal fitting plots for a mixture of ethanol, propanol, and butanol in D_2_O, focusing on the spectral region from 3.4 to 3.8 ppm, processed with a Gaussian window function of 2 Hz. In all cases, manual phasing for each increment and manual baseline correction were applied.
**Figure S21: A)** 400 MHz ^1^H‐DOSY plots and **B)** corresponding signal fitting plots for a mixture of ethanol, propanol, and butanol in D_2_O, focusing on the spectral region from 3.4 to 3.8 ppm, processed with a combined Lorentzian and Gaussian window functions of 1 Hz. In all cases, manual phasing for each increment and manual baseline correction were applied.
**Figure S22: A)** 400 MHz ^1^H‐DOSY plots and **B)** corresponding signal fitting plots for a mixture of ethanol, propanol, and butanol in D_2_O, focusing on the spectral region from 3.4 to 3.8 ppm, processed with reference deconvolution based on a Lorentzian window function of 1 Hz. In all cases, manual phasing for each increment and manual baseline correction were applied.
**Figure S23: A)** 400 MHz ^1^H‐DOSY plots and **B)** corresponding signal fitting plots for a mixture of ethanol, propanol, and butanol in D_2_O, focusing on the spectral region from 3.4 to 3.8 ppm, processed with reference deconvolution based on a Gaussian window function of 1 Hz. In all cases, manual phasing for each increment and manual baseline correction were applied.
**Figure S24: A)** 400 MHz ^1^H‐DOSY plots and **B)** corresponding signal fitting plots for a mixture of ethanol, propanol, and butanol in D_2_O, focusing on the spectral region from 3.4 to 3.8 ppm, processed with reference deconvolution based on a Lorentzian window function −0.3 Hz and a Gaussian window function of 1 Hz. In all cases, manual phasing for each increment and manual baseline correction were applied.
**Figure S25: A)** 400 MHz ^1^H‐DOSY plots and **B)** corresponding signal fitting plots for a mixture of ethanol, propanol, and butanol in D_2_O, focusing on the spectral region from 3.4 to 3.8 ppm, processed with reference deconvolution based on a combined Lorentzian window function and Gaussian window function of 1 Hz. In all cases, manual phasing for each increment and manual baseline correction were applied.
**Figure S26: A)** 400 MHz ^1^H‐DOSY plots and **B)** corresponding signal fitting plots for a mixture of ethanol, propanol, and butanol in D_2_O, focusing on the spectral region from 3.4 to 3.8 ppm, processed with a Lorentzian window function of 0.5 Hz. In all cases, manual phasing for each increment and manual baseline correction were applied.
**Figure S27: A)** 400 MHz ^1^H‐DOSY plots and **B)** corresponding signal fitting plots for a mixture of ethanol, propanol, and butanol in D_2_O, focusing on the spectral region from 3.4 to 3.8 ppm, processed with a Gaussian window function of 0.5 Hz. In all cases, manual phasing for each increment and manual baseline correction were applied.
**Figure S28: A)** 400 MHz ^1^H‐DOSY plots and **B)** corresponding signal fitting plots for a mixture of ethanol, propanol, and butanol in D_2_O, focusing on the spectral region from 3.4 to 3.8 ppm, processed with a combined Lorentzian window function of −0.5 Hz and Gaussian width of 0.5 Hz. In all cases, manual phasing for each increment and manual baseline correction were applied.
**Figure S29: A)** 400 MHz ^1^H‐DOSY plots and **B)** corresponding signal fitting plots for a mixture of ethanol, propanol, and butanol in D_2_O, focusing on the spectral region from 3.4 to 3.8 ppm, processed with a combined Lorentzian window function of 0.5 Hz and Gaussian width of 0.5 Hz. In all cases, manual phasing for each increment and manual baseline correction were applied.
**Figure S30: A)** 400 MHz ^1^H‐DOSY plots and **B)** corresponding signal fitting plots for a mixture of ethanol, propanol, and butanol in D_2_O, focusing on the spectral region from 3.4 to 3.8 ppm, processed using reference deconvolution based on a Lorentzian window function of 0.5 Hz. In all cases, manual phasing for each increment and manual baseline correction were applied.
**Figure S31: A)** 400 MHz ^1^H‐DOSY plots and **B)** corresponding signal fitting plots for a mixture of ethanol, propanol, and butanol in D_2_O, focusing on the spectral region from 3.4 to 3.8 ppm, processed using reference deconvolution based on a Gaussian window function of 0.5 Hz. In all cases, manual phasing for each increment and manual baseline correction were applied.
**Figure S32: A)** 400 MHz ^1^H‐DOSY plots and **B)** corresponding signal fitting plots for a mixture of ethanol, propanol, and butanol in D_2_O, focusing on the spectral region from 3.4 to 3.8 ppm, processed using reference deconvolution based on a combined Lorentzian window function of −0.5 Hz and Gaussian width of 0.5 Hz. In all cases, manual phasing for each increment and manual baseline correction were applied.
**Figure S33: A)** 400 MHz ^1^H‐DOSY plots and **B)** corresponding signal fitting plots for a mixture of ethanol, propanol, and butanol in D_2_O, focusing on the spectral region from 3.4 to 3.8 ppm, processed using reference deconvolution based on a combined Lorentzian window function of 0.5 Hz and Gaussian width of 0.5 Hz. In all cases, manual phasing for each increment and manual baseline correction were applied.
**Figure S34:** DOSY plots for a mixture of ethanol, propanol, and butanol in D_2_O, focusing on the spectral region between 3.4 and 3.8 ppm. The plots were processed using **A)** a global phase correction and **B)** an individual phase correction. **C)** and **D)** show the phase variation (in degrees) across the 16 increments of the same DOSY experiment when **C)** a single global phase correction is applied to all increments simultaneously, and when **D)** manual phase correction is applied individually to each increment.
**Figure S35: A)** 400 MHz ^1^H‐DOSY plots and **B)** corresponding signal fitting plots for a mixture of ethanol, propanol, and butanol in D_2_O, focusing on the spectral region from 3.4 to 3.8 ppm, processed with a Gaussian window function of 1 Hz combined with manual and global phasing across all increments.
**Figure S36: A)** 400 MHz ^1^H‐DOSY plots and **B)** corresponding signal fitting plots for a mixture of ethanol, propanol, and butanol in D_2_O, focusing on the spectral region from 3.4 to 3.8 ppm, processed with a Gaussian window function of 1 Hz combined with manual and individual phasing for each increment.
**Figure S37: A)** 400 MHz ^1^H‐DOSY plots and **B)** corresponding signal fitting plots for a mixture of ethanol, propanol, and butanol in D_2_O, focusing on the spectral region from 3.4 to 3.8 ppm, processed with a Gaussian window function of 1 Hz. In all cases, manual phasing was performed for each increment, followed by automatic baseline correction (order 0).
**Figure S38: A)** 400 MHz ^1^H‐DOSY plots and **B)** corresponding signal fitting plots for a mixture of ethanol, propanol, and butanol in D_2_O, focusing on the spectral region from 3.4 to 3.8 ppm, processed with a Gaussian window function of 1 Hz. In all cases, manual phasing was performed for each increment, followed by automatic baseline correction (order 1).
**Figure S39: A)** 400 MHz ^1^H‐DOSY plots and **B)** corresponding signal fitting plots for a mixture of ethanol, propanol, and butanol in D_2_O, focusing on the spectral region from 3.4 to 3.8 ppm, processed with a Gaussian window function of 1 Hz. In all cases, manual phasing was performed for each increment, followed by automatic baseline correction (order 3).
**Figure S40: A)** 400 MHz ^1^H‐DOSY plots and **B)** corresponding signal fitting plots for a mixture of ethanol, propanol, and butanol in D_2_O, focusing on the spectral region from 3.4 to 3.8 ppm, processed with a Gaussian window function of 1 Hz. In all cases, manual phasing was performed for each increment, followed by automatic baseline correction (order 5).
**Figure S41: A)** 400 MHz ^1^H‐DOSY plots and **B)** corresponding signal fitting plots for a mixture of ethanol, propanol, and butanol in D_2_O, focusing on the spectral region from 3.4 to 3.8 ppm, processed with a Gaussian window function of 1 Hz. In all cases, manual phasing was performed for each increment, followed by automatic baseline correction (order 8).
**Figure S42: A)** 400 MHz ^1^H‐DOSY plots and **B)** corresponding signal fitting plots for a mixture of ethanol, propanol, and butanol in D_2_O, focusing on the spectral region from 3.4 to 3.8 ppm, processed with a Gaussian window function of 1 Hz. In all cases, manual phasing was performed for each increment, followed by manual baseline correction (order 0).
**Figure S43: A)** 400 MHz ^1^H‐DOSY plots and **B)** corresponding signal fitting plots for a mixture of ethanol, propanol, and butanol in D_2_O, focusing on the spectral region from 3.4 to 3.8 ppm, processed with a Gaussian window function of 1 Hz. In all cases, manual phasing was performed for each increment, followed by manual baseline correction (order 1).
**Figure S44: A)** 400 MHz ^1^H‐DOSY plots and **B)** corresponding signal fitting plots for a mixture of ethanol, propanol, and butanol in D_2_O, focusing on the spectral region from 3.4 to 3.8 ppm, processed with a Gaussian window function of 1 Hz. In all cases, manual phasing was performed for each increment, followed by manual baseline correction (order 3).
**Figure S45: A)** 400 MHz ^1^H‐DOSY plots and **B)** corresponding signal fitting plots for a mixture of ethanol, propanol, and butanol in D_2_O, focusing on the spectral region from 3.4 to 3.8 ppm, processed with a Gaussian window function of 1 Hz. In all cases, manual phasing was performed for each increment, followed by manual baseline correction (order 5).
**Figure S46: A)** 400 MHz ^1^H‐DOSY plots and **B)** corresponding signal fitting plots for a mixture of ethanol, propanol, and butanol in D_2_O, focusing on the spectral region from 3.4 to 3.8 ppm, processed with a Gaussian window function of 1 Hz. In all cases, manual phasing was performed for each increment, followed by manual baseline correction (order 8).
**Figure S47: A)** Diffusion coefficients of ethanol, propanol, butanol, and TSP in their mixture in deuterated water, along with their associated errors across ten different processing methods. **B)** The ^trends in the relative diffusion differences (^Δ^*D*^ethanol, propanol, butanol ^= *D*^ethanol, propanol, butanol ^*− D*^TSP^) and^ the corresponding propagated errors between ethanol and TSP, propanol and TSP, and butanol and TSP across the different processing methods.
**Figure S48:** mrc70110‐sup‐0001‐Supporting_Information.docx. ^1^H NMR (400 MHz) signal of TSP **A)** before and **B)** after the application of Reference Deconvolution using a Lorentzian window function (LW) of 1 Hz.
**Table S1:** mrc70110‐sup‐0001‐Supporting_Information.docx. ^1^H‐DOSY (400 MHz) data for a mixture of propanol, butanol, and ethanol in D_2_O, processed without any window function. Chemical shifts correspond to the region from 3.4 to 3.8 ppm.
**Table S2:** mrc70110‐sup‐0001‐Supporting_Information.docx. ^1^H‐DOSY (400 MHz) data for a mixture of propanol, butanol, and ethanol in D_2_O, processed with a Lorentzian window function of 1 Hz. Chemical shifts correspond to the region from 3.4 to 3.8 ppm.
**Table S3:** mrc70110‐sup‐0001‐Supporting_Information.docx. ^1^H‐DOSY (400 MHz) data for a mixture of propanol, butanol, and ethanol in D_2_O, processed with a Lorentzian window function of 2 Hz. Chemical shifts correspond to the region from 3.4 to 3.8 ppm.
**Table S4:** mrc70110‐sup‐0001‐Supporting_Information.docx. ^1^H‐DOSY (400 MHz) data for a mixture of propanol, butanol, and ethanol in D_2_O, processed with a Gaussian window function of 1 Hz. Chemical shifts correspond to the region from 3.4 to 3.8 ppm.
**Table S5:** mrc70110‐sup‐0001‐Supporting_Information.docx. ^1^H‐DOSY (400 MHz) data for a mixture of propanol, butanol, and ethanol in D_2_O, processed with a Gaussian window function of 2 Hz. Chemical shifts correspond to the region from 3.4 to 3.8 ppm.
**Table S6:** mrc70110‐sup‐0001‐Supporting_Information.docx. ^1^H‐DOSY (400 MHz) data for a mixture of propanol, butanol, and ethanol in D_2_O, processed with a combined Lorentzian and Gaussian window functions of 1 Hz. Chemical shifts correspond to the region from 3.4 to 3.8 ppm.
**Table S7:** mrc70110‐sup‐0001‐Supporting_Information.docx. ^1^H‐DOSY (400 MHz) data for a mixture of propanol, butanol, and ethanol in D_2_O, processed with reference deconvolution based on a Lorentzian window function of 1 Hz. Chemical shifts correspond to the region from 3.4 to 3.8 ppm.
**Table S8:** mrc70110‐sup‐0001‐Supporting_Information.docx. ^1^H‐DOSY (400 MHz) data for a mixture of propanol, butanol, and ethanol in D_2_O, processed with reference deconvolution based on a Gaussian window function of 1 Hz. Chemical shifts correspond to the region from 3.4 to 3.8 ppm.
**Table S9:** mrc70110‐sup‐0001‐Supporting_Information.docx. ^1^H‐DOSY (400 MHz) data for a mixture of propanol, butanol, and ethanol in D_2_O, processed with reference deconvolution based on a Lorentzian window function −0.3 Hz and a Gaussian window function of 1 Hz. Chemical shifts correspond to the region from 3.4 to 3.8 ppm.
**Table S10:** mrc70110‐sup‐0001‐Supporting_Information.docx. ^1^H‐DOSY (400 MHz) data for a mixture of propanol, butanol, and ethanol in D_2_O, processed with reference deconvolution based on a combined Lorentzian window function and Gaussian window function of 1 Hz. Chemical shifts correspond to the region from 3.4 to 3.8 ppm.
**Table S11:** mrc70110‐sup‐0001‐Supporting_Information.docx. ^1^H‐DOSY (400 MHz) data for a mixture of propanol, butanol, and ethanol in D_2_O, processed with a Lorentzian window function of 0.5 Hz. Chemical shifts correspond to the region from 3.4 to 3.8 ppm.
**Table S12:** mrc70110‐sup‐0001‐Supporting_Information.docx. ^1^H‐DOSY (400 MHz) data for a mixture of propanol, butanol, and ethanol in D_2_O, processed with a Gaussian window function of 0.5 Hz. Chemical shifts correspond to the region from 3.4 to 3.8 ppm.
**Table S13:** mrc70110‐sup‐0001‐Supporting_Information.docx. ^1^H‐DOSY (400 MHz) data for a mixture of propanol, butanol, and ethanol in D_2_O, processed with a combined Lorentzian window function of −0.5 Hz and Gaussian width of 0.5 Hz. Chemical shifts correspond to the region from 3.4 to 3.8 ppm.
**Table S14:** mrc70110‐sup‐0001‐Supporting_Information.docx. ^1^H‐DOSY (400 MHz) data for a mixture of propanol, butanol, and ethanol in D_2_O, processed with a combined Lorentzian window function of 0.5 Hz and Gaussian width of 0.5 Hz. Chemical shifts correspond to the region from 3.4 to 3.8 ppm.
**Table S15:** mrc70110‐sup‐0001‐Supporting_Information.docx. ^1^H‐DOSY (400 MHz) data for a mixture of propanol, butanol, and ethanol in D_2_O, processed using reference deconvolution based on a Lorentzian window function of 0.5 Hz. Chemical shifts correspond to the region from 3.4 to 3.8 ppm.
**Table S16:** mrc70110‐sup‐0001‐Supporting_Information.docx. ^1^H‐DOSY (400 MHz) data for a mixture of propanol, butanol, and ethanol in D_2_O, processed using reference deconvolution based on a Gaussian window function of 0.5 Hz. Chemical shifts correspond to the region from 3.4 to 3.8 ppm.
**Table S17:** mrc70110‐sup‐0001‐Supporting_Information.docx. ^1^H‐DOSY (400 MHz) data for a mixture of propanol, butanol, and ethanol in D_2_O, processed using reference deconvolution based on a combined Lorentzian window function of −0.5 Hz and Gaussian width of 0.5 Hz. Chemical shifts correspond to the region from 3.4 to 3.8 ppm.
**Table S18:** mrc70110‐sup‐0001‐Supporting_Information.docx. ^1^H‐DOSY (400 MHz) data for a mixture of propanol, butanol, and ethanol in D_2_O, processed using reference deconvolution based on a combined Lorentzian window function of 0.5 Hz and Gaussian width of 0.5 Hz. Chemical shifts correspond to the region from 3.4 to 3.8 ppm.
**Table S19:** mrc70110‐sup‐0001‐Supporting_Information.docx. ^1^H‐DOSY (400 MHz) data for a mixture of propanol, butanol, and ethanol in D_2_O, processed with a Gaussian window function of 1 Hz combined with manual and global phasing across all increments. Chemical shifts correspond to the region from 3.4 to 3.8 ppm.
**Table S20:** mrc70110‐sup‐0001‐Supporting_Information.docx. ^1^H‐DOSY (400 MHz) data for a mixture of propanol, butanol, and ethanol in D_2_O, processed with a Gaussian window function of 1 Hz combined with manual and individual phasing for each increment. Chemical shifts correspond to the region from 3.4 to 3.8 ppm.
**Table S21:** mrc70110‐sup‐0001‐Supporting_Information.docx. ^1^H‐DOSY (400 MHz) data for a mixture of propanol, butanol, and ethanol in D_2_O, processed with a Gaussian window function of 1 Hz, followed by automatic baseline correction (order 0). Chemical shifts correspond to the region from 3.4 to 3.8 ppm.
**Table S22:** mrc70110‐sup‐0001‐Supporting_Information.docx. ^1^H‐DOSY (400 MHz) data for a mixture of propanol, butanol, and ethanol in D_2_O, processed with a Gaussian window function of 1 Hz, followed by automatic baseline correction (order 1). Chemical shifts correspond to the region from 3.4 to 3.8 ppm.
**Table S23:** mrc70110‐sup‐0001‐Supporting_Information.docx. ^1^H‐DOSY (400 MHz) data for a mixture of propanol, butanol, and ethanol in D_2_O, processed with a Gaussian window function of 1 Hz, followed by automatic baseline correction (order 3). Chemical shifts correspond to the region from 3.4 to 3.8 ppm.
**Table S24:** mrc70110‐sup‐0001‐Supporting_Information.docx. ^1^H‐DOSY (400 MHz) data for a mixture of propanol, butanol, and ethanol in D_2_O, processed with a Gaussian window function of 1 Hz, followed by automatic baseline correction (order 5). Chemical shifts correspond to the region from 3.4 to 3.8 ppm.
**Table S25:** mrc70110‐sup‐0001‐Supporting_Information.docx. ^1^H‐DOSY (400 MHz) data for a mixture of propanol, butanol, and ethanol in D_2_O, processed with a Gaussian window function of 1 Hz, followed by automatic baseline correction (order 8). Chemical shifts correspond to the region from 3.4 to 3.8 ppm.
**Table S26:** mrc70110‐sup‐0001‐Supporting_Information.docx. ^1^H‐DOSY (400 MHz) data for a mixture of propanol, butanol, and ethanol in D_2_O, processed with a Gaussian window function of 1 Hz, followed by manual baseline correction (order 0). Chemical shifts correspond to the region from 3.4 to 3.8 ppm.
**Table S27:** mrc70110‐sup‐0001‐Supporting_Information.docx. ^1^H‐DOSY (400 MHz) data for a mixture of propanol, butanol, and ethanol in D_2_O, processed with a Gaussian window function of 1 Hz, followed by manual baseline correction (order 1). Chemical shifts correspond to the region from 3.4 to 3.8 ppm.
**Table S28:** mrc70110‐sup‐0001‐Supporting_Information.docx. ^1^H‐DOSY (400 MHz) data for a mixture of propanol, butanol, and ethanol in D_2_O, processed with a Gaussian window function of 1 Hz, followed by manual baseline correction (order 3). Chemical shifts correspond to the region from 3.4 to 3.8 ppm.
**Table S29:** mrc70110‐sup‐0001‐Supporting_Information.docx. ^1^H‐DOSY (400 MHz) data for a mixture of propanol, butanol, and ethanol in D_2_O, processed with a Gaussian window function of 1 Hz, followed by manual baseline correction (order 5). Chemical shifts correspond to the region from 3.4 to 3.8 ppm.
**Table S30:** mrc70110‐sup‐0001‐Supporting_Information.docx. ^1^H‐DOSY (400 MHz) data for a mixture of propanol, butanol, and ethanol in D_2_O, processed with a Gaussian window function of 1 Hz, followed by manual baseline correction (order 8). Chemical shifts correspond to the region from 3.4 to 3.8 ppm.
**Table S31:** Signal‐to‐noise ratios (SNRs) of selected frequencies for propanol, butanol, ethanol, and TSP under various processing methods. Values were extracted from signals in the region of 3.4–3.8 ppm: approximately 3.538 ppm (propanol), 3.587 ppm (butanol), 3.678 ppm (ethanol), and 0.00 ppm (TSP).
**Table S32:** Signal‐to‐noise ratios (SNRs) of selected frequencies for propanol, butanol, ethanol, and TSP under various processing methods. Values were extracted from signals in the region of 3.4–3.8 ppm: approximately 3.538 ppm (propanol), 3.587 ppm (butanol), 3.678 ppm (ethanol), and 0.00 ppm (TSP). All processing parameters were selected based on the experimental line‐width at half height of the TSP signal (0.5 Hz).
**Table S33:** Average diffusion coefficients and their corresponding propagated errors (both expressed in *×*10^*−*10^ m^2^ s^−1^) for butanol, propanol, ethanol, and TSP under various processing conditions. The signal fitting was performed in the spectral region from 3.4 to 3.8 ppm for the alcohols and at 0.0 ppm for TSP.

## Data Availability

The data that support the findings of this study are openly available in REDU at https://doi.org/10.25824/redu/LTE2ZC.
