# Supplementary material for: Practical Guide and Best Practices for Diffusion NMR Processing With GNAT
Source: Magn Reson Chem. 2026 May 10;64(8):729–44. doi: 10.1002/mrc.70110 (PMC13327220; doi:10.1002/mrc.70110)
Supplement: Supplementary file 1 — Figure S1: Importing diffusion‐NMR data into GNAT. Figure S2: Application of a Fourier transformation. Figure S3: Automatic phase correction. Figure S4: Manual global phase correction. Figure S5: Individual phase correction across diffusion increments. Figure S6: Manual baseline correction. Figure S7: Selection of reference signal region. Figure S8: Chemical shift referencing. Figure S9: Reference deconvolution workflow. Figure S10: Generation of DOSY plot. Figure S11: DOSY visualization interface. Figure S12: Exporting DOSY plots. Figure S13: Terminal displaying the diffusion coefficients and fitting statistics. Figure S14: Access to further information on the fitting results. Figure S15: Fits and residuals for selected peaks. Figure S16: A) 400 MHz 1H‐DOSY plots and B) corresponding signal fitting plots for a mixture of ethanol, propanol, and butanol in D2O, focusing on the spectral region from 3.4 to 3.8 ppm, processed without any window function. In all cases, manual phasing for each increment and manual baseline correction were applied. Figure S17: A) 400 MHz 1H‐DOSY plots and B) corresponding signal fitting plots for a mixture of ethanol, propanol, and butanol in D2O, focusing on the spectral region from 3.4 to 3.8 ppm, processed with a Lorentzian window function of 1 Hz. In all cases, manual phasing for each increment and manual baseline correction were applied. Figure S18: A) 400 MHz 1H‐DOSY plots and B) corresponding signal fitting plots for a mixture of ethanol, propanol, and butanol in D2O, focusing on the spectral region from 3.4 to 3.8 ppm, processed with a Lorentzian window function of 2 Hz. In all cases, manual phasing for each increment and manual baseline correction were applied. Figure S19: A) 400 MHz 1H‐DOSY plots and B) corresponding signal fitting plots for a mixture of ethanol, propanol, and butanol in D2O, focusing on the spectral region from 3.4 to 3.8 ppm, processed with a Gaussian window function of 1 Hz. In all cases, manual phasing for ea [file MRC-64-729-s001.docx]

Supporting Information

Practical Guide and Best Practices for Diffusion NMR Processing with GNAT

Tadeu Luiz Gomes Cabral*^a^*, Guilherme Dal Poggetto*^b^*, Claudio F Tormena*^a,∗^*,

Mathias Nilsson*^c,∗^*

*^a^*Institute of Chemistry, Physical Organic Chemistry Lab, University of Campinas - UNICAMP, Campinas - São Paulo, Brazil.

*^b^*Merck & Co. Inc., Analytical Research & Development, 126 Lincoln Ave, Rahway, NJ, USA.

*^c^*Department of Chemistry, University of Manchester, Oxford Road, Manchester M13 9PL, UK.

**Emails:**

[t219502@dac.unicamp.br](mailto:t219502@dac.unicamp.br) [guilherme.dal.poggetto@merck.com](mailto:guilherme.dal.poggetto@merck.com) [tormena@unicamp.br](mailto:tormena@unicamp.br) [mathias.nilsson@manchester.ac.uk](mailto:mathias.nilsson@manchester.ac.uk)

# Contents

| [**1 Summary of Processing Steps**](#_bookmark0) | **S3** |
| --- | --- |
| [**2 Checklist for Proper Diffusion Processing**](#_bookmark1) | **S4** |
| [**3 Step-by-Step Processing Images**](#_bookmark2) | **S4** |
| [**4 Further Information on DOSY Processing**](#_bookmark3) | **S12** |
| [4.1 Processing Without Window Functions](#_bookmark4) . . . . . . . . . . . . . . . . . . . . . . . . . | S13 |
| [4.2 Processing with a Lorentzian Window Function (1 Hz)](#_bookmark5) . . . . . . . . . . . . . . . . | S14 |
| [4.3 Processing with a Lorentzian Window Function (2 Hz)](#_bookmark6) . . . . . . . . . . . . . . . . | S15 |
| [4.4 Processing with a Gaussian Window Function (1 Hz)](#_bookmark7) . . . . . . . . . . . . . . . . . | S16 |
| [4.5 Processing with a Gaussian Window Function (2 Hz)](#_bookmark8) . . . . . . . . . . . . . . . . . | S17 |
| [4.6 Processing with a Combined Lorentzian and Gaussian Window Functions (1 Hz)](#_bookmark9) . . | S18 |
| [4.7 Reference Deconvolution Based on a Lorentzian Window Function (1 Hz)](#_bookmark10) . . . . . . | S19 |
| [4.8 Reference Deconvolution Based on a Gaussian Window Function (1 Hz)](#_bookmark11) . . . . . . . | S20 |
| [4.9 Reference Deconvolution Based on a Combined Lorentzian Window Function (-0.3](#_bookmark12) |  |
| [Hz) and Gaussian (1 Hz)](#_bookmark12) . . . . . . . . . . . . . . . . . . . . . . . . . . . . . . . . | S21 |
| [4.10 Reference Deconvolution Based on a Combined Lorentzian and Gaussian Window](#_bookmark13) |  |
| [Functions (1 Hz)](#_bookmark13) . . . . . . . . . . . . . . . . . . . . . . . . . . . . . . . . . . . . | S22 |
| [4.11 Processing with a Lorentzian Window Function (0.5 Hz)](#_bookmark14) . . . . . . . . . . . . . . . | S23 |
| [4.12 Processing with a Gaussian Window Function (0.5 Hz)](#_bookmark15) . . . . . . . . . . . . . . . . | S24 |
| [4.13 Processing with a Combined Lorentzian Window Function (-0.5 Hz) and Gaussian](#_bookmark16) |  |
| [(0.5 Hz)](#_bookmark16) . . . . . . . . . . . . . . . . . . . . . . . . . . . . . . . . . . . . . . . . . | S25 |
| [4.14 Processing with a Combined Lorentzian Window Function (0.5 Hz) and Gaussian (0.5](#_bookmark17) |  |
| [Hz)](#_bookmark17) . . . . . . . . . . . . . . . . . . . . . . . . . . . . . . . . . . . . . . . . . . . | S26 |
| [4.15 Reference Deconvolution Based on a Lorentzian Window Function (0.5 Hz)](#_bookmark18) . . . . . | S27 |
| [4.16 Reference Deconvolution Based on a Gaussian Window Function (0.5 Hz)](#_bookmark19) . . . . . . | S28 |
| [4.17 Reference Deconvolution Based on a Combined Lorentzian Window Function (-0.5](#_bookmark20) |  |
| [Hz) and Gaussian (0.5 Hz)](#_bookmark20) . . . . . . . . . . . . . . . . . . . . . . . . . . . . . . . | S29 |
| [4.18 Reference Deconvolution Based on a Combined Lorentzian Window Function (0.5](#_bookmark21) |  |
| [Hz) and Gaussian (0.5 Hz)](#_bookmark21) . . . . . . . . . . . . . . . . . . . . . . . . . . . . . . . | S30 |
| [**5 Processing with a Gaussian Window (1 Hz) - Global and Individual Phases Series**](#_bookmark22) | **S31** |
| [5.1 Global Phase Correction](#_bookmark23) . . . . . . . . . . . . . . . . . . . . . . . . . . . . . . . . | S32 |
| [5.2 Individual Phase Correction](#_bookmark24) . . . . . . . . . . . . . . . . . . . . . . . . . . . . . . . | S33 |
| [**6 Processing with a Gaussian Window (1 Hz) - Baseline Order Series**](#_bookmark25) | **S34** |
| [6.1 Automatic Baseline Correction (order 0)](#_bookmark26) . . . . . . . . . . . . . . . . . . . . . . . . | S34 |
| [6.2 Automatic Baseline Correction (order 1)](#_bookmark27) . . . . . . . . . . . . . . . . . . . . . . . . | S35 |
| [6.3 Automatic Baseline Correction (order 3)](#_bookmark28) . . . . . . . . . . . . . . . . . . . . . . . . | S36 |

- 1. [Automatic Baseline Correction (order 5)](#_bookmark29) S37
  2. [Automatic Baseline Correction (order 8)](#_bookmark30) S38

| [6.6 Manual Baseline Correction (order 0)](#_bookmark31) | . . . . . . . . . . . . . . . . . . . . . . . . . | S39 |
| --- | --- | --- |
| [6.7 Manual Baseline Correction (order 1)](#_bookmark32) | . . . . . . . . . . . . . . . . . . . . . . . . . | S40 |
| [6.8 Manual Baseline Correction (order 3)](#_bookmark33) | . . . . . . . . . . . . . . . . . . . . . . . . . | S41 |
| [6.9 Manual Baseline Correction (order 5)](#_bookmark34) | . . . . . . . . . . . . . . . . . . . . . . . . . | S42 |
| [6.10 Manual Baseline Correction (order 8)](#_bookmark35) | . . . . . . . . . . . . . . . . . . . . . . . . . | S43 |

1. [**Absolute Signal-to-Noise (SNR) Ratio Values**](#_bookmark36) **S44**
2. [**Average Diffusion Coefficients and Relative Differences**](#_bookmark37) **S45**
3. [**Effect of Reference Deconvolution on the TSP Signal**](#_bookmark38) **S50**

# Summary of Processing Steps

The step-by-step procedures described in the main text are summarized below to provide a quick reference guide for DOSY processing.

1. **Import Data**

File *→* Import *→* Bruker *→* Select Folder

1. **Fourier Transform**

FT panel *→* fn *→* LW/GW window function *→* FT

1. **Phase Correction**
   1. **Global Phase Correction:**

Phase panel *→* Auto *→* Scale *→* Set Pivot *→* Click on Spectrum *→* Adjust Zero/First Order

- 1. **Individual Phase Correction:**

Navigate to final spectrum *→* Individual *→* Manually adjust phase using Zero/First Order for each spectrum

1. **Baseline Correction**

Correct panel *→* Scale *→* Set *→* Order 5 *→* Click on Spectrum *→* Apply

1. **Chemical Shift Referencing**

Zoom into reference signal *→* FT panel *→* Set *→* Click on Spectrum *→* Ref *→* Enter reference value (e.g., 0.00 ppm) *→* OK

1. **Reference Deconvolution (Optional)**

Correct panel *→* Set Limits (Left/Right) *→* Click on Spectrum *→* Set Centre *→* Click on Spectrum *→* Select line shape, line broadening, and peak type *→* FIDDLE

1. **DOSY Calculation**

Rescale spectrum *→* Thresh *→* Click on Spectrum *→* Diffusion *→* DOSY *→* Select fitting settings *→* Run

1. **Visualization & Export**

Adjust plot via Zoom/Pan/Scale *→* Click on Separate/Publication Plot *→* Save or export

1. **Saving Diffusion Data**

Click Stats or check the terminal to view/export diffusion coefficients and statistical parameters

1. **Fitting Analysis**

Check the boxes and enter peak numbers *→* Enable Original/Fitted/Residual checkboxes *→*

Compare Fit *→* Save

# Checklist for Proper Diffusion Processing

To ensure reliable DOSY results, we strongly recommend verifying the following criteria as part of the processing workflow:

- All phase and baseline corrections have been properly applied.
- The DOSY plot displays consistent and well-resolved
- The residual errors from the fitting are minimal (as close to zero as possible) and constant across all diffusion increments.
- The errors in the estimated diffusion coefficients are as low as possible.
- The diffusion trends (i.e., relative differences between diffusion coefficients) are consistent across different processing methods.
- The signal-to-noise ratio (SNR) for the relevant peaks in the first diffusion increment is greater than 200 or as high as possible.
- When applicable, an average diffusion coefficient should be reported for each compound, along with its propagated error based on multiple signals of the same molecule.

# Step-by-Step Processing Images

Here, higher-resolution, double-column versions of Figures 2–16 presented in the main text are provided.


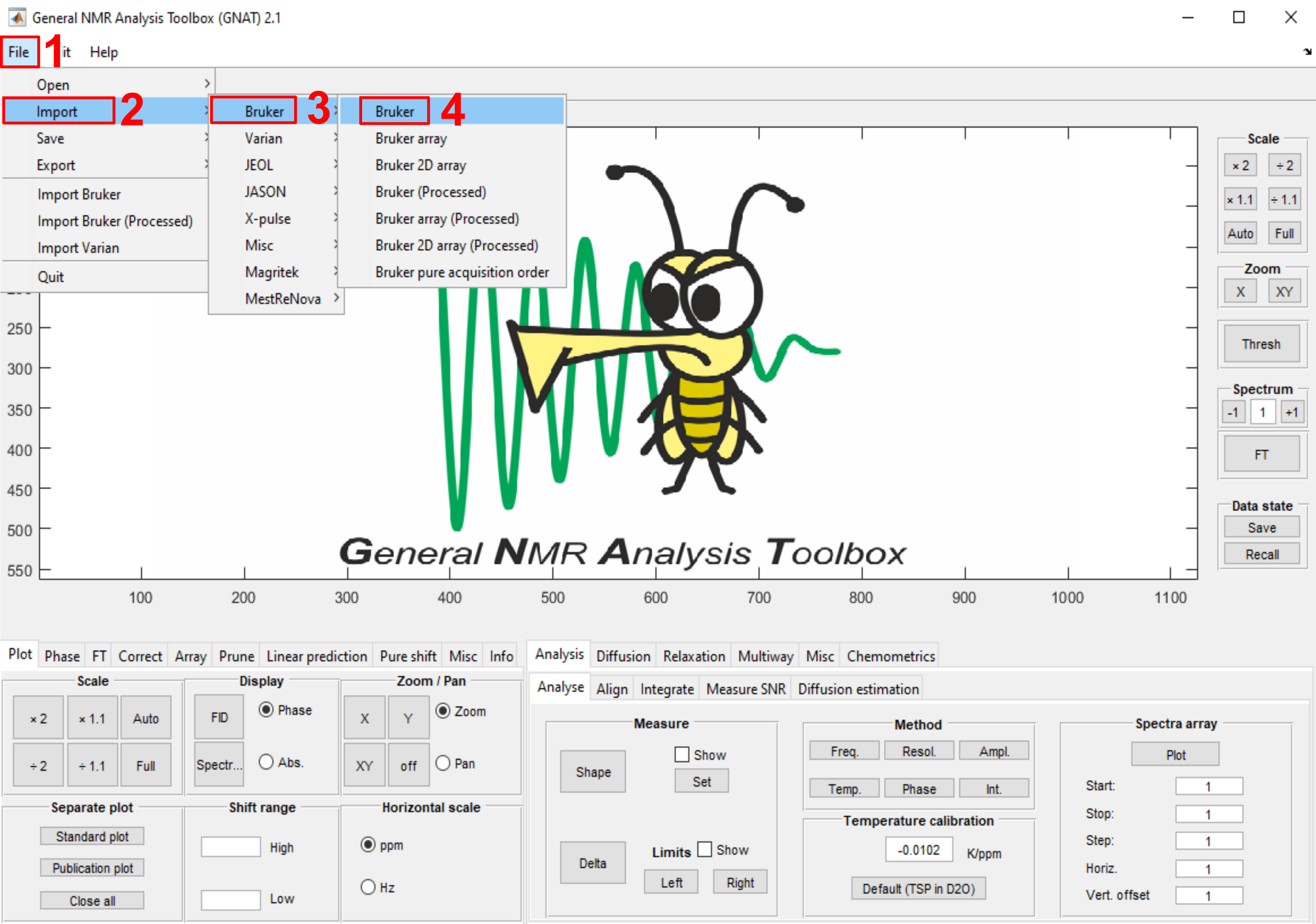


**Figure S1:** Importing diffusion-NMR data into GNAT.


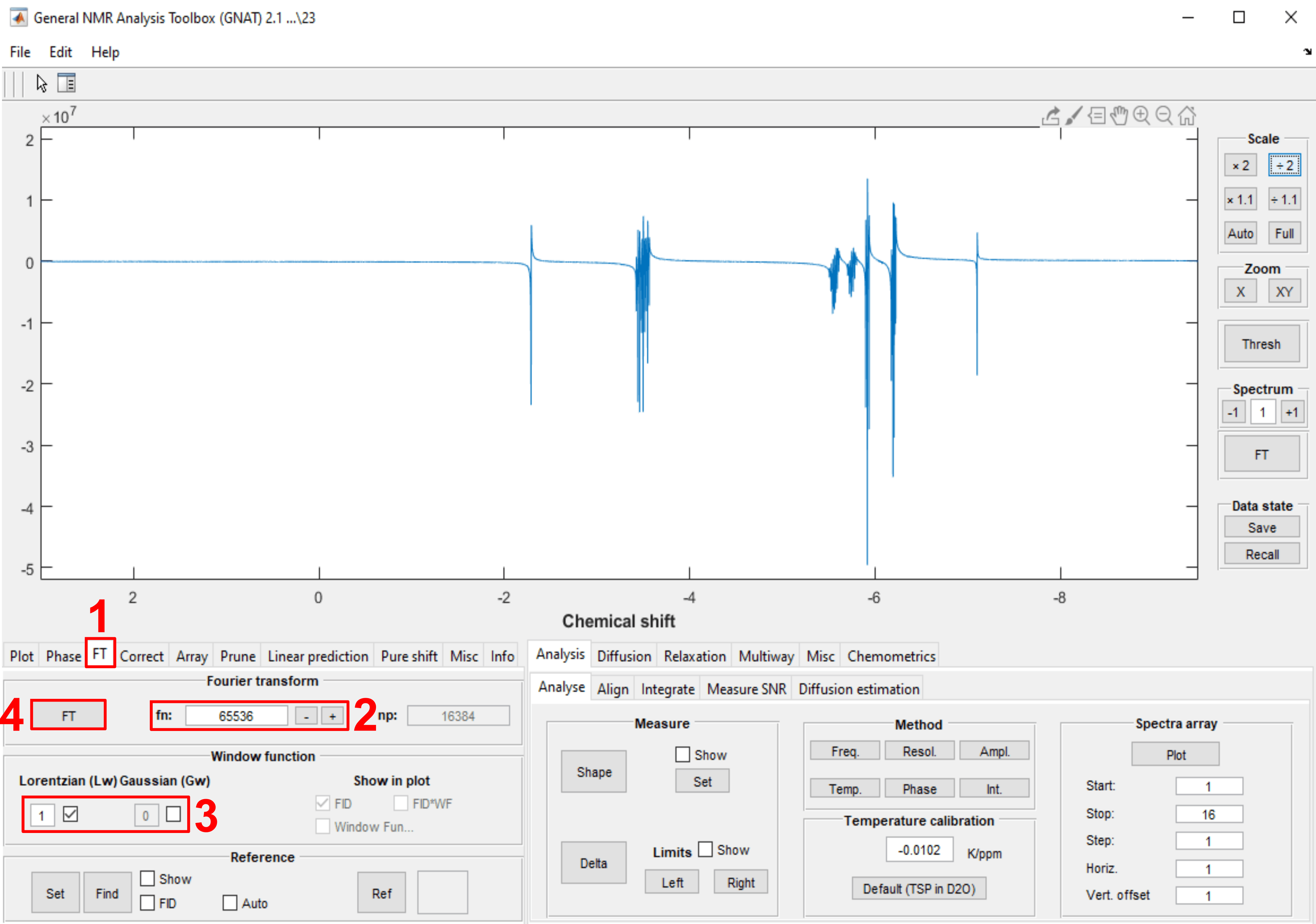


**Figure S2:** Application of a Fourier transformation


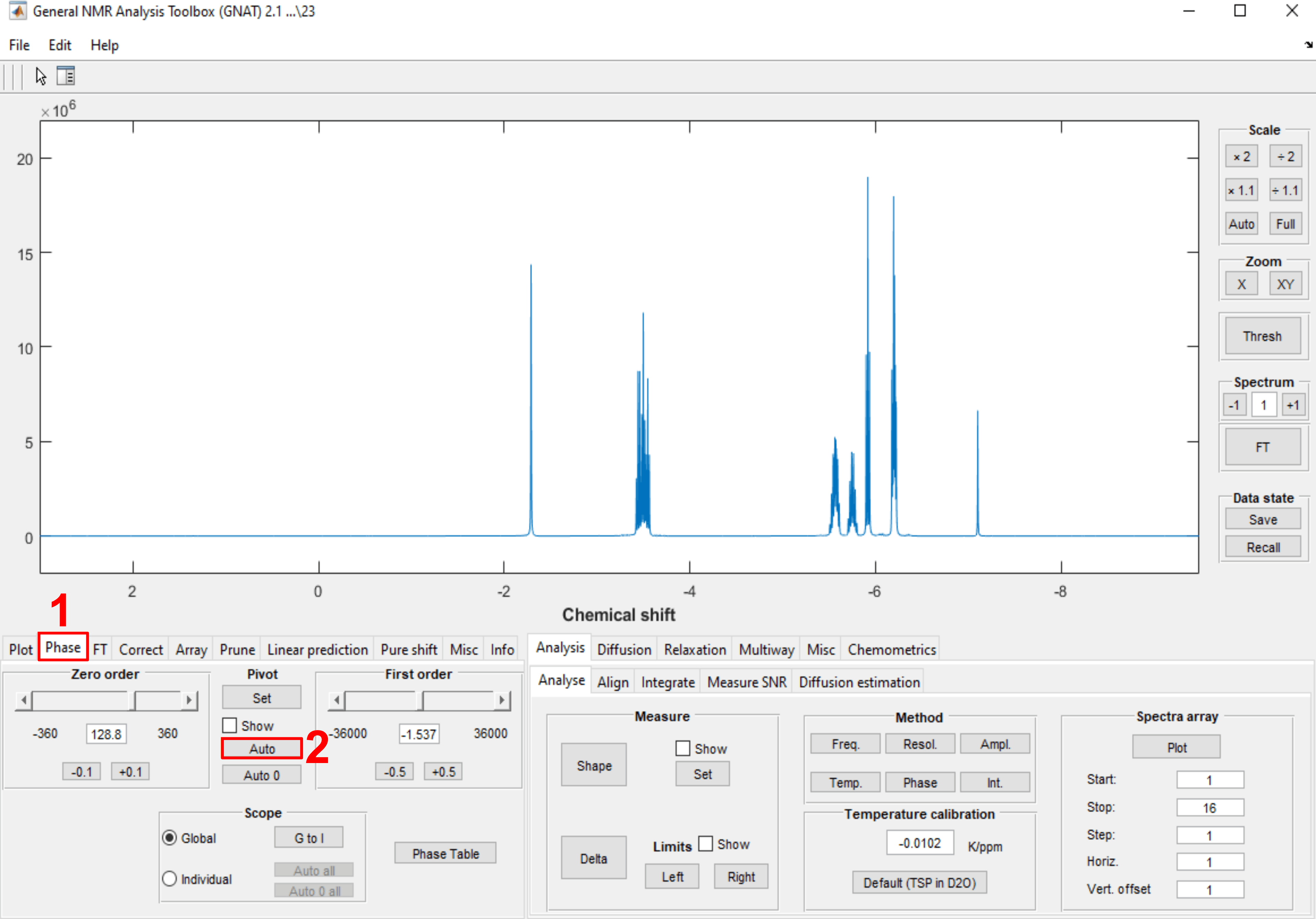


**Figure S3:** Automatic phase correction.


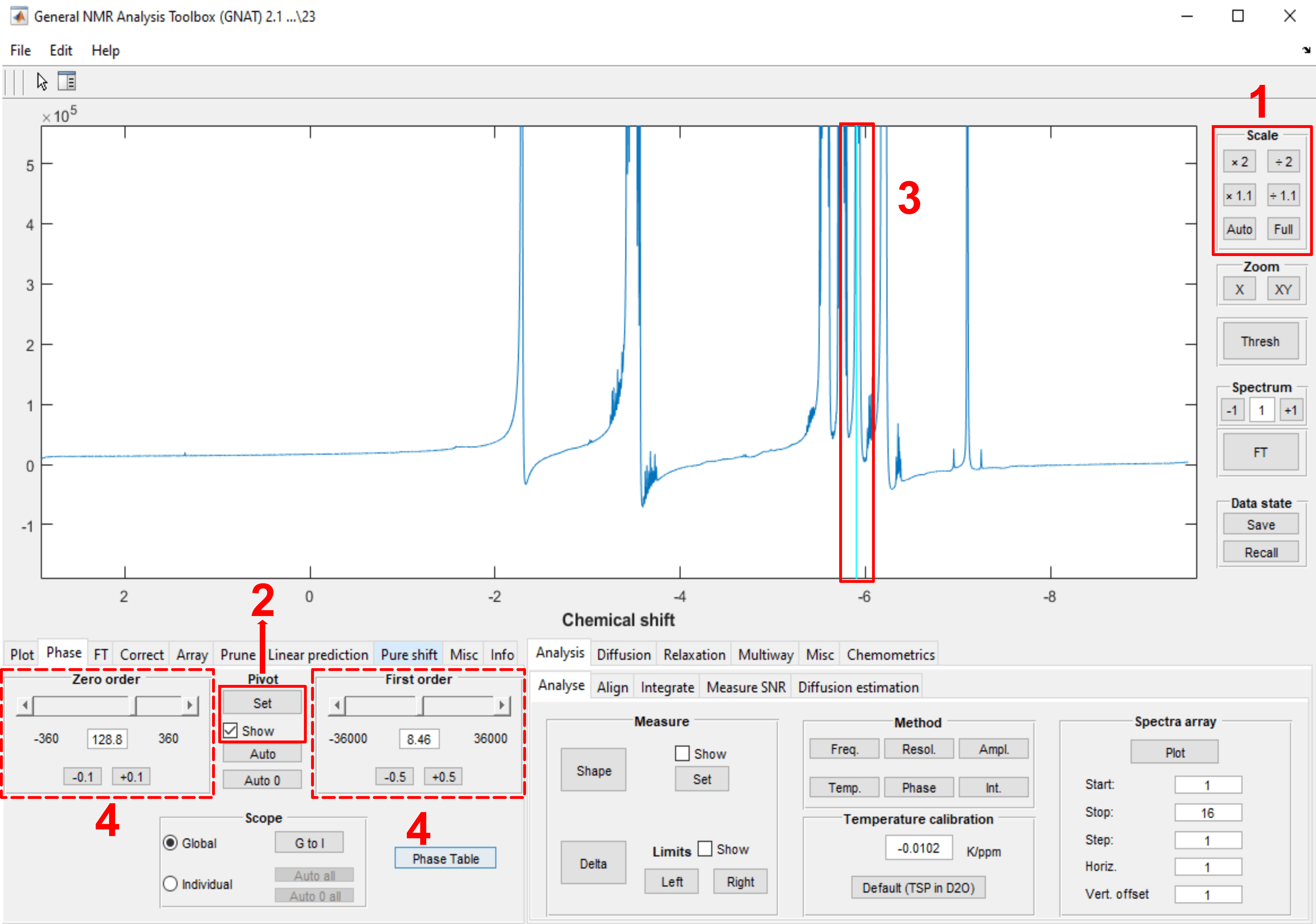


**Figure S4:** Manual global phase correction.


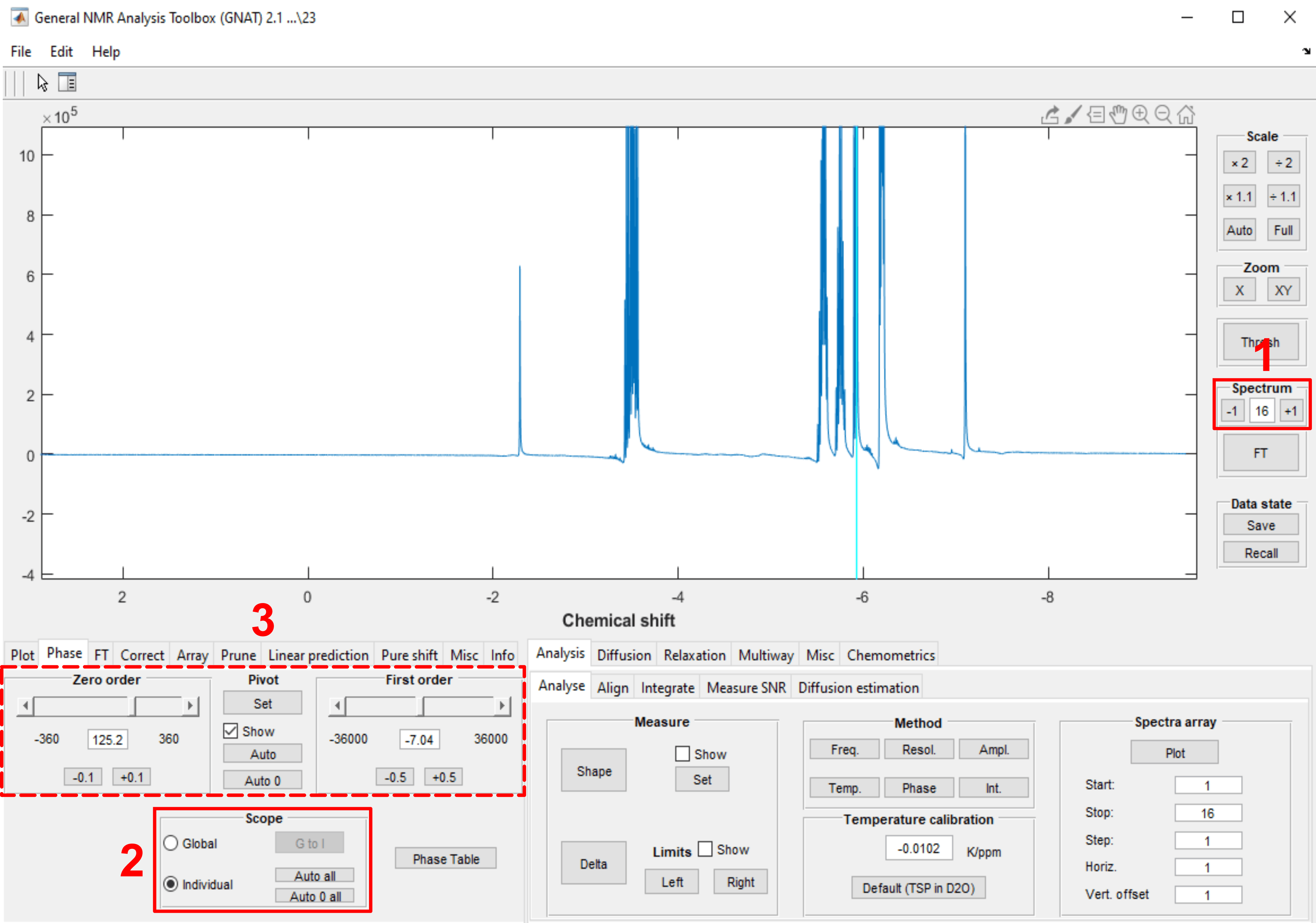


**Figure S5:** Individual phase correction across diffusion increments.


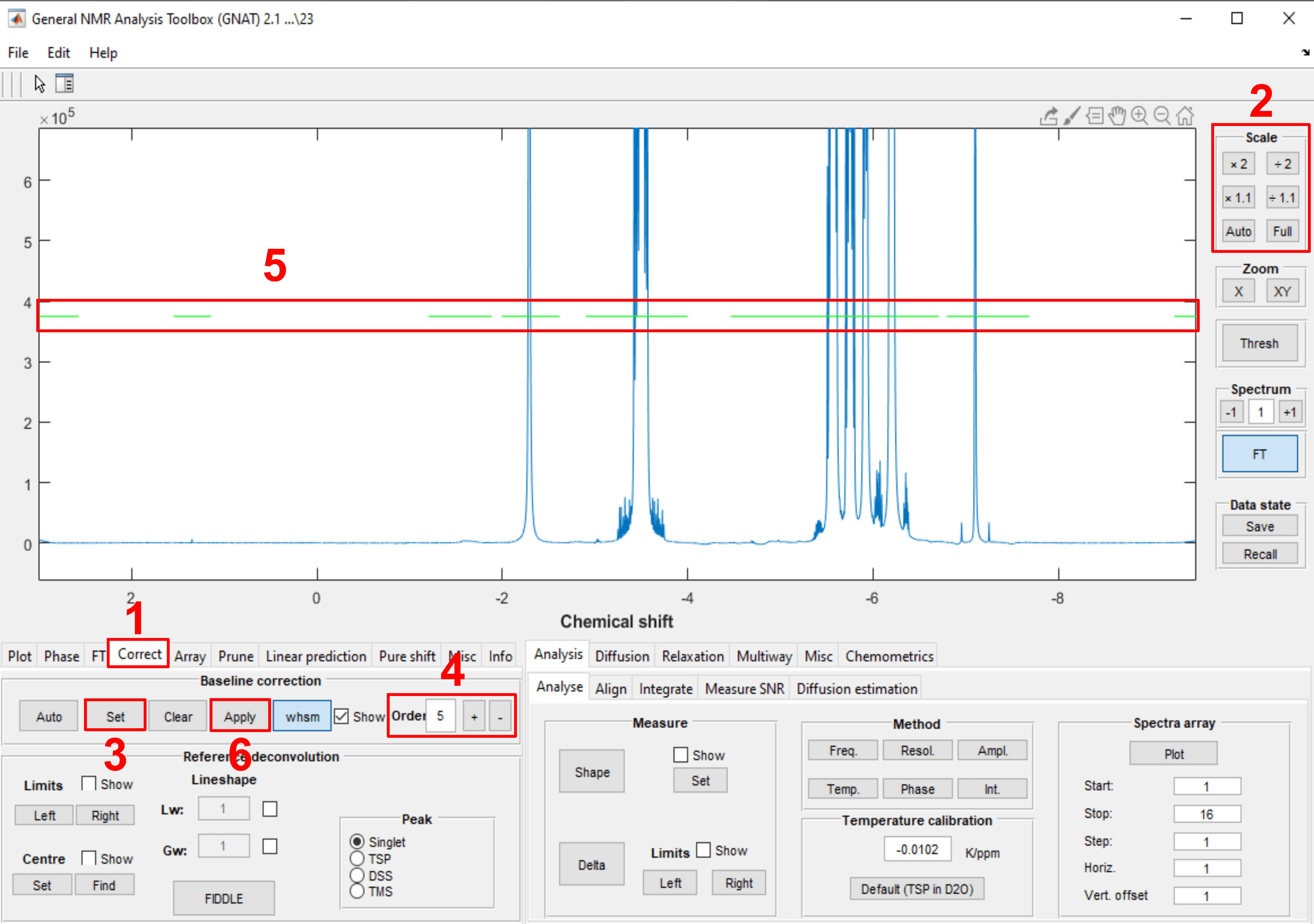


**Figure S6:** Manual baseline correction.


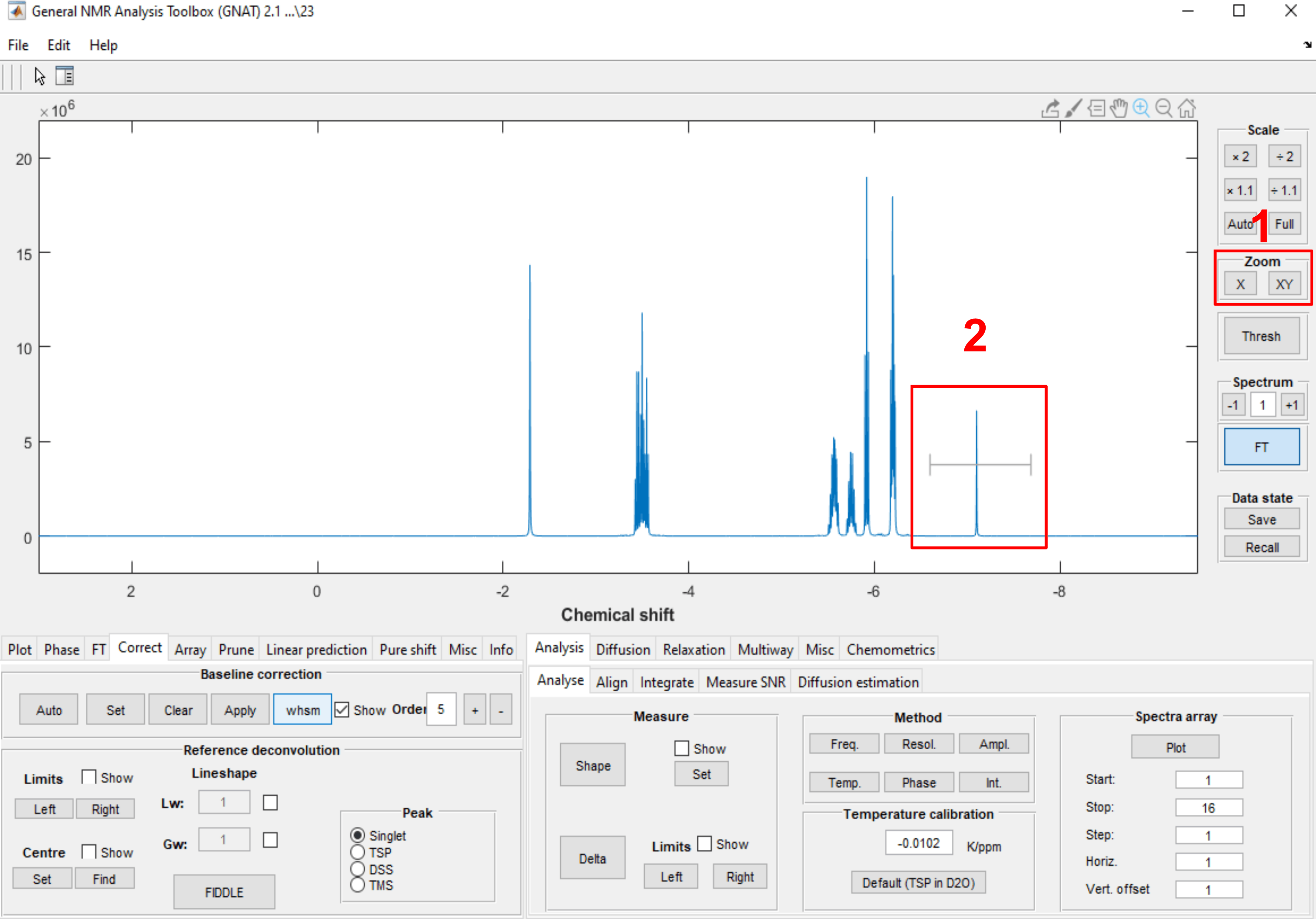


**Figure S7:** Selection of reference signal region.


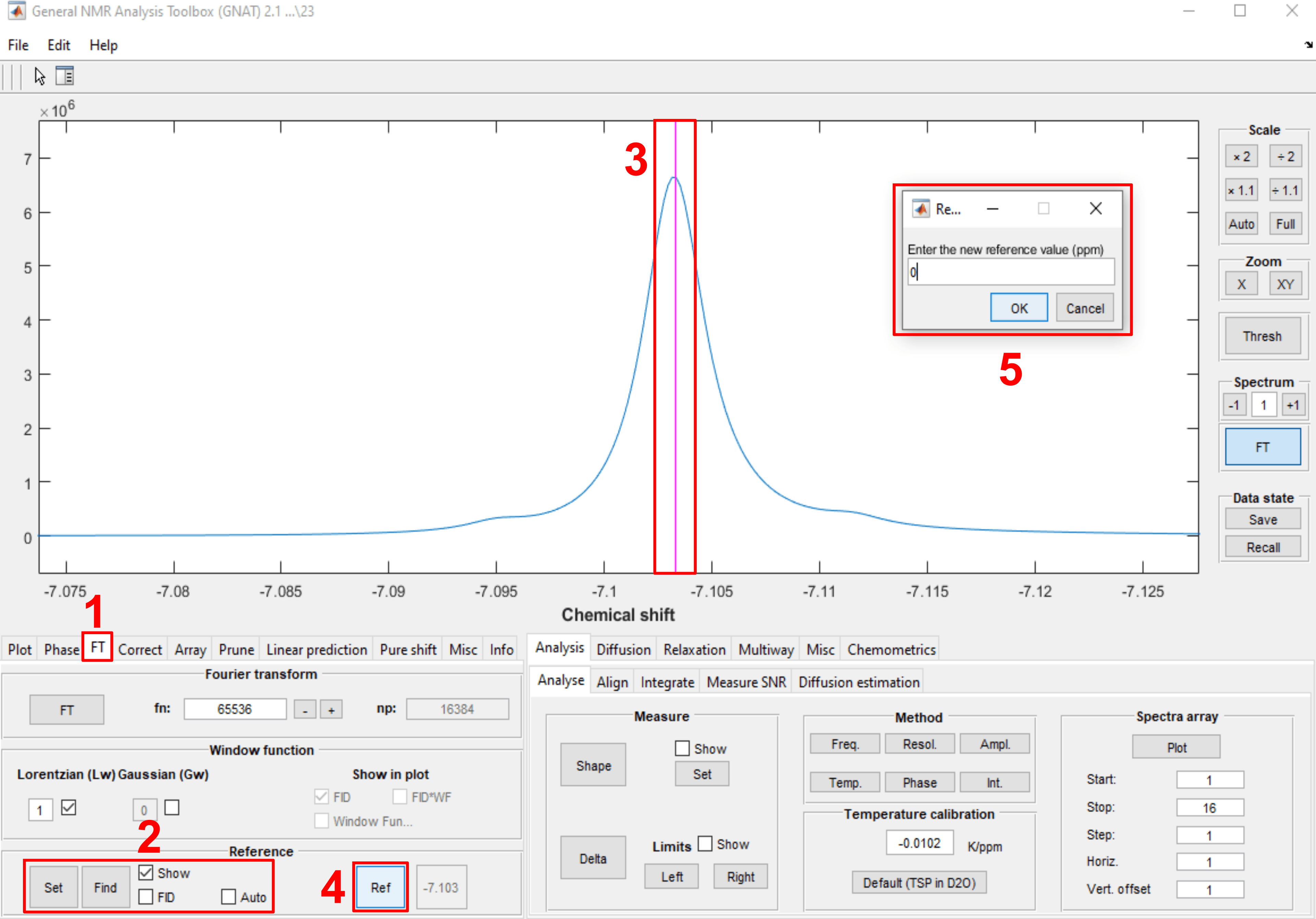


**Figure S8:** Chemical shift referencing.


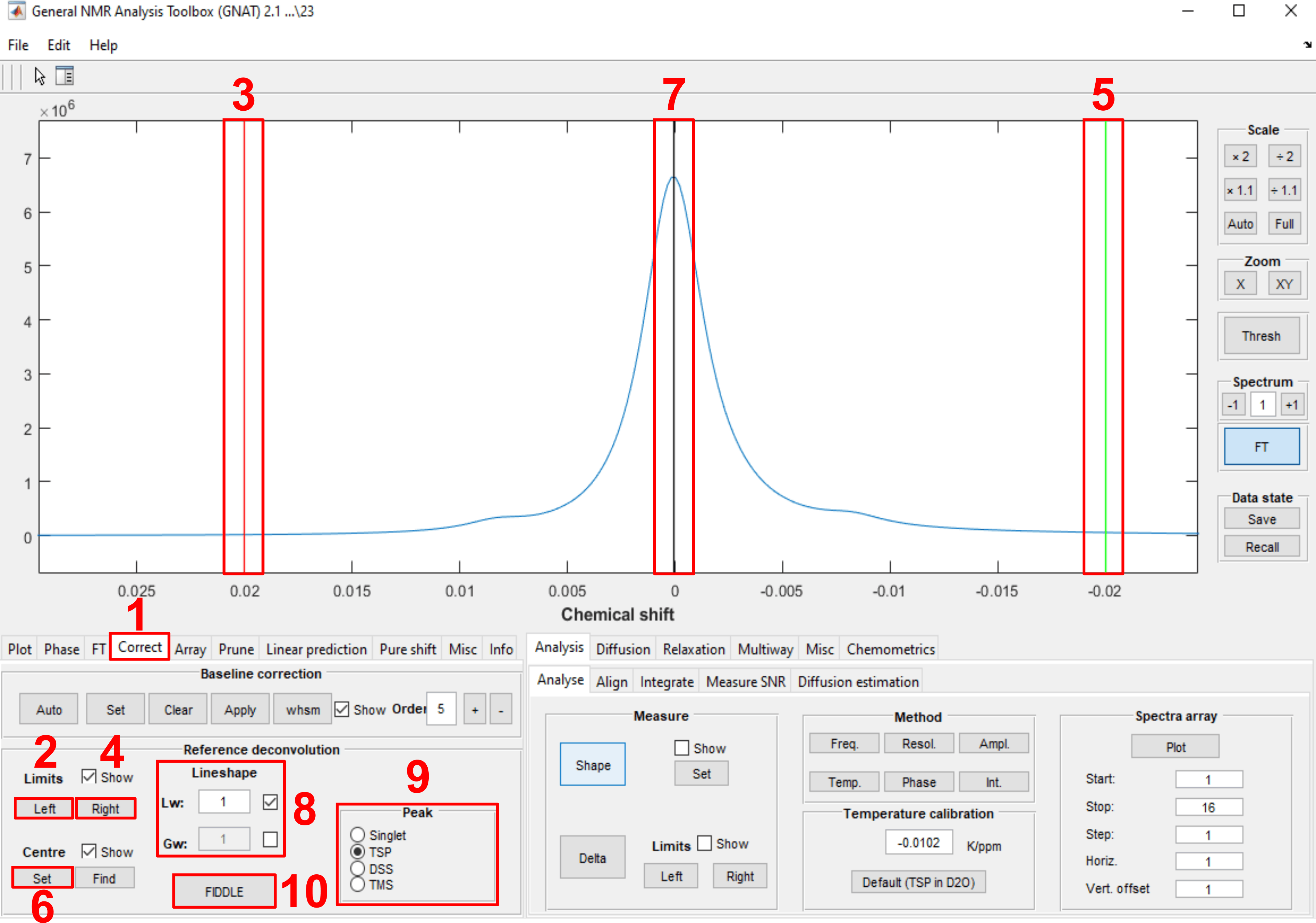


**Figure S9:** Reference deconvolution workflow.


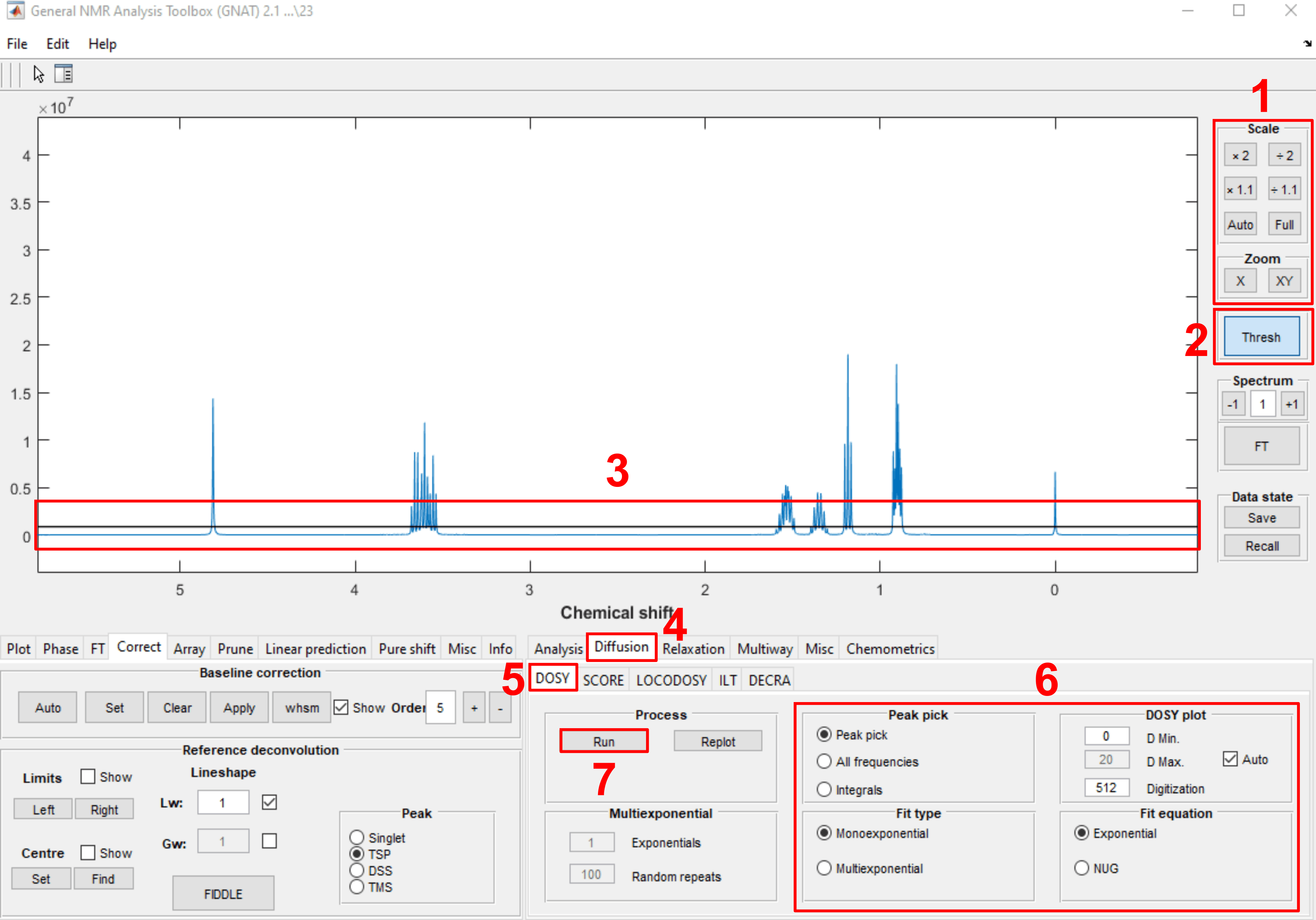


**Figure S10:** Generation of DOSY plot.


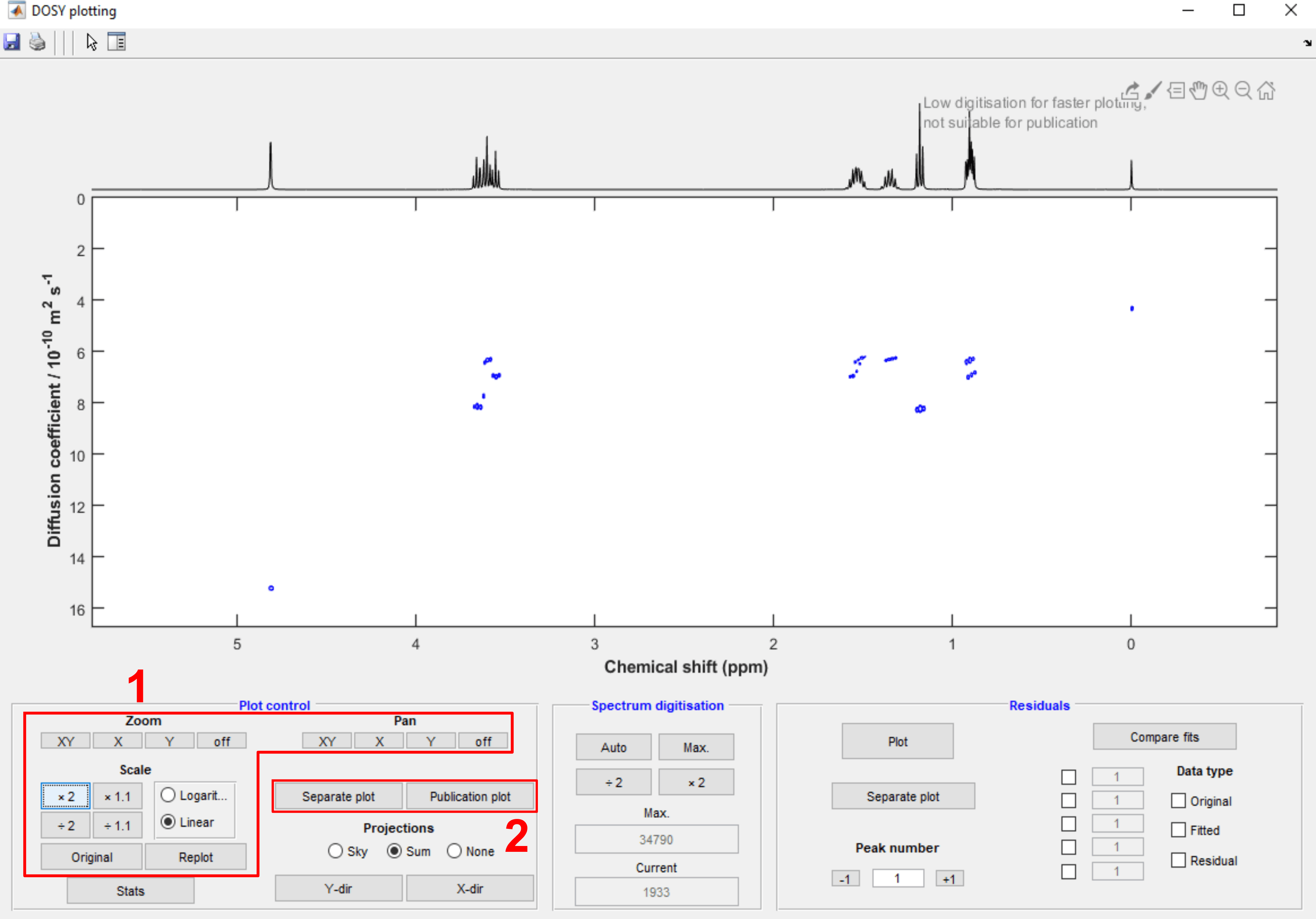


**Figure S11:** DOSY visualization interface.


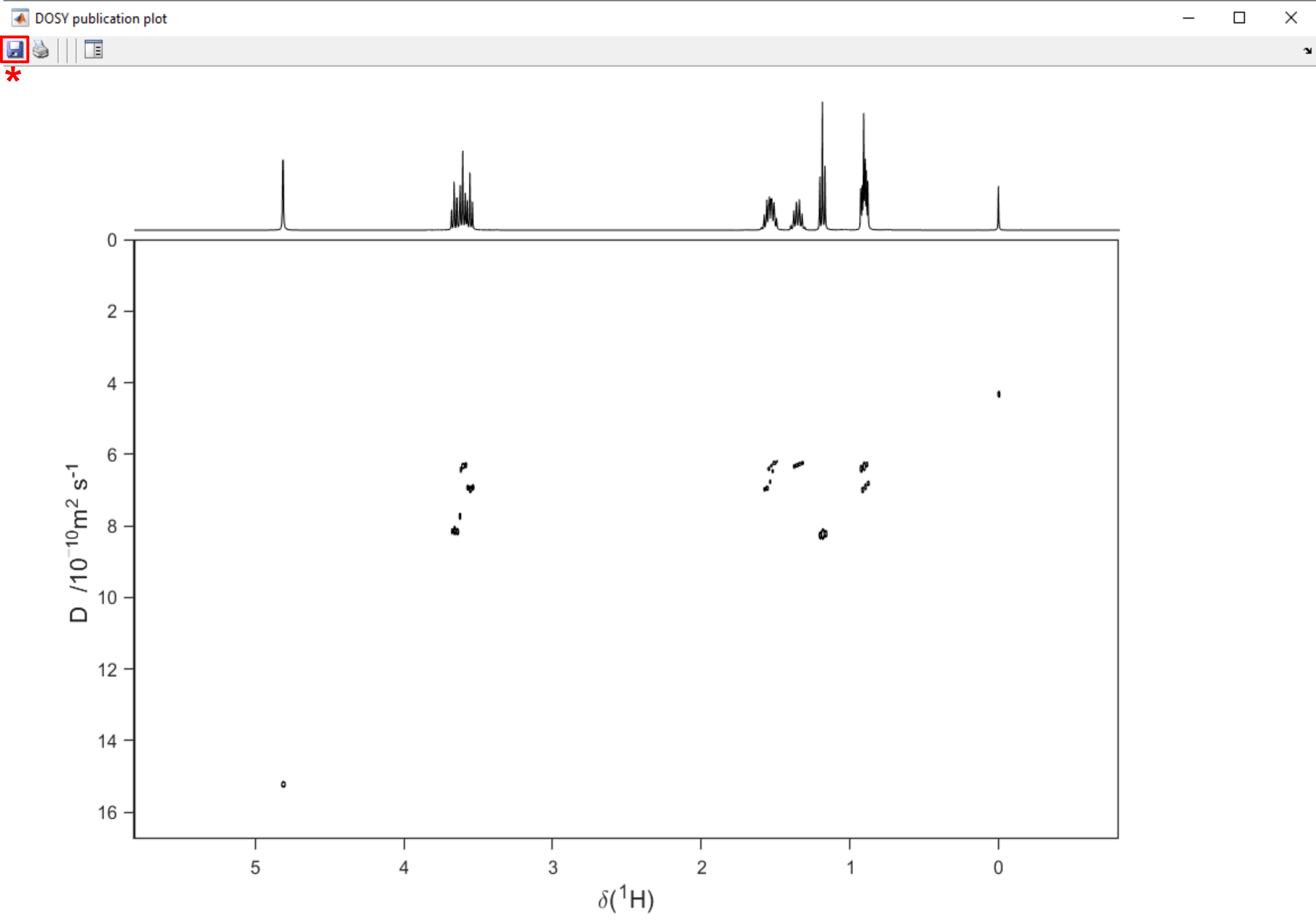


**Figure S12:** Exporting DOSY plots.


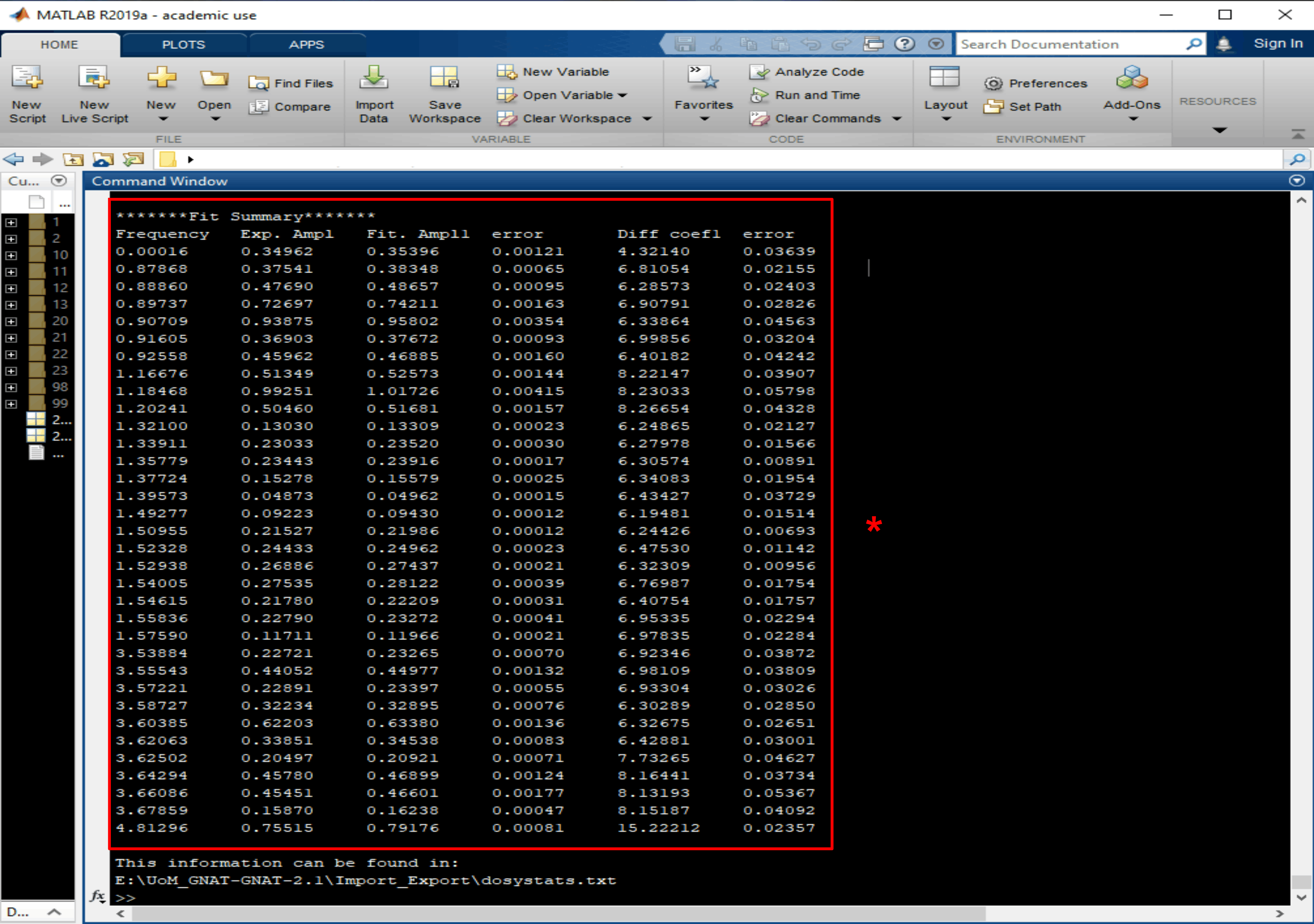


**Figure S13:** Terminal displaying the diffusion coefficients and fitting statistics.


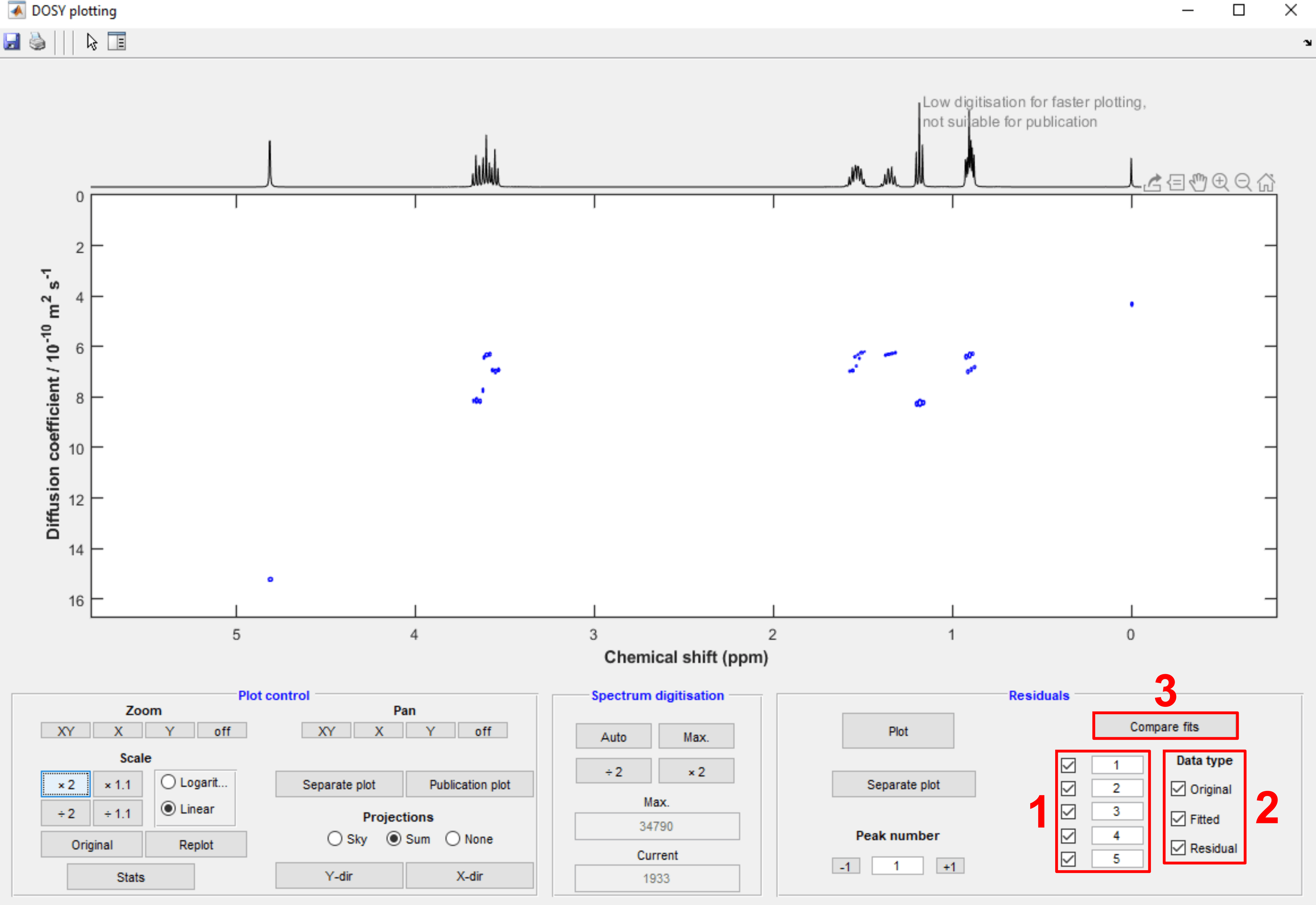


**Figure S14:** Access to further information on the fitting results.


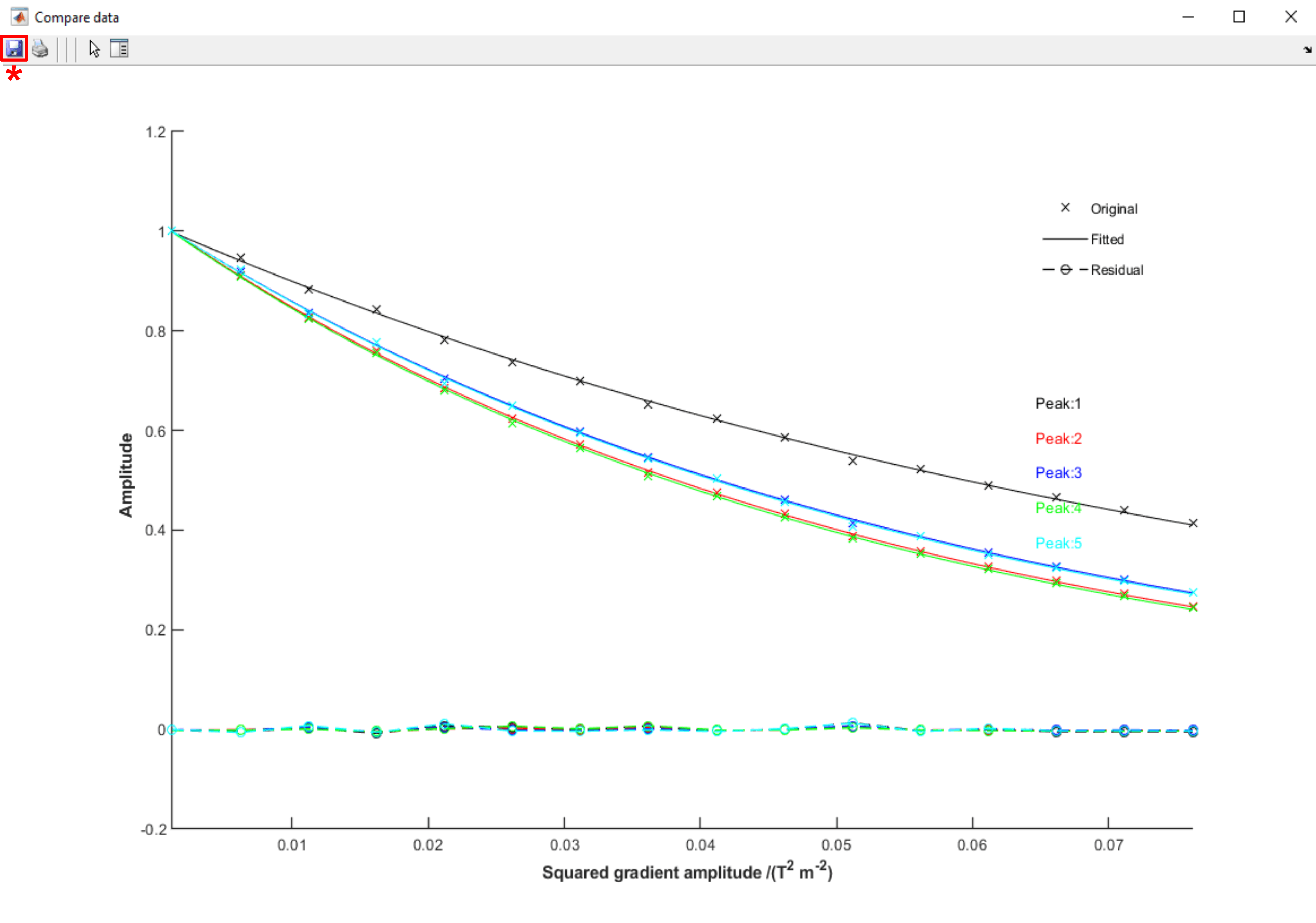


**Figure S15:** Fits and residuals for selected peaks.

# Further Information on DOSY Processing

The sections below present DOSY maps, fitting plots, diffusion coefficients, and the corresponding statistical parameters associated with the fitting procedures, compiled across different processing methods and reported exactly as obtained from the program. All fitting plots are based on the same signals shown and described in Figure 17 of the main text. The fit statistics are displayed as provided by GNAT; however, in formal scientific reporting, the number of significant figures should be selected to reflect a meaningful level of precision.

## Processing Without Window Functions

**(A) (B)**


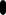

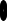

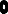

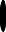

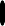


0 1.2


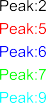


Original

Fitted Residual

1.0

4 0.8

D / 10-10 m2 s-1

0.6

Amplitude

8

0.4

12 0.2

0.0

16

3.8 3.7 3.6 3.5 3.4

-0.2

0.01

0.03

0.05

0.07


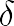
 (^1^H) Squared Gradient Amplitude / (T^2^ m^-2^)

**Figure S16: A)** 400 MHz ^1^H-DOSY plots and **B)** corresponding signal fitting plots for a mixture of ethanol, propanol, and butanol in D_2_O, focusing on the spectral region from 3.4 to 3.8 ppm, processed without any window function. In all cases, manual phasing for each increment and manual baseline correction were applied.

**Table S1:** 1H-DOSY (400 MHz) data for a mixture of propanol, butanol, and ethanol in D_2_O, processed without any window function. Chemical shifts correspond to the region from 3.4 to 3.8 ppm.

| **Compound Frequencya** | **Exp. Ampl.** | **Fit. Ampl.** | **Error** | **Diff. coef.b** | **Errorb** |
| --- | --- | --- | --- | --- | --- |
| 3.53871 | 0.18421 | 0.18819 | 0.00177 | 7.01266 | 0.12245 |
| Propanol 3.55549 | 0.35262 | 0.36221 | 0.00564 | 6.88877 | 0.20050 |
| 3.57208 | 0.18113 | 0.18486 | 0.00171 | 7.03241 | 0.12059 |
| 3.58714 | 0.22520 | 0.22931 | 0.00138 | 6.34566 | 0.07462 |
| Butanol 3.60373 | 0.45328 | 0.45998 | 0.00401 | 6.41184 | 0.10828 |
| 3.62050 | 0.22522 | 0.23063 | 0.00275 | 6.28175 | 0.14656 |
| 3.62508 | 0.15177 | 0.15498 | 0.00221 | 8.44485 | 0.20598 |
| Ethanol 3.64281 | 0.43903 | 0.44804 | 0.00743 | 8.51767 | 0.24119 |
| 3.66073 | 0.41749 | 0.43176 | 0.01012 | 8.07646 | 0.32973 |
| 3.67846 | 0.14551 | 0.14993 | 0.00274 | 8.20569 | 0.26000 |

a Chemical shift values in ppm.

b Diffusion coefficients are expressed in units of 10*^−^*^10^ m^2^ s^-1^.

## Processing with a Lorentzian Window Function (1 Hz)

**(A) (B)**


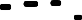

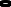

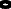

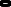


0 1.2


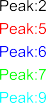


Original

Fitted Residual

1.0

4 0.8

D / 10-10 m2 s-1

0.6

Amplitude

8

0.4

12 0.2

0.0

16

3.8 3.7 3.6 3.5 3.4

-0.2

0.01

0.03

0.05

0.07


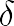
 (^1^H) Squared Gradient Amplitude / (T^2^ m^-2^)

**Figure S17: A)** 400 MHz ^1^H-DOSY plots and **B)** corresponding signal fitting plots for a mixture of ethanol, propanol, and butanol in D_2_O, focusing on the spectral region from 3.4 to 3.8 ppm, processed with a Lorentzian window function of 1 Hz. In all cases, manual phasing for each increment and manual baseline correction were applied.

**Table S2:** 1H-DOSY (400 MHz) data for a mixture of propanol, butanol, and ethanol in D_2_O, processed with a Lorentzian window function of 1 Hz. Chemical shifts correspond to the region from 3.4 to 3.8 ppm.

| **Compound Frequencya** | **Exp. Ampl.** | **Fit. Ampl.** | **Error** | **Diff. coef.b** | **Errorb** |
| --- | --- | --- | --- | --- | --- |
| 3.53871 | 0.22728 | 0.23278 | 0.00075 | 6.86878 | 0.04157 |
| Propanol 3.55530 | 0.44059 | 0.44990 | 0.00121 | 6.95609 | 0.03480 |
| 3.57208 | 0.22897 | 0.23406 | 0.00051 | 6.88939 | 0.02809 |
| 3.58714 | 0.32239 | 0.32904 | 0.00083 | 6.29312 | 0.03120 |
| Butanol 3.60373 | 0.62206 | 0.63394 | 0.00128 | 6.33093 | 0.02502 |
| 3.62050 | 0.33852 | 0.34552 | 0.00091 | 6.45562 | 0.03270 |
| 3.62489 | 0.20490 | 0.20931 | 0.00068 | 7.89430 | 0.04512 |
| Ethanol 3.64281 | 0.45777 | 0.46906 | 0.00120 | 8.19953 | 0.03622 |
| 3.66073 | 0.45445 | 0.46600 | 0.00189 | 8.18222 | 0.05751 |
| 3.67846 | 0.15863 | 0.16235 | 0.00046 | 8.28230 | 0.04024 |

a Chemical shift values in ppm.

b Diffusion coefficients are expressed in units of 10*^−^*^10^ m^2^ s^-1^.

## Processing with a Lorentzian Window Function (2 Hz)

**(A) (B)**


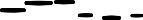

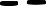


0 1.2


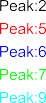


Original

Fitted Residual

1.0

4 0.8

D / 10-10 m2 s-1

0.6

Amplitude

8

0.4

12 0.2

0.0

16

3.8 3.7 3.6 3.5 3.4

-0.2

0.01

0.03

0.05

0.07


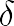
 (^1^H) Squared Gradient Amplitude / (T^2^ m^-2^)

**Figure S18: A)** 400 MHz ^1^H-DOSY plots and **B)** corresponding signal fitting plots for a mixture of ethanol, propanol, and butanol in D_2_O, focusing on the spectral region from 3.4 to 3.8 ppm, processed with a Lorentzian window function of 2 Hz. In all cases, manual phasing for each increment and manual baseline correction were applied.

**Table S3:** 1H-DOSY (400 MHz) data for a mixture of propanol, butanol, and ethanol in D_2_O, processed with a Lorentzian window function of 2 Hz. Chemical shifts correspond to the region from 3.4 to 3.8 ppm.

| **Compound** | **Frequencya** | **Exp. Ampl.** | **Fit. Ampl.** | **Error** | **Diff. coef.b** | **Errorb** |
| --- | --- | --- | --- | --- | --- | --- |
|  | 3.53852 | 0.23581 | 0.24155 | 0.00025 | 6.82635 | 0.01337 |
| Propanol | 3.55530 | 0.44085 | 0.45074 | 0.00047 | 6.91316 | 0.01354 |
|  | 3.57227 | 0.24652 | 0.25234 | 0.00033 | 6.81387 | 0.01651 |
|  | 3.58695 | 0.35028 | 0.35744 | 0.00035 | 6.30248 | 0.01206 |
| Butanol | 3.60373 | 0.64121 | 0.65393 | 0.00064 | 6.33495 | 0.01204 |
|  | 3.62069 | 0.38639 | 0.39421 | 0.00049 | 6.61663 | 0.01577 |
|  | 3.64281 | 0.45000 | 0.46116 | 0.00061 | 8.15947 | 0.01871 |
| Ethanol | 3.66073 | 0.44399 | 0.45478 | 0.00083 | 8.20286 | 0.02595 |
|  | 3.67846 | 0.16138 | 0.16490 | 0.00032 | 8.33761 | 0.02820 |

a Chemical shift values in ppm.

b Diffusion coefficients are expressed in units of 10*^−^*^10^ m^2^ s^-1^.

## Processing with a Gaussian Window Function (1 Hz)

**(A) (B)**


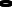

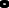


0 1.2


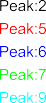


Original

Fitted Residual

1.0

4 0.8

D / 10-10 m2 s-1

0.6

Amplitude

8

0.4

12 0.2

0.0

16

3.8 3.7 3.6 3.5 3.4

-0.2

0.01

0.03

0.05

0.07


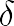
 (^1^H) Squared Gradient Amplitude / (T^2^ m^-2^)

**Figure S19: A)** 400 MHz ^1^H-DOSY plots and **B)** corresponding signal fitting plots for a mixture of ethanol, propanol, and butanol in D_2_O, focusing on the spectral region from 3.4 to 3.8 ppm, processed with a Gaussian window function of 1 Hz. In all cases, manual phasing for each increment and manual baseline correction were applied.

**Table S4:** 1H-DOSY (400 MHz) data for a mixture of propanol, butanol, and ethanol in D_2_O, processed with a Gaussian window function of 1 Hz. Chemical shifts correspond to the region from

3.4 to 3.8 ppm.

| **Compound Frequencya** | **Exp. Ampl.** | **Fit. Ampl.** | **Error** | **Diff. coef.b** | **Errorb** |
| --- | --- | --- | --- | --- | --- |
| 3.53871 | 0.22152 | 0.22680 | 0.00086 | 6.88904 | 0.04884 |
| Propanol 3.55530 | 0.43570 | 0.44484 | 0.00120 | 6.96262 | 0.03489 |
| 3.57208 | 0.21859 | 0.22340 | 0.00052 | 6.91677 | 0.02980 |
| 3.58714 | 0.31015 | 0.31651 | 0.00097 | 6.28895 | 0.03789 |
| Butanol 3.60373 | 0.61255 | 0.62421 | 0.00137 | 6.32300 | 0.02702 |
| 3.62050 | 0.31384 | 0.32049 | 0.00114 | 6.36009 | 0.04396 |
| 3.62489 | 0.16731 | 0.17106 | 0.00046 | 8.24366 | 0.03844 |
| Ethanol 3.64281 | 0.45212 | 0.46338 | 0.00116 | 8.19707 | 0.03562 |
| 3.66073 | 0.45123 | 0.46274 | 0.00184 | 8.17563 | 0.05622 |
| 3.67846 | 0.15459 | 0.15830 | 0.00042 | 8.25185 | 0.03779 |

a Chemical shift values in ppm.

b Diffusion coefficients are expressed in units of 10*^−^*^10^ m^2^ s^-1^.

## Processing with a Gaussian Window Function (2 Hz)

**(A) (B)**


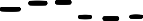

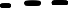


0 1.2


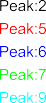


Original

Fitted Residual

1.0

4 0.8

D / 10-10 m2 s-1

0.6

Amplitude

8

0.4

12 0.2

0.0

16

3.8 3.7 3.6 3.5 3.4

-0.2

0.01

0.03

0.05

0.07


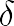
 (^1^H) Squared Gradient Amplitude / (T^2^ m^-2^)

**Figure S20: A)** 400 MHz ^1^H-DOSY plots and **B)** corresponding signal fitting plots for a mixture of ethanol, propanol, and butanol in D_2_O, focusing on the spectral region from 3.4 to 3.8 ppm, processed with a Gaussian window function of 2 Hz. In all cases, manual phasing for each increment and manual baseline correction were applied.

**Table S5:** 1H-DOSY (400 MHz) data for a mixture of propanol, butanol, and ethanol in D_2_O, processed with a Gaussian window function of 2 Hz. Chemical shifts correspond to the region from

3.4 to 3.8 ppm.

| **Compound** | **Frequencya** | **Exp. Ampl.** | **Fit. Ampl.** | **Error** | **Diff. coef.b** | **Errorb** |
| --- | --- | --- | --- | --- | --- | --- |
|  | 3.53852 | 0.23681 | 0.24251 | 0.00020 | 6.85293 | 0.01070 |
| Propanol | 3.55530 | 0.45724 | 0.46746 | 0.00042 | 6.92551 | 0.01173 |
|  | 3.57227 | 0.23327 | 0.23876 | 0.00030 | 6.86277 | 0.01610 |
|  | 3.58695 | 0.34387 | 0.35089 | 0.00027 | 6.27872 | 0.00947 |
| Butanol | 3.60373 | 0.66592 | 0.67916 | 0.00064 | 6.31486 | 0.01156 |
|  | 3.62089 | 0.37468 | 0.38228 | 0.00043 | 6.55167 | 0.01398 |
|  | 3.64281 | 0.46143 | 0.47307 | 0.00063 | 8.18430 | 0.01880 |
| Ethanol | 3.66073 | 0.46186 | 0.47319 | 0.00074 | 8.20289 | 0.02212 |
|  | 3.67865 | 0.15861 | 0.16228 | 0.00031 | 8.30301 | 0.02750 |

a Chemical shift values in ppm.

b Diffusion coefficients are expressed in units of 10*^−^*^10^ m^2^ s^-1^.

## Processing with a Combined Lorentzian and Gaussian Window Functions (1 Hz)

**(A) (B)**


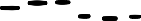

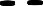


0 1.2


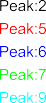


Original

Fitted Residual

1.0

4 0.8

D / 10-10 m2 s-1

0.6

Amplitude

8

0.4

12 0.2

0.0

16

3.8 3.7 3.6 3.5 3.4

-0.2

0.01

0.03

0.05

0.07


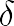
 (^1^H) Squared Gradient Amplitude / (T^2^ m^-2^)

**Figure S21: A)** 400 MHz ^1^H-DOSY plots and **B)** corresponding signal fitting plots for a mixture of ethanol, propanol, and butanol in D_2_O, focusing on the spectral region from 3.4 to 3.8 ppm, processed with a combined Lorentzian and Gaussian window functions of 1 Hz. In all cases, manual phasing for each increment and manual baseline correction were applied.

**Table S6:** 1H-DOSY (400 MHz) data for a mixture of propanol, butanol, and ethanol in D_2_O, processed with a combined Lorentzian and Gaussian window functions of 1 Hz. Chemical shifts correspond to the region from 3.4 to 3.8 ppm.

| **Compound** | **Frequencya** | **Exp. Ampl.** | **Fit. Ampl.** | **Error** | **Diff. coef.b** | **Errorb** |
| --- | --- | --- | --- | --- | --- | --- |
|  | 3.53852 | 0.23810 | 0.24373 | 0.00028 | 6.85799 | 0.01482 |
| Propanol | 3.55530 | 0.45520 | 0.46527 | 0.00059 | 6.92802 | 0.01647 |
|  | 3.57227 | 0.23923 | 0.24490 | 0.00048 | 6.84705 | 0.02513 |
|  | 3.58695 | 0.34619 | 0.35311 | 0.00043 | 6.29541 | 0.01510 |
| Butanol | 3.60373 | 0.65797 | 0.67094 | 0.00079 | 6.32346 | 0.01459 |
|  | 3.62050 | 0.37070 | 0.37807 | 0.00042 | 6.51452 | 0.01381 |
|  | 3.64281 | 0.46393 | 0.47553 | 0.00072 | 8.17582 | 0.02151 |
| Ethanol | 3.66073 | 0.46169 | 0.47306 | 0.00098 | 8.19665 | 0.02951 |
|  | 3.67846 | 0.16205 | 0.16571 | 0.00032 | 8.30747 | 0.02742 |

a Chemical shift values in ppm.

b Diffusion coefficients are expressed in units of 10*^−^*^10^ m^2^ s^-1^.

## Reference Deconvolution Based on a Lorentzian Window Function (1 Hz)

**(A) (B)**


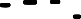


0 1.2


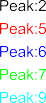


Original

Fitted Residual

1.0

4 0.8

D / 10-10 m2 s-1

0.6

Amplitude

8

0.4

12 0.2

0.0

16

3.8 3.7 3.6 3.5 3.4

-0.2

0.01

0.03

0.05

0.07


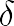
 (^1^H) Squared Gradient Amplitude / (T^2^ m^-2^)

**Figure S22: A)** 400 MHz ^1^H-DOSY plots and **B)** corresponding signal fitting plots for a mixture of ethanol, propanol, and butanol in D_2_O, focusing on the spectral region from 3.4 to 3.8 ppm, processed with reference deconvolution based on a Lorentzian window function of 1 Hz. In all cases, manual phasing for each increment and manual baseline correction were applied.

**Table S7:** 1H-DOSY (400 MHz) data for a mixture of propanol, butanol, and ethanol in D_2_O, processed with reference deconvolution based on a Lorentzian window function of 1 Hz. Chemical shifts correspond to the region from 3.4 to 3.8 ppm.

| **Compound Frequencya** | **Exp. Ampl.** | **Fit. Ampl.** | **Error** | **Diff. coef.b** | **Errorb** |
| --- | --- | --- | --- | --- | --- |
| 3.53852 | 0.21579 | 0.22078 | 0.00008 | 6.88105 | 0.00479 |
| Propanol 3.55511 | 0.42273 | 0.43219 | 0.00016 | 6.92573 | 0.00486 |
| 3.57189 | 0.21611 | 0.22097 | 0.00009 | 6.90012 | 0.00511 |
| 3.58695 | 0.29768 | 0.30376 | 0.00011 | 6.28715 | 0.00441 |
| Butanol 3.60354 | 0.58021 | 0.59183 | 0.00021 | 6.30817 | 0.00441 |
| 3.62012 | 0.30950 | 0.31559 | 0.00018 | 6.42831 | 0.00694 |
| 3.62470 | 0.18895 | 0.19339 | 0.00016 | 7.80526 | 0.01165 |
| Ethanol 3.64262 | 0.45063 | 0.46199 | 0.00029 | 8.15388 | 0.00878 |
| 3.66035 | 0.45243 | 0.46356 | 0.00041 | 8.17609 | 0.01250 |
| 3.67827 | 0.15405 | 0.15770 | 0.00018 | 8.24027 | 0.01585 |

a Chemical shift values in ppm.

b Diffusion coefficients are expressed in units of 10*^−^*^10^ m^2^ s^-1^.

## Reference Deconvolution Based on a Gaussian Window Function (1 Hz)

**(A) (B)**

0 1.2


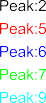


Original

Fitted Residual

1.0

4 0.8

D / 10-10 m2 s-1

0.6

Amplitude

8

0.4

12 0.2

0.0

16

3.8 3.7 3.6 3.5 3.4

-0.2

0.01

0.03

0.05

0.07


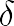
 (^1^H) Squared Gradient Amplitude / (T^2^ m^-2^)

**Figure S23: A)** 400 MHz ^1^H-DOSY plots and **B)** corresponding signal fitting plots for a mixture of ethanol, propanol, and butanol in D_2_O, focusing on the spectral region from 3.4 to 3.8 ppm, processed with reference deconvolution based on a Gaussian window function of 1 Hz. In all cases, manual phasing for each increment and manual baseline correction were applied.

**Table S8:** 1H-DOSY (400 MHz) data for a mixture of propanol, butanol, and ethanol in D_2_O, processed with reference deconvolution based on a Gaussian window function of 1 Hz. Chemical shifts correspond to the region from 3.4 to 3.8 ppm.

| **Compound Frequencya** | **Exp. Ampl.** | **Fit. Ampl.** | **Error** | **Diff. coef.b** | **Errorb** |
| --- | --- | --- | --- | --- | --- |
| 3.53852 | 0.21446 | 0.21935 | 0.00008 | 6.89769 | 0.00457 |
| Propanol 3.55511 | 0.42489 | 0.43434 | 0.00017 | 6.93320 | 0.00516 |
| 3.57189 | 0.21122 | 0.21594 | 0.00008 | 6.92066 | 0.00505 |
| 3.58695 | 0.29466 | 0.30063 | 0.00011 | 6.28290 | 0.00436 |
| Butanol 3.60354 | 0.58541 | 0.59712 | 0.00021 | 6.30235 | 0.00442 |
| 3.62012 | 0.29659 | 0.30244 | 0.00014 | 6.35267 | 0.00584 |
| 3.62470 | 0.15943 | 0.16342 | 0.00010 | 8.06939 | 0.00899 |
| Ethanol 3.64262 | 0.44739 | 0.45874 | 0.00027 | 8.15800 | 0.00840 |
| 3.66035 | 0.45069 | 0.46185 | 0.00039 | 8.17243 | 0.01200 |
| 3.67827 | 0.15196 | 0.15565 | 0.00015 | 8.21761 | 0.01376 |

a Chemical shift values in ppm.

b Diffusion coefficients are expressed in units of 10*^−^*^10^ m^2^ s^-1^.

## Reference Deconvolution Based on a Combined Lorentzian Window Function (-0.3 Hz) and Gaussian (1 Hz)

**(A) (B)**

0 1.2


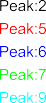


Original

Fitted Residual

1.0

4 0.8

D / 10-10 m2 s-1

0.6

Amplitude

8

0.4

12 0.2

0.0

16

3.8 3.7 3.6 3.5 3.4

-0.2

0.01

0.03

0.05

0.07


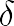
 (^1^H) Squared Gradient Amplitude / (T^2^ m^-2^)

**Figure S24: A)** 400 MHz ^1^H-DOSY plots and **B)** corresponding signal fitting plots for a mixture of ethanol, propanol, and butanol in D_2_O, focusing on the spectral region from 3.4 to 3.8 ppm, processed with reference deconvolution based on a Lorentzian window function -0.3 Hz and a Gaussian window function of 1 Hz. In all cases, manual phasing for each increment and manual baseline correction were applied.

**Table S9:** 1H-DOSY (400 MHz) data for a mixture of propanol, butanol, and ethanol in D_2_O, processed with reference deconvolution based on a Lorentzian window function -0.3 Hz and a Gaussian window function of 1 Hz. Chemical shifts correspond to the region from 3.4 to 3.8 ppm.

| **Compound Frequencya** | **Exp. Ampl.** | **Fit. Ampl.** | **Error** | **Diff. coef.b** | **Errorb** |
| --- | --- | --- | --- | --- | --- |
| 3.53852 | 0.20788 | 0.21256 | 0.00008 | 6.90745 | 0.00502 |
| Propanol 3.55511 | 0.41434 | 0.42351 | 0.00017 | 6.93529 | 0.00533 |
| 3.57189 | 0.20417 | 0.20870 | 0.00009 | 6.92779 | 0.00533 |
| 3.58695 | 0.27930 | 0.28492 | 0.00011 | 6.28418 | 0.00459 |
| Butanol 3.60354 | 0.55894 | 0.57011 | 0.00020 | 6.29896 | 0.00441 |
| 3.62012 | 0.27993 | 0.28545 | 0.00013 | 6.32872 | 0.00551 |
| 3.62489 | 0.14860 | 0.15238 | 0.00009 | 8.14927 | 0.00869 |
| Ethanol 3.64262 | 0.44311 | 0.45436 | 0.00027 | 8.15440 | 0.00826 |
| 3.66035 | 0.44713 | 0.45823 | 0.00038 | 8.16595 | 0.01174 |
| 3.67827 | 0.14965 | 0.15332 | 0.00014 | 8.19978 | 0.01274 |

a Chemical shift values in ppm.

b Diffusion coefficients are expressed in units of 10*^−^*^10^ m^2^ s^-1^.

## Reference Deconvolution Based on a Combined Lorentzian and Gaussian Window Functions (1 Hz)

**(A) (B)**


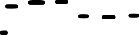


0 1.2


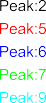


Original

Fitted Residual

1.0

4 0.8

D / 10-10 m2 s-1

0.6

Amplitude

8

0.4

12 0.2

0.0

16

3.8 3.7 3.6 3.5 3.4

-0.2

0.01

0.03

0.05

0.07


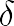
 (^1^H) Squared Gradient Amplitude / (T^2^ m^-2^)

**Figure S25: A)** 400 MHz ^1^H-DOSY plots and **B)** corresponding signal fitting plots for a mixture of ethanol, propanol, and butanol in D_2_O, focusing on the spectral region from 3.4 to 3.8 ppm, processed with reference deconvolution based on a combined Lorentzian window function and Gaussian window function of 1 Hz. In all cases, manual phasing for each increment and manual baseline correction were applied.

**Table S10:** 1H-DOSY (400 MHz) data for a mixture of propanol, butanol, and ethanol in D_2_O, processed with reference deconvolution based on a combined Lorentzian window function and Gaussian window function of 1 Hz. Chemical shifts correspond to the region from 3.4 to 3.8 ppm.

| **Compound Frequencya** | **Exp. Ampl.** | **Fit. Ampl.** | **Error** | **Diff. coef.b** | **Errorb** |
| --- | --- | --- | --- | --- | --- |
| 3.53833 | 0.23409 | 0.23965 | 0.00009 | 6.86122 | 0.00462 |
| Propanol 3.55511 | 0.44966 | 0.45981 | 0.00016 | 6.92142 | 0.00462 |
| 3.57189 | 0.23417 | 0.23950 | 0.00009 | 6.88588 | 0.00471 |
| 3.58676 | 0.33728 | 0.34426 | 0.00010 | 6.28378 | 0.00353 |
| Butanol 3.60354 | 0.64497 | 0.65791 | 0.00024 | 6.31460 | 0.00447 |
| 3.62031 | 0.35200 | 0.35890 | 0.00023 | 6.48405 | 0.00788 |
| 3.62413 | 0.22136 | 0.22630 | 0.00022 | 7.56196 | 0.01299 |
| Ethanol 3.64262 | 0.46089 | 0.47248 | 0.00030 | 8.16157 | 0.00908 |
| 3.66054 | 0.45811 | 0.46930 | 0.00044 | 8.19210 | 0.01318 |
| 3.67827 | 0.16014 | 0.16385 | 0.00021 | 8.27710 | 0.01803 |

a Chemical shift values in ppm.

b Diffusion coefficients are expressed in units of 10*^−^*^10^ m^2^ s^-1^.

## Processing with a Lorentzian Window Function (0.5 Hz)

**(A) (B)**


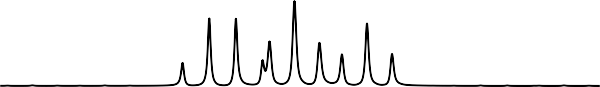

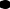

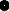

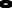

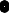

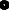


0 1.2


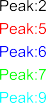


Original

Fitted Residual

1.0

4 0.8

D / 10-10 m2 s-1

0.6

Amplitude

8

0.4

12 0.2

0.0

16

3.8 3.7 3.6 3.5 3.4

-0.2

0.01

0.03

0.05

0.07


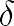
 (^1^H) Squared Gradient Amplitude / (T^2^ m^-2^)

**Figure S26: A)** 400 MHz ^1^H-DOSY plots and **B)** corresponding signal fitting plots for a mixture of ethanol, propanol, and butanol in D_2_O, focusing on the spectral region from 3.4 to 3.8 ppm, processed with a Lorentzian window function of 0.5 Hz. In all cases, manual phasing for each increment and manual baseline correction were applied.

**Table S11:** 1H-DOSY (400 MHz) data for a mixture of propanol, butanol, and ethanol in D_2_O, processed with a Lorentzian window function of 0.5 Hz. Chemical shifts correspond to the region from 3.4 to 3.8 ppm.

| **Compound Frequencya** | **Exp. Ampl.** | **Fit. Ampl.** | **Error** | **Diff. coef.b** | **Errorb** |
| --- | --- | --- | --- | --- | --- |
| 3.53871 | 0.21246 | 0.21732 | 0.00105 | 6.92800 | 0.06219 |
| Propanol 3.55549 | 0.40987 | 0.41993 | 0.00313 | 6.90966 | 0.09605 |
| 3.57208 | 0.21088 | 0.21535 | 0.00087 | 6.95160 | 0.05232 |
| 3.58714 | 0.28767 | 0.29339 | 0.00101 | 6.30636 | 0.04235 |
| Butanol 3.60373 | 0.56639 | 0.57656 | 0.00216 | 6.34441 | 0.04635 |
| 3.62050 | 0.29338 | 0.29986 | 0.00169 | 6.35761 | 0.06970 |
| 3.62508 | 0.17078 | 0.17501 | 0.00102 | 8.04767 | 0.08159 |
| Ethanol 3.64281 | 0.44870 | 0.45951 | 0.00251 | 8.25054 | 0.07772 |
| 3.66073 | 0.44260 | 0.45487 | 0.00362 | 8.13745 | 0.11239 |
| 3.67846 | 0.15291 | 0.15691 | 0.00086 | 8.20548 | 0.07778 |

a Chemical shift values in ppm.

b Diffusion coefficients are expressed in units of 10*^−^*^10^ m^2^ s^-1^.

## Processing with a Gaussian Window Function (0.5 Hz)

**(A) (B)**


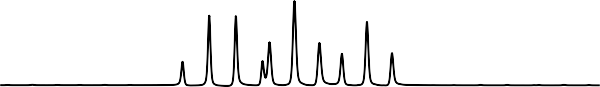

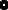

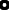

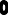

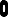

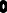


0 1.2


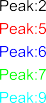


Original

Fitted Residual

1.0

4 0.8

D / 10-10 m2 s-1

0.6

Amplitude

8

0.4

12 0.2

0.0

16

3.8 3.7 3.6 3.5 3.4

-0.2

0.01

0.03

0.05

0.07


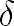
 (^1^H) Squared Gradient Amplitude / (T^2^ m^-2^)

**Figure S27: A)** 400 MHz ^1^H-DOSY plots and **B)** corresponding signal fitting plots for a mixture of ethanol, propanol, and butanol in D_2_O, focusing on the spectral region from 3.4 to 3.8 ppm, processed with a Gaussian window function of 0.5 Hz. In all cases, manual phasing for each increment and manual baseline correction were applied.

**Table S12:** 1H-DOSY (400 MHz) data for a mixture of propanol, butanol, and ethanol in D_2_O, processed with a Gaussian window function of 0.5 Hz. Chemical shifts correspond to the region from

3.4 to 3.8 ppm.

| **Compound Frequencya** | **Exp. Ampl.** | **Fit. Ampl.** | **Error** | **Diff. coef.b** | **Errorb** |
| --- | --- | --- | --- | --- | --- |
| 3.53871 | 0.20564 | 0.21031 | 0.00122 | 6.94175 | 0.07492 |
| Propanol 3.55549 | 0.39831 | 0.40830 | 0.00371 | 6.90525 | 0.11721 |
| 3.57208 | 0.20251 | 0.20676 | 0.00103 | 6.97303 | 0.06451 |
| 3.58714 | 0.27140 | 0.27670 | 0.00116 | 6.31182 | 0.05176 |
| Butanol 3.60373 | 0.54070 | 0.55009 | 0.00261 | 6.35305 | 0.05868 |
| 3.62031 | 0.27670 | 0.28150 | 0.00143 | 6.39363 | 0.06326 |
| 3.62508 | 0.15525 | 0.15916 | 0.00099 | 8.21413 | 0.08847 |
| Ethanol 3.64281 | 0.44409 | 0.45470 | 0.00292 | 8.27199 | 0.09160 |
| 3.66073 | 0.43762 | 0.44995 | 0.00418 | 8.13209 | 0.13134 |
| 3.67846 | 0.15036 | 0.15433 | 0.00098 | 8.20306 | 0.09058 |

a Chemical shift values in ppm.

b Diffusion coefficients are expressed in units of 10*^−^*^10^ m^2^ s^-1^.

## Processing with a Combined Lorentzian Window Function (-0.5 Hz) and Gaussian (0.5 Hz)

**(A) (B)**


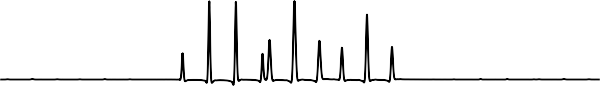

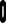

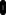

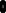

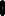

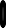

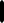

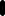

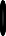

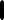

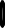


0 1.2


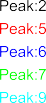


Original

Fitted Residual

1.0

4 0.8

D / 10-10 m2 s-1

0.6

Amplitude

8

0.4

12 0.2

0.0

16

3.8 3.7 3.6 3.5 3.4

-0.2

0.01

0.03

0.05

0.07


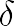
 (^1^H) Squared Gradient Amplitude / (T^2^ m^-2^)

**Figure S28: A)** 400 MHz ^1^H-DOSY plots and **B)** corresponding signal fitting plots for a mixture of ethanol, propanol, and butanol in D_2_O, focusing on the spectral region from 3.4 to 3.8 ppm, processed with a combined Lorentzian window function of -0.5 Hz and Gaussian width of 0.5 Hz. In all cases, manual phasing for each increment and manual baseline correction were applied.

**Table S13:** 1H-DOSY (400 MHz) data for a mixture of propanol, butanol, and ethanol in D_2_O, processed with a combined Lorentzian window function of -0.5 Hz and Gaussian width of 0.5 Hz. Chemical shifts correspond to the region from 3.4 to 3.8 ppm.

| **Compound Frequencya** | **Exp. Ampl.** | **Fit. Ampl.** | **Error** | **Diff. coef.b** | **Errorb** |
| --- | --- | --- | --- | --- | --- |
| 3.53871 | 0.18242 | 0.18634 | 0.00189 | 7.01517 | 0.13187 |
| Propanol 3.55549 | 0.34992 | 0.35970 | 0.00610 | 6.88342 | 0.21842 |
| 3.57208 | 0.17878 | 0.18240 | 0.00180 | 7.04171 | 0.12874 |
| 3.58714 | 0.21714 | 0.22102 | 0.00151 | 6.35393 | 0.08481 |
| Butanol 3.60392 | 0.42528 | 0.43586 | 0.00598 | 6.23961 | 0.16848 |
| 3.62031 | 0.22240 | 0.22572 | 0.00208 | 6.42285 | 0.11452 |
| 3.62508 | 0.14436 | 0.14778 | 0.00192 | 8.46668 | 0.18799 |
| Ethanol 3.64281 | 0.43494 | 0.44426 | 0.00666 | 8.47321 | 0.21728 |
| 3.66073 | 0.41761 | 0.43131 | 0.00882 | 8.09410 | 0.28799 |
| 3.67846 | 0.14462 | 0.14887 | 0.00226 | 8.21487 | 0.21588 |

a Chemical shift values in ppm.

b Diffusion coefficients are expressed in units of 10*^−^*^10^ m^2^ s^-1^.

## Processing with a Combined Lorentzian Window Function (0.5 Hz) and Gaussian (0.5 Hz)

**(A) (B)**


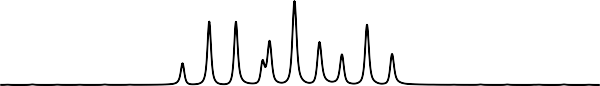

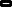

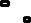

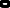

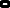

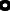

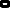


0 1.2


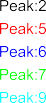


Original

Fitted Residual

1.0

4 0.8

D / 10-10 m2 s-1

0.6

Amplitude

8

0.4

12 0.2

0.0

16

3.8 3.7 3.6 3.5 3.4

-0.2

0.01

0.03

0.05

0.07


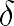
 (^1^H) Squared Gradient Amplitude / (T^2^ m^-2^)

**Figure S29: A)** 400 MHz ^1^H-DOSY plots and **B)** corresponding signal fitting plots for a mixture of ethanol, propanol, and butanol in D_2_O, focusing on the spectral region from 3.4 to 3.8 ppm, processed with a combined Lorentzian window function of 0.5 Hz and Gaussian width of 0.5 Hz. In all cases, manual phasing for each increment and manual baseline correction were applied.

**Table S14:** 1H-DOSY (400 MHz) data for a mixture of propanol, butanol, and ethanol in D_2_O, processed with a combined Lorentzian window function of 0.5 Hz and Gaussian width of 0.5 Hz. Chemical shifts correspond to the region from 3.4 to 3.8 ppm.

| **Compound Frequencya** | **Exp. Ampl.** | **Fit. Ampl.** | **Error** | **Diff. coef.b** | **Errorb** |
| --- | --- | --- | --- | --- | --- |
| 3.53871 | 0.22031 | 0.22542 | 0.00087 | 6.90981 | 0.04956 |
| Propanol 3.55530 | 0.43142 | 0.44015 | 0.00161 | 6.98502 | 0.04730 |
| 3.57208 | 0.21905 | 0.22375 | 0.00063 | 6.93318 | 0.03643 |
| 3.58714 | 0.30617 | 0.31238 | 0.00092 | 6.29689 | 0.03623 |
| Butanol 3.60373 | 0.60020 | 0.61144 | 0.00162 | 6.32963 | 0.03280 |
| 3.62050 | 0.31392 | 0.32064 | 0.00125 | 6.37762 | 0.04820 |
| 3.62489 | 0.17931 | 0.18332 | 0.00058 | 8.02940 | 0.04418 |
| Ethanol 3.64281 | 0.45222 | 0.46347 | 0.00149 | 8.19744 | 0.04569 |
| 3.66073 | 0.44922 | 0.46110 | 0.00225 | 8.14857 | 0.06889 |
| 3.67846 | 0.15497 | 0.15890 | 0.00052 | 8.20341 | 0.04654 |

a Chemical shift values in ppm.

b Diffusion coefficients are expressed in units of 10*^−^*^10^ m^2^ s^-1^.

## Reference Deconvolution Based on a Lorentzian Window Function (0.5 Hz)

**(A) (B)**


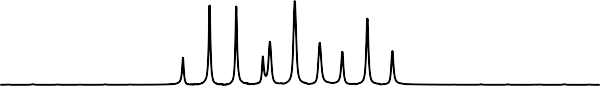


0 1.2


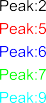


Original

Fitted Residual

1.0

4 0.8

D / 10-10 m2 s-1

0.6

Amplitude

8

0.4

12 0.2

0.0

16

3.8 3.7 3.6 3.5 3.4

-0.2

0.01

0.03

0.05

0.07


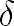
 (^1^H) Squared Gradient Amplitude / (T^2^ m^-2^)

**Figure S30: A)** 400 MHz ^1^H-DOSY plots and **B)** corresponding signal fitting plots for a mixture of ethanol, propanol, and butanol in D_2_O, focusing on the spectral region from 3.4 to 3.8 ppm, processed using reference deconvolution based on a Lorentzian window function of 0.5 Hz. In all cases, manual phasing for each increment and manual baseline correction were applied.

**Table S15:** 1H-DOSY (400 MHz) data for a mixture of propanol, butanol, and ethanol in D_2_O, processed using reference deconvolution based on a Lorentzian window function of 0.5 Hz. Chemical shifts correspond to the region from 3.4 to 3.8 ppm.

| **Compound Frequencya** | **Exp. Ampl.** | **Fit. Ampl.** | **Error** | **Diff. coef.b** | **Errorb** |
| --- | --- | --- | --- | --- | --- |
| 3.53852 | 0.18431 | 0.18831 | 0.00011 | 6.91970 | 0.00753 |
| Propanol 3.55511 | 0.36761 | 0.37567 | 0.00017 | 6.93113 | 0.00597 |
| 3.57189 | 0.18210 | 0.18606 | 0.00011 | 6.92682 | 0.00744 |
| 3.58695 | 0.23202 | 0.23660 | 0.00011 | 6.28718 | 0.00570 |
| Butanol 3.60354 | 0.46500 | 0.47424 | 0.00017 | 6.28847 | 0.00444 |
| 3.62012 | 0.23976 | 0.24454 | 0.00012 | 6.35251 | 0.00612 |
| 3.62489 | 0.15764 | 0.16149 | 0.00013 | 7.99611 | 0.01132 |
| Ethanol 3.64262 | 0.43534 | 0.44636 | 0.00029 | 8.13122 | 0.00907 |
| 3.66035 | 0.44049 | 0.45145 | 0.00038 | 8.14048 | 0.01205 |
| 3.67827 | 0.14107 | 0.14454 | 0.00014 | 8.15774 | 0.01346 |

a Chemical shift values in ppm.

b Diffusion coefficients are expressed in units of 10*^−^*^10^ m^2^ s^-1^.

## Reference Deconvolution Based on a Gaussian Window Function (0.5 Hz)

**(A) (B)**


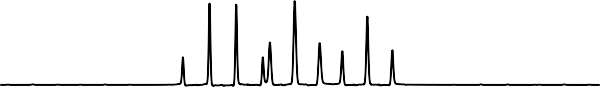


0 1.2


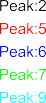


Original

Fitted Residual

1.0

4 0.8

D / 10-10 m2 s-1

0.6

Amplitude

8

0.4

12 0.2

0.0

16

3.8 3.7 3.6 3.5 3.4

-0.2

0.01

0.03

0.05

0.07


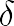
 (^1^H) Squared Gradient Amplitude / (T^2^ m^-2^)

**Figure S31: A)** 400 MHz ^1^H-DOSY plots and **B)** corresponding signal fitting plots for a mixture of ethanol, propanol, and butanol in D_2_O, focusing on the spectral region from 3.4 to 3.8 ppm, processed using reference deconvolution based on a Gaussian window function of 0.5 Hz. In all cases, manual phasing for each increment and manual baseline correction were applied.

**Table S16:** 1H-DOSY (400 MHz) data for a mixture of propanol, butanol, and ethanol in D_2_O, processed using reference deconvolution based on a Gaussian window function of 0.5 Hz. Chemical shifts correspond to the region from 3.4 to 3.8 ppm.

| **Compound Frequencya** | **Exp. Ampl.** | **Fit. Ampl.** | **Error** | **Diff. coef.b** | **Errorb** |
| --- | --- | --- | --- | --- | --- |
| 3.53852 | 0.18217 | 0.18611 | 0.00011 | 6.92355 | 0.00796 |
| Propanol 3.55511 | 0.36646 | 0.37448 | 0.00017 | 6.93141 | 0.00592 |
| 3.57189 | 0.17902 | 0.18290 | 0.00011 | 6.93169 | 0.00770 |
| 3.58695 | 0.22239 | 0.22679 | 0.00011 | 6.28705 | 0.00590 |
| Butanol 3.60354 | 0.44953 | 0.45852 | 0.00017 | 6.28614 | 0.00452 |
| 3.62012 | 0.22817 | 0.23271 | 0.00011 | 6.32007 | 0.00601 |
| 3.62489 | 0.14696 | 0.15064 | 0.00012 | 8.09252 | 0.01082 |
| Ethanol 3.64262 | 0.43105 | 0.44199 | 0.00027 | 8.13299 | 0.00855 |
| 3.66035 | 0.43656 | 0.44745 | 0.00037 | 8.14064 | 0.01161 |
| 3.67827 | 0.14108 | 0.14458 | 0.00013 | 8.15649 | 0.01271 |

a Chemical shift values in ppm.

b Diffusion coefficients are expressed in units of 10*^−^*^10^ m^2^ s^-1^.

## Reference Deconvolution Based on a Combined Lorentzian Window Function (-0.5 Hz) and Gaussian (0.5 Hz)

**(A) (B)**


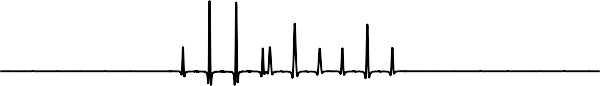


0 1.2


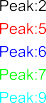


Original

Fitted Residual

1.0

4 0.8

D / 10-10 m2 s-1

0.6

Amplitude

8

0.4

12 0.2

0.0

16

3.8 3.7 3.6 3.5 3.4

-0.2

0.01

0.03

0.05

0.07

(^1^H) Squared Gradient Amplitude / (T^2^ m^-2^)

**Figure S32: A)** 400 MHz ^1^H-DOSY plots and **B)** corresponding signal fitting plots for a mixture of ethanol, propanol, and butanol in D_2_O, focusing on the spectral region from 3.4 to 3.8 ppm, processed using reference deconvolution based on a combined Lorentzian window function of -0.5 Hz and Gaussian width of 0.5 Hz. In all cases, manual phasing for each increment and manual baseline correction were applied.

**Table S17:** 1H-DOSY (400 MHz) data for a mixture of propanol, butanol, and ethanol in D_2_O, processed using reference deconvolution based on a combined Lorentzian window function of -0.5 Hz and Gaussian width of 0.5 Hz. Chemical shifts correspond to the region from 3.4 to 3.8 ppm.

| **Compound Frequencya** | **Exp. Ampl.** | **Fit. Ampl.** | **Error** | **Diff. coef.b** | **Errorb** |
| --- | --- | --- | --- | --- | --- |
| 3.53852 | 0.13814 | 0.14100 | 0.00015 | 6.93581 | 0.01398 |
| Propanol 3.55511 | 0.28237 | 0.28849 | 0.00017 | 6.92524 | 0.00744 |
| 3.57189 | 0.13468 | 0.13750 | 0.00015 | 6.94359 | 0.01396 |
| 3.58695 | 0.13651 | 0.13914 | 0.00009 | 6.28486 | 0.00804 |
| Butanol 3.60354 | 0.28761 | 0.29332 | 0.00011 | 6.26418 | 0.00456 |
| 3.62012 | 0.14540 | 0.14827 | 0.00007 | 6.29425 | 0.00615 |
| 3.62489 | 0.13829 | 0.14168 | 0.00013 | 8.14669 | 0.01307 |
| Ethanol 3.64262 | 0.41436 | 0.42475 | 0.00031 | 8.11825 | 0.01017 |
| 3.66035 | 0.42311 | 0.43353 | 0.00040 | 8.12308 | 0.01299 |
| 3.67827 | 0.12396 | 0.12689 | 0.00018 | 8.14872 | 0.02038 |

a Chemical shift values in ppm.

b Diffusion coefficients are expressed in units of 10*^−^*^10^ m^2^ s^-1^.

## Reference Deconvolution Based on a Combined Lorentzian Window Function (0.5 Hz) and Gaussian (0.5 Hz)

**(A) (B)**

0 1.2

Original

Fitted Residual

1.0

4 0.8

D / 10-10 m2 s-1

0.6

Amplitude

8

0.4

12 0.2

0.0

16

3.8 3.7 3.6 3.5 3.4

-0.2

0.01

0.03

0.05

0.07

(^1^H) Squared Gradient Amplitude / (T^2^ m^-2^)

**Figure S33: A)** 400 MHz ^1^H-DOSY plots and **B)** corresponding signal fitting plots for a mixture of ethanol, propanol, and butanol in D_2_O, focusing on the spectral region from 3.4 to 3.8 ppm, processed using reference deconvolution based on a combined Lorentzian window function of 0.5 Hz and Gaussian width of 0.5 Hz. In all cases, manual phasing for each increment and manual baseline correction were applied.

**Table S18:** 1H-DOSY (400 MHz) data for a mixture of propanol, butanol, and ethanol in D_2_O, processed using reference deconvolution based on a combined Lorentzian window function of 0.5 Hz and Gaussian width of 0.5 Hz. Chemical shifts correspond to the region from 3.4 to 3.8 ppm.

| **Compound Frequencya** | **Exp. Ampl.** | **Fit. Ampl.** | **Error** | **Diff. coef.b** | **Errorb** |
| --- | --- | --- | --- | --- | --- |
| 3.53852 | 0.20807 | 0.21267 | 0.00009 | 6.91311 | 0.00535 |
| Propanol 3.55511 | 0.41188 | 0.42095 | 0.00018 | 6.93290 | 0.00544 |
| 3.57189 | 0.20614 | 0.21068 | 0.00009 | 6.92082 | 0.00572 |
| 3.58695 | 0.27959 | 0.28518 | 0.00012 | 6.28874 | 0.00504 |
| Butanol 3.60354 | 0.55320 | 0.56427 | 0.00021 | 6.29694 | 0.00466 |
| 3.62012 | 0.28635 | 0.29206 | 0.00015 | 6.36445 | 0.00633 |
| 3.62489 | 0.16545 | 0.16950 | 0.00014 | 7.94746 | 0.01136 |
| Ethanol 3.64262 | 0.44466 | 0.45598 | 0.00028 | 8.13879 | 0.00856 |
| 3.66035 | 0.44790 | 0.45910 | 0.00038 | 8.15107 | 0.01163 |
| 3.67827 | 0.15033 | 0.15410 | 0.00013 | 8.16855 | 0.01236 |

a Chemical shift values in ppm.

b Diffusion coefficients are expressed in units of 10*^−^*^10^ m^2^ s^-1^.

# Processing with a Gaussian Window (1 Hz) - Global and Individual Phases Series

**(A)**

DOSY - Global Phase

**(B)**

DOSY - Individual Phase

0

4

D / 10-10 m2 s-1

8

12

16

3.8 3.7 3.6 3.5 3.4

(^1^H) / ppm

0

4

D / 10-10 m2 s-1

8

12

16

3.8 3.7 3.6 3.5 3.4

(^1^H) / ppm

**(C)** Global Phase **(D)** Individual Phase

8 8

4 4

0 0

Phase Change / Degrees

Phase Change / Degrees

-4 -4

-8

2 4 6

8 10 12 14 16

Increment Number

-8

2 4 6

8 10 12 14 16

Increment Number

**Figure S34:** DOSY plots for a mixture of ethanol, propanol, and butanol in D_2_O, focusing on the spectral region between 3.4 and 3.8 ppm. The plots were processed using **A)** a global phase correction and **B)** an individual phase correction. **C)** and **D)** show the phase variation (in degrees) across the 16 increments of the same DOSY experiment when **C)** a single global phase correction is applied to all increments simultaneously, and when **D)** manual phase correction is applied individually to each increment.

## Global Phase Correction

**(A) (B)**

0 1.2

Original

Fitted Residual

1.0

4 0.8

D / 10-10 m2 s-1

0.6

Amplitude

8

0.4

12 0.2

0.0

16

3.8 3.7 3.6 3.5 3.4

-0.2

0.01

0.03

0.05

0.07

(^1^H) Squared Gradient Amplitude / (T^2^ m^-2^)

**Figure S35: A)** 400 MHz ^1^H-DOSY plots and **B)** corresponding signal fitting plots for a mixture of ethanol, propanol, and butanol in D_2_O, focusing on the spectral region from 3.4 to 3.8 ppm, processed with a Gaussian window function of 1 Hz combined with manual and global phasing across all increments.

**Table S19:** 1H-DOSY (400 MHz) data for a mixture of propanol, butanol, and ethanol in D_2_O, processed with a Gaussian window function of 1 Hz combined with manual and global phasing across all increments. Chemical shifts correspond to the region from 3.4 to 3.8 ppm.

| **Compound Frequencya** | **Exp. Ampl.** | **Fit. Ampl.** | **Error** | **Diff. coef.b** | **Errorb** |
| --- | --- | --- | --- | --- | --- |
| 3.53867 | 0.22209 | 0.22709 | 0.00050 | 6.90780 | 0.02847 |
| Propanol 3.55545 | 0.43421 | 0.44379 | 0.00112 | 6.94215 | 0.03270 |
| 3.57213 | 0.21802 | 0.22280 | 0.00054 | 6.91634 | 0.03153 |
| 3.58709 | 0.31128 | 0.31725 | 0.00068 | 6.30220 | 0.02648 |
| Butanol 3.60378 | 0.61281 | 0.62438 | 0.00142 | 6.32195 | 0.02803 |
| 3.62046 | 0.31569 | 0.32196 | 0.00066 | 6.36634 | 0.02524 |
| 3.62494 | 0.16899 | 0.17275 | 0.00046 | 8.20918 | 0.03774 |
| Ethanol 3.64286 | 0.45310 | 0.46432 | 0.00116 | 8.19409 | 0.03533 |
| 3.66069 | 0.45533 | 0.46640 | 0.00122 | 8.20305 | 0.03721 |
| 3.67851 | 0.15571 | 0.15943 | 0.00042 | 8.24216 | 0.03775 |

a Chemical shift values in ppm.

b Diffusion coefficients are expressed in units of 10*^−^*^10^ m^2^ s^-1^.

## Individual Phase Correction

**(A) (B)**

0 1.2

Original

Fitted Residual

1.0

4 0.8

D / 10-10 m2 s-1

Amplitude

0.6

8

0.4

12 0.2

0.0

16

3.8 3.7 3.6 3.5 3.4

-0.2

0.01

0.03

0.05

0.07

(^1^H) Squared Gradient Amplitude / (T^2^ m^-2^)

**Figure S36: A)** 400 MHz ^1^H-DOSY plots and **B)** corresponding signal fitting plots for a mixture of ethanol, propanol, and butanol in D_2_O, focusing on the spectral region from 3.4 to 3.8 ppm, processed with a Gaussian window function of 1 Hz combined with manual and individual phasing for each increment.

**Table S20:** 1H-DOSY (400 MHz) data for a mixture of propanol, butanol, and ethanol in D_2_O, processed with a Gaussian window function of 1 Hz combined with manual and individual phasing for each increment. Chemical shifts correspond to the region from 3.4 to 3.8 ppm.

| **Compound Frequencya** | **Exp. Ampl.** | **Fit. Ampl.** | **Error** | **Diff. coef.b** | **Errorb** |
| --- | --- | --- | --- | --- | --- |
| 3.53867 | 0.22293 | 0.22781 | 0.00049 | 6.94250 | 0.02804 |
| Propanol 3.55535 | 0.43572 | 0.44473 | 0.00128 | 6.97693 | 0.03733 |
| 3.57213 | 0.21860 | 0.22331 | 0.00053 | 6.94223 | 0.03076 |
| 3.58709 | 0.31154 | 0.31747 | 0.00067 | 6.31116 | 0.02617 |
| Butanol 3.60378 | 0.61252 | 0.62415 | 0.00141 | 6.31950 | 0.02782 |
| 3.62046 | 0.31553 | 0.32179 | 0.00067 | 6.36090 | 0.02561 |
| 3.62494 | 0.16719 | 0.17110 | 0.00046 | 8.10166 | 0.03814 |
| Ethanol 3.64286 | 0.45204 | 0.46341 | 0.00116 | 8.17399 | 0.03551 |
| 3.66069 | 0.45407 | 0.46534 | 0.00122 | 8.17960 | 0.03717 |
| 3.67851 | 0.15450 | 0.15839 | 0.00042 | 8.17270 | 0.03791 |

a Chemical shift values in ppm.

b Diffusion coefficients are expressed in units of 10*^−^*^10^ m^2^ s^-1^.

# Processing with a Gaussian Window (1 Hz) - Baseline Order Series

## Automatic Baseline Correction (order 0)

**(A) (B)**

0 1.2

Original

Fitted Residual

1.0

4 0.8

D / 10-10 m2 s-1

0.6

Amplitude

8

0.4

12 0.2

0.0

16

3.8 3.7 3.6 3.5 3.4

-0.2

0.01

0.03

0.05

0.07

(^1^H) Squared Gradient Amplitude / (T^2^ m^-2^)

**Figure S37: A)** 400 MHz ^1^H-DOSY plots and **B)** corresponding signal fitting plots for a mixture of ethanol, propanol, and butanol in D_2_O, focusing on the spectral region from 3.4 to 3.8 ppm, processed with a Gaussian window function of 1 Hz. In all cases, manual phasing was performed for each increment, followed by automatic baseline correction (order 0).

**Table S21:** 1H-DOSY (400 MHz) data for a mixture of propanol, butanol, and ethanol in D_2_O, processed with a Gaussian window function of 1 Hz, followed by automatic baseline correction (order 0). Chemical shifts correspond to the region from 3.4 to 3.8 ppm.

| **Compound Frequencya** | **Exp. Ampl.** | **Fit. Ampl.** | **Error** | **Diff. coef.b** | **Errorb** |
| --- | --- | --- | --- | --- | --- |
| 3.53871 | 0.22162 | 0.22679 | 0.00082 | 6.90641 | 0.04675 |
| Propanol 3.55530 | 0.43579 | 0.44482 | 0.00125 | 6.97174 | 0.03639 |
| 3.57208 | 0.21868 | 0.22340 | 0.00053 | 6.93394 | 0.03051 |
| 3.58714 | 0.31015 | 0.31649 | 0.00092 | 6.28913 | 0.03574 |
| Butanol 3.60373 | 0.61250 | 0.62416 | 0.00138 | 6.32011 | 0.02733 |
| 3.62050 | 0.31378 | 0.32044 | 0.00107 | 6.34780 | 0.04148 |
| 3.62489 | 0.16702 | 0.17096 | 0.00044 | 8.15116 | 0.03655 |
| Ethanol 3.64281 | 0.45194 | 0.46333 | 0.00115 | 8.18123 | 0.03511 |
| 3.66073 | 0.45096 | 0.46270 | 0.00173 | 8.15282 | 0.05295 |
| 3.67846 | 0.15439 | 0.15828 | 0.00041 | 8.19965 | 0.03669 |

a Chemical shift values in ppm.

b Diffusion coefficients are expressed in units of 10*^−^*^10^ m^2^ s^-1^.

## Automatic Baseline Correction (order 1)

**(A) (B)**

0 1.2

Original

Fitted Residual

1.0

4 0.8

D / 10-10 m2 s-1

0.6

Amplitude

8

0.4

12 0.2

0.0

16

3.8 3.7 3.6 3.5 3.4

-0.2

0.01

0.03

0.05

0.07

(^1^H) Squared Gradient Amplitude / (T^2^ m^-2^)

**Figure S38: A)** 400 MHz ^1^H-DOSY plots and **B)** corresponding signal fitting plots for a mixture of ethanol, propanol, and butanol in D_2_O, focusing on the spectral region from 3.4 to 3.8 ppm, processed with a Gaussian window function of 1 Hz. In all cases, manual phasing was performed for each increment, followed by automatic baseline correction (order 1).

**Table S22:** 1H-DOSY (400 MHz) data for a mixture of propanol, butanol, and ethanol in D_2_O, processed with a Gaussian window function of 1 Hz, followed by automatic baseline correction (order 1). Chemical shifts correspond to the region from 3.4 to 3.8 ppm.

| **Compound Frequencya** | **Exp. Ampl.** | **Fit. Ampl.** | **Error** | **Diff. coef.b** | **Errorb** |
| --- | --- | --- | --- | --- | --- |
| 3.53871 | 0.22156 | 0.22672 | 0.00082 | 6.90644 | 0.04675 |
| Propanol 3.55530 | 0.43576 | 0.44478 | 0.00125 | 6.97177 | 0.03639 |
| 3.57208 | 0.21861 | 0.22333 | 0.00053 | 6.93398 | 0.03051 |
| 3.58714 | 0.31009 | 0.31643 | 0.00092 | 6.28896 | 0.03574 |
| Butanol 3.60373 | 0.61249 | 0.62415 | 0.00138 | 6.32004 | 0.02733 |
| 3.62050 | 0.31373 | 0.32039 | 0.00107 | 6.34766 | 0.04148 |
| 3.62489 | 0.16695 | 0.17089 | 0.00044 | 8.15202 | 0.03652 |
| Ethanol 3.64281 | 0.45191 | 0.46330 | 0.00115 | 8.18156 | 0.03511 |
| 3.66073 | 0.45093 | 0.46267 | 0.00173 | 8.15314 | 0.05295 |
| 3.67846 | 0.15432 | 0.15820 | 0.00041 | 8.20063 | 0.03667 |

a Chemical shift values in ppm.

b Diffusion coefficients are expressed in units of 10*^−^*^10^ m^2^ s^-1^.

## Automatic Baseline Correction (order 3)

**(A) (B)**

0 1.2

Original

Fitted Residual

1.0

4 0.8

D / 10-10 m2 s-1

0.6

Amplitude

8

0.4

12 0.2

0.0

16

3.8 3.7 3.6 3.5 3.4

-0.2

0.01

0.03

0.05

0.07

(^1^H) Squared Gradient Amplitude / (T^2^ m^-2^)

**Figure S39: A)** 400 MHz ^1^H-DOSY plots and **B)** corresponding signal fitting plots for a mixture of ethanol, propanol, and butanol in D_2_O, focusing on the spectral region from 3.4 to 3.8 ppm, processed with a Gaussian window function of 1 Hz. In all cases, manual phasing was performed for each increment, followed by automatic baseline correction (order 3).

**Table S23:** 1H-DOSY (400 MHz) data for a mixture of propanol, butanol, and ethanol in D_2_O, processed with a Gaussian window function of 1 Hz, followed by automatic baseline correction (order 3). Chemical shifts correspond to the region from 3.4 to 3.8 ppm.

| **Compound Frequencya** | **Exp. Ampl.** | **Fit. Ampl.** | **Error** | **Diff. coef.b** | **Errorb** |
| --- | --- | --- | --- | --- | --- |
| 3.53871 | 0.22153 | 0.22668 | 0.00082 | 6.90816 | 0.04682 |
| Propanol 3.55530 | 0.43573 | 0.44475 | 0.00125 | 6.97267 | 0.03639 |
| 3.57208 | 0.21858 | 0.22329 | 0.00053 | 6.93575 | 0.03052 |
| 3.58714 | 0.31007 | 0.31640 | 0.00092 | 6.28999 | 0.03578 |
| Butanol 3.60373 | 0.61247 | 0.62412 | 0.00138 | 6.32056 | 0.02734 |
| 3.62050 | 0.31370 | 0.32035 | 0.00108 | 6.34868 | 0.04152 |
| 3.62489 | 0.16692 | 0.17085 | 0.00044 | 8.15515 | 0.03658 |
| Ethanol 3.64281 | 0.45189 | 0.46327 | 0.00115 | 8.18272 | 0.03512 |
| 3.66073 | 0.45091 | 0.46264 | 0.00173 | 8.15428 | 0.05299 |
| 3.67846 | 0.15429 | 0.15817 | 0.00041 | 8.20402 | 0.03669 |

a Chemical shift values in ppm.

b Diffusion coefficients are expressed in units of 10*^−^*^10^ m^2^ s^-1^.

## Automatic Baseline Correction (order 5)

**(A) (B)**

0 1.2

Original

Fitted Residual

1.0

4 0.8

D / 10-10 m2 s-1

0.6

Amplitude

8

0.4

12 0.2

0.0

16

3.8 3.7 3.6 3.5 3.4

-0.2

0.01

0.03

0.05

0.07

(^1^H) Squared Gradient Amplitude / (T^2^ m^-2^)

**Figure S40: A)** 400 MHz ^1^H-DOSY plots and **B)** corresponding signal fitting plots for a mixture of ethanol, propanol, and butanol in D_2_O, focusing on the spectral region from 3.4 to 3.8 ppm, processed with a Gaussian window function of 1 Hz. In all cases, manual phasing was performed for each increment, followed by automatic baseline correction (order 5).

**Table S24:** 1H-DOSY (400 MHz) data for a mixture of propanol, butanol, and ethanol in D_2_O, processed with a Gaussian window function of 1 Hz, followed by automatic baseline correction (order 5). Chemical shifts correspond to the region from 3.4 to 3.8 ppm.

| **Compound Frequencya** | **Exp. Ampl.** | **Fit. Ampl.** | **Error** | **Diff. coef.b** | **Errorb** |
| --- | --- | --- | --- | --- | --- |
| 3.53871 | 0.22157 | 0.22673 | 0.00082 | 6.90797 | 0.04683 |
| Propanol 3.55530 | 0.43579 | 0.44481 | 0.00125 | 6.97256 | 0.03637 |
| 3.57208 | 0.21862 | 0.22333 | 0.00053 | 6.93552 | 0.03049 |
| 3.58714 | 0.31011 | 0.31645 | 0.00092 | 6.28990 | 0.03579 |
| Butanol 3.60373 | 0.61254 | 0.62419 | 0.00138 | 6.32051 | 0.02733 |
| 3.62050 | 0.31375 | 0.32040 | 0.00108 | 6.34857 | 0.04153 |
| 3.62489 | 0.16696 | 0.17089 | 0.00044 | 8.15445 | 0.03656 |
| Ethanol 3.64281 | 0.45195 | 0.46333 | 0.00115 | 8.18245 | 0.03511 |
| 3.66073 | 0.45096 | 0.46270 | 0.00173 | 8.15400 | 0.05299 |
| 3.67846 | 0.15433 | 0.15821 | 0.00041 | 8.20316 | 0.03666 |

a Chemical shift values in ppm.

b Diffusion coefficients are expressed in units of 10*^−^*^10^ m^2^ s^-1^.

## Automatic Baseline Correction (order 8)

**(A) (B)**

0 1.2

Original

Fitted Residual

1.0

4 0.8

D / 10-10 m2 s-1

0.6

Amplitude

8

0.4

12 0.2

0.0

16

3.8 3.7 3.6 3.5 3.4

-0.2

0.01

0.03

0.05

0.07

(^1^H) Squared Gradient Amplitude / (T^2^ m^-2^)

**Figure S41: A)** 400 MHz ^1^H-DOSY plots and **B)** corresponding signal fitting plots for a mixture of ethanol, propanol, and butanol in D_2_O, focusing on the spectral region from 3.4 to 3.8 ppm, processed with a Gaussian window function of 1 Hz. In all cases, manual phasing was performed for each increment, followed by automatic baseline correction (order 8).

**Table S25:** 1H-DOSY (400 MHz) data for a mixture of propanol, butanol, and ethanol in D_2_O, processed with a Gaussian window function of 1 Hz, followed by automatic baseline correction (order 8). Chemical shifts correspond to the region from 3.4 to 3.8 ppm.

| **Compound Frequencya** | **Exp. Ampl.** | **Fit. Ampl.** | **Error** | **Diff. coef.b** | **Errorb** |
| --- | --- | --- | --- | --- | --- |
| 3.53871 | 0.22160 | 0.22676 | 0.00082 | 6.90772 | 0.04683 |
| Propanol 3.55530 | 0.43584 | 0.44486 | 0.00125 | 6.97242 | 0.03637 |
| 3.57208 | 0.21865 | 0.22337 | 0.00053 | 6.93522 | 0.03048 |
| 3.58714 | 0.31015 | 0.31649 | 0.00092 | 6.28972 | 0.03579 |
| Butanol 3.60373 | 0.61260 | 0.62425 | 0.00138 | 6.32041 | 0.02733 |
| 3.62050 | 0.31378 | 0.32044 | 0.00108 | 6.34835 | 0.04153 |
| 3.62489 | 0.16698 | 0.17092 | 0.00044 | 8.15379 | 0.03654 |
| Ethanol 3.64281 | 0.45199 | 0.46337 | 0.00115 | 8.18219 | 0.03511 |
| 3.66073 | 0.45100 | 0.46275 | 0.00173 | 8.15374 | 0.05299 |
| 3.67846 | 0.15435 | 0.15823 | 0.00041 | 8.20234 | 0.03665 |

a Chemical shift values in ppm.

b Diffusion coefficients are expressed in units of 10*^−^*^10^ m^2^ s^-1^.

## Manual Baseline Correction (order 0)

**(A) (B)**

0 1.2

Original

Fitted Residual

1.0

4 0.8

D / 10-10 m2 s-1

0.6

Amplitude

8

0.4

12 0.2

0.0

16

3.8 3.7 3.6 3.5 3.4

-0.2

0.01

0.03

0.05

0.07

(^1^H) Squared Gradient Amplitude / (T^2^ m^-2^)

**Figure S42: A)** 400 MHz ^1^H-DOSY plots and **B)** corresponding signal fitting plots for a mixture of ethanol, propanol, and butanol in D_2_O, focusing on the spectral region from 3.4 to 3.8 ppm, processed with a Gaussian window function of 1 Hz. In all cases, manual phasing was performed for each increment, followed by manual baseline correction (order 0).

**Table S26:** 1H-DOSY (400 MHz) data for a mixture of propanol, butanol, and ethanol in D_2_O, processed with a Gaussian window function of 1 Hz, followed by manual baseline correction (order 0). Chemical shifts correspond to the region from 3.4 to 3.8 ppm.

| **Compound Frequencya** | **Exp. Ampl.** | **Fit. Ampl.** | **Error** | **Diff. coef.b** | **Errorb** |
| --- | --- | --- | --- | --- | --- |
| 3.53871 | 0.22163 | 0.22679 | 0.00082 | 6.90666 | 0.04676 |
| Propanol 3.55530 | 0.43580 | 0.44482 | 0.00125 | 6.97187 | 0.03638 |
| 3.57208 | 0.21868 | 0.22340 | 0.00053 | 6.93419 | 0.03049 |
| 3.58714 | 0.31015 | 0.31649 | 0.00092 | 6.28930 | 0.03575 |
| Butanol 3.60373 | 0.61250 | 0.62416 | 0.00138 | 6.32020 | 0.02733 |
| 3.62050 | 0.31378 | 0.32044 | 0.00107 | 6.34796 | 0.04149 |
| 3.62489 | 0.16702 | 0.17097 | 0.00044 | 8.15152 | 0.03654 |
| Ethanol 3.64281 | 0.45195 | 0.46333 | 0.00115 | 8.18136 | 0.03511 |
| 3.66073 | 0.45096 | 0.46270 | 0.00173 | 8.15295 | 0.05296 |
| 3.67846 | 0.15440 | 0.15828 | 0.00041 | 8.20004 | 0.03667 |

a Chemical shift values in ppm.

b Diffusion coefficients are expressed in units of 10*^−^*^10^ m^2^ s^-1^.

## Manual Baseline Correction (order 1)

**(A) (B)**

0 1.2

Original

Fitted Residual

1.0

4 0.8

D / 10-10 m2 s-1

0.6

Amplitude

8

0.4

12 0.2

0.0

16

3.8 3.7 3.6 3.5 3.4

-0.2

0.01

0.03

0.05

0.07

(^1^H) Squared Gradient Amplitude / (T^2^ m^-2^)

**Figure S43: A)** 400 MHz ^1^H-DOSY plots and **B)** corresponding signal fitting plots for a mixture of ethanol, propanol, and butanol in D_2_O, focusing on the spectral region from 3.4 to 3.8 ppm, processed with a Gaussian window function of 1 Hz. In all cases, manual phasing was performed for each increment, followed by manual baseline correction (order 1).

**Table S27:** 1H-DOSY (400 MHz) data for a mixture of propanol, butanol, and ethanol in D_2_O, processed with a Gaussian window function of 1 Hz, followed by manual baseline correction (order 1). Chemical shifts correspond to the region from 3.4 to 3.8 ppm.

| **Compound Frequencya** | **Exp. Ampl.** | **Fit. Ampl.** | **Error** | **Diff. coef.b** | **Errorb** |
| --- | --- | --- | --- | --- | --- |
| 3.53871 | 0.22162 | 0.22679 | 0.00082 | 6.90641 | 0.04675 |
| Propanol 3.55530 | 0.43579 | 0.44482 | 0.00125 | 6.97174 | 0.03639 |
| 3.57208 | 0.21868 | 0.22340 | 0.00053 | 6.93394 | 0.03051 |
| 3.58714 | 0.31015 | 0.31649 | 0.00092 | 6.28913 | 0.03574 |
| Butanol 3.60373 | 0.61250 | 0.62416 | 0.00138 | 6.32011 | 0.02733 |
| 3.62050 | 0.31378 | 0.32044 | 0.00107 | 6.34780 | 0.04148 |
| 3.62489 | 0.16702 | 0.17096 | 0.00044 | 8.15116 | 0.03655 |
| Ethanol 3.64281 | 0.45194 | 0.46333 | 0.00115 | 8.18123 | 0.03511 |
| 3.66073 | 0.45096 | 0.46270 | 0.00173 | 8.15282 | 0.05295 |
| 3.67846 | 0.15439 | 0.15828 | 0.00041 | 8.19965 | 0.03669 |

a Chemical shift values in ppm.

b Diffusion coefficients are expressed in units of 10*^−^*^10^ m^2^ s^-1^.

## Manual Baseline Correction (order 3)

**(A) (B)**

0 1.2

Original

Fitted Residual

1.0

4 0.8

D / 10-10 m2 s-1

0.6

Amplitude

8

0.4

12 0.2

0.0

16

3.8 3.7 3.6 3.5 3.4

-0.2

0.01

0.03

0.05

0.07

(^1^H) Squared Gradient Amplitude / (T^2^ m^-2^)

**Figure S44: A)** 400 MHz ^1^H-DOSY plots and **B)** corresponding signal fitting plots for a mixture of ethanol, propanol, and butanol in D_2_O, focusing on the spectral region from 3.4 to 3.8 ppm, processed with a Gaussian window function of 1 Hz. In all cases, manual phasing was performed for each increment, followed by manual baseline correction (order 3).

**Table S28:** 1H-DOSY (400 MHz) data for a mixture of propanol, butanol, and ethanol in D_2_O, processed with a Gaussian window function of 1 Hz, followed by manual baseline correction (order 3). Chemical shifts correspond to the region from 3.4 to 3.8 ppm.

| **Compound Frequencya** | **Exp. Ampl.** | **Fit. Ampl.** | **Error** | **Diff. coef.b** | **Errorb** |
| --- | --- | --- | --- | --- | --- |
| 3.53871 | 0.22162 | 0.22679 | 0.00082 | 6.90596 | 0.04674 |
| Propanol 3.55530 | 0.43579 | 0.44482 | 0.00125 | 6.97151 | 0.03638 |
| 3.57208 | 0.21868 | 0.22339 | 0.00053 | 6.93349 | 0.03049 |
| 3.58714 | 0.31015 | 0.31649 | 0.00092 | 6.28884 | 0.03574 |
| Butanol 3.60373 | 0.61250 | 0.62416 | 0.00138 | 6.31997 | 0.02733 |
| 3.62050 | 0.31378 | 0.32044 | 0.00107 | 6.34752 | 0.04148 |
| 3.62489 | 0.16702 | 0.17096 | 0.00044 | 8.15051 | 0.03653 |
| Ethanol 3.64281 | 0.45195 | 0.46333 | 0.00115 | 8.18099 | 0.03511 |
| 3.66073 | 0.45096 | 0.46270 | 0.00173 | 8.15258 | 0.05295 |
| 3.67846 | 0.15439 | 0.15828 | 0.00041 | 8.19898 | 0.03667 |

a Chemical shift values in ppm.

b Diffusion coefficients are expressed in units of 10*^−^*^10^ m^2^ s^-1^.

## Manual Baseline Correction (order 5)

**(A) (B)**

0 1.2

Original

Fitted Residual

1.0

4 0.8

D / 10-10 m2 s-1

0.6

Amplitude

8

0.4

12 0.2

0.0

16

3.8 3.7 3.6 3.5 3.4

-0.2

0.01

0.03

0.05

0.07

(^1^H) Squared Gradient Amplitude / (T^2^ m^-2^)

**Figure S45: A)** 400 MHz ^1^H-DOSY plots and **B)** corresponding signal fitting plots for a mixture of ethanol, propanol, and butanol in D_2_O, focusing on the spectral region from 3.4 to 3.8 ppm, processed with a Gaussian window function of 1 Hz. In all cases, manual phasing was performed for each increment, followed by manual baseline correction (order 5).

**Table S29:** 1H-DOSY (400 MHz) data for a mixture of propanol, butanol, and ethanol in D_2_O, processed with a Gaussian window function of 1 Hz, followed by manual baseline correction (order 5). Chemical shifts correspond to the region from 3.4 to 3.8 ppm.

| **Compound Frequencya** | **Exp. Ampl.** | **Fit. Ampl.** | **Error** | **Diff. coef.b** | **Errorb** |
| --- | --- | --- | --- | --- | --- |
| 3.53871 | 0.22162 | 0.22679 | 0.00082 | 6.90486 | 0.04683 |
| Propanol 3.55530 | 0.43580 | 0.44483 | 0.00125 | 6.97095 | 0.03639 |
| 3.57208 | 0.21868 | 0.22340 | 0.00053 | 6.93238 | 0.03054 |
| 3.58714 | 0.31015 | 0.31649 | 0.00092 | 6.28816 | 0.03579 |
| Butanol 3.60373 | 0.61250 | 0.62417 | 0.00138 | 6.31962 | 0.02734 |
| 3.62050 | 0.31378 | 0.32045 | 0.00108 | 6.34685 | 0.04153 |
| 3.62489 | 0.16702 | 0.17096 | 0.00044 | 8.14871 | 0.03658 |
| Ethanol 3.64281 | 0.45195 | 0.46333 | 0.00115 | 8.18034 | 0.03515 |
| 3.66073 | 0.45096 | 0.46271 | 0.00173 | 8.15193 | 0.05299 |
| 3.67846 | 0.15439 | 0.15828 | 0.00041 | 8.19708 | 0.03677 |

a Chemical shift values in ppm.

b Diffusion coefficients are expressed in units of 10*^−^*^10^ m^2^ s^-1^.

## Manual Baseline Correction (order 8)

**(A) (B)**

0 1.2

Original

Fitted Residual

1.0

4 0.8

D / 10-10 m2 s-1

0.6

Amplitude

8

0.4

12 0.2

0.0

16

3.8 3.7 3.6 3.5 3.4

-0.2

0.01

0.03

0.05

0.07

(^1^H) Squared Gradient Amplitude / (T^2^ m^-2^)

**Figure S46: A)** 400 MHz ^1^H-DOSY plots and **B)** corresponding signal fitting plots for a mixture of ethanol, propanol, and butanol in D_2_O, focusing on the spectral region from 3.4 to 3.8 ppm, processed with a Gaussian window function of 1 Hz. In all cases, manual phasing was performed for each increment, followed by manual baseline correction (order 8).

**Table S30:** 1H-DOSY (400 MHz) data for a mixture of propanol, butanol, and ethanol in D_2_O, processed with a Gaussian window function of 1 Hz, followed by manual baseline correction (order 8). Chemical shifts correspond to the region from 3.4 to 3.8 ppm.

| **Compound Frequencya** | **Exp. Ampl.** | **Fit. Ampl.** | **Error** | **Diff. coef.b** | **Errorb** |
| --- | --- | --- | --- | --- | --- |
| 3.53871 | 0.22162 | 0.22680 | 0.00082 | 6.92705 | 0.04677 |
| Propanol 3.55530 | 0.43580 | 0.44484 | 0.00124 | 6.98230 | 0.03626 |
| 3.57208 | 0.21868 | 0.22340 | 0.00052 | 6.95459 | 0.03026 |
| 3.58714 | 0.31015 | 0.31649 | 0.00092 | 6.30201 | 0.03573 |
| Butanol 3.60373 | 0.61252 | 0.62417 | 0.00138 | 6.32662 | 0.02728 |
| 3.62050 | 0.31379 | 0.32044 | 0.00107 | 6.36036 | 0.04149 |
| 3.62489 | 0.16702 | 0.17099 | 0.00043 | 8.18286 | 0.03576 |
| Ethanol 3.64281 | 0.45196 | 0.46337 | 0.00114 | 8.19280 | 0.03488 |
| 3.66073 | 0.45097 | 0.46274 | 0.00173 | 8.16419 | 0.05292 |
| 3.67846 | 0.15439 | 0.15831 | 0.00040 | 8.23285 | 0.03599 |

a Chemical shift values in ppm.

b Diffusion coefficients are expressed in units of 10*^−^*^10^ m^2^ s^-1^.

# Absolute Signal-to-Noise (SNR) Ratio Values

**Table S31:** Signal-to-noise ratios (SNRs) of selected frequencies for propanol, butanol, ethanol, and TSP under various processing methods. Values were extracted from signals in the region of 3.4–3.8 ppm: approximately 3.538 ppm (propanol), 3.587 ppm (butanol), 3.678 ppm (ethanol), and 0.00 ppm (TSP).

| **Processing Methodsa** | **Propanol** | **Butanol** | **Ethanol** | **TSP** |
| --- | --- | --- | --- | --- |
| Only FT | 3763.60 | 4601.16 | 2973.08 | 7073.27 |
| lw = 1 | 1020.72 | 1447.83 | 712.42 | 1569.09 |
| lw = 2 | 490.50 | 728.61 | 335.70 | 692.46 |
| gw = 1 | 2070.76 | 2899.22 | 1445.10 | 3260.78 |
| gw = 2 | 1175.77 | 1707.32 | 787.51 | 1770.34 |
| lw = 1 and gw = 1 | 819.41 | 1191.37 | 557.68 | 1218.48 |
| RD: lw = 1 | 1212.35 | 1672.46 | 865.52 | 1934.41 |
| RD: gw = 1 | 2664.24 | 3660.61 | 1887.87 | 4272.31 |
| RD: lw = *−*0*.*3 and gw = 1 | 4129.12 | 5547.89 | 2972.64 | 6774.98 |
| RD: lw = 1 and gw = 1 | 918.40 | 1323.25 | 628.28 | 1384.23 |

a Lorentzian (lw) and Gaussian (gw) window functions are specified in Hz. "Only FT" refers to processing without any window function applied, while RD indicates processing with reference deconvolution.

**Table S32:** Signal-to-noise ratios (SNRs) of selected frequencies for propanol, butanol, ethanol, and TSP under various processing methods. Values were extracted from signals in the region of 3.4–3.8 ppm: approximately 3.538 ppm (propanol), 3.587 ppm (butanol), 3.678 ppm (ethanol), and 0.00 ppm (TSP). All processing parameters were selected based on the experimental line-width at half height of the TSP signal (0.5 Hz).

| **Processing Methodsa** | **Propanol** | **Butanol** | **Ethanol** | **TSP** |
| --- | --- | --- | --- | --- |
| Only FT | 3763.60 | 4601.16 | 2973.08 | 7073.27 |
| lw = 0.5 | 2469.77 | 3401.16 | 2701.06 | 3083.60 |
| gw = 0.5 | 4229.80 | 5710.57 | 4670.49 | 5422.38 |
| lw = *−*0*.*5 and gw = 0.5 | 9864.82 | 11996.85 | 11876.54 | 14245.97 |
| lw = 0.5 and gw = 0.5 | 2167.81 | 3037.93 | 2307.24 | 2599.77 |
| RD: lw = 0.5 | 3233.97 | 4126.10 | 3881.21 | 4472.00 |
| RD: gw = 0.5 | 6368.65 | 7877.13 | 7585.92 | 8803.53 |
| RD: lw = *−*0*.*5 and gw = 0.5 | 6664.71 | 6837.80 | 9900.49 | 11807.13 |
| RD: lw = 0.5 and gw = 0.5 | 2784.38 | 3770.87 | 3054.38 | 3463.95 |

a Lorentzian (lw) and Gaussian (gw) window functions are specified in Hz. "Only FT" refers to processing without any window function applied, while RD indicates processing with reference deconvolution.

# Average Diffusion Coefficients and Relative Differences

**Table S33:** Average diffusion coefficients and their corresponding propagated errors (both expressed in *×*10*^−^*^10^ m^2^ s^-1^) for butanol, propanol, ethanol, and TSP under various processing conditions. The signal fitting was performed in the spectral region from 3.4 to 3.8 ppm for the alcohols and at 0.0 ppm

for TSP.

|  | Diff. | Error | % Error |  | Diff. | Error | % Error |  | Diff. | Error | % Error |  | Diff. | Error | % Error |  |
| --- | --- | --- | --- | --- | --- | --- | --- | --- | --- | --- | --- | --- | --- | --- | --- | --- |
| Only FT | 6.35 | 0.2 | 3.15 |  | 6.98 | 0.26 | 3.72 |  | 8.31 | 0.53 | 6.38 |  | 4.42 | 0.22 | 4.98 |  |
| lw = 1 | 6.36 | 0.05 | 0.79 |  | 6.90 | 0.06 | 0.87 |  | 8.14 | 0.09 | 1.11 |  | 4.32 | 0.04 | 0.93 |  |
| lw = 2 | 6.42 | 0.02 | 0.31 |  | 6.85 | 0.03 | 0.44 |  | 8.23 | 0.04 | 0.49 |  | 4.32 | 0.02 | 0.46 |  |
| gw = 1 | 6.32 | 0.06 | 0.95 |  | 6.92 | 0.07 | 1.01 |  | 8.22 | 0.09 | 1.09 |  | 4.32 | 0.04 | 0.93 |  |
| gw = 2 | 6.38 | 0.02 | 0.31 |  | 6.88 | 0.02 | 0.29 |  | 8.23 | 0.04 | 0.49 |  | 4.32 | 0.02 | 0.46 |  |
| lw = 1 and gw = 1 | 6.38 | 0.03 | 0.47 |  | 6.88 | 0.03 | 0.44 |  | 8.23 | 0.05 | 0.61 |  | 4.32 | 0.02 | 0.46 |  |
| RD: lw = 1 | 6.34 | 0.01 | 0.16 |  | 6.90 | 0.01 | 0.14 |  | 8.09 | 0.02 | 0.25 |  | 4.31 | 0.00 | 0.00 |  |
| RD: gw = 1 | 6.31 | 0.01 | 0.16 |  | 6.92 | 0.01 | 0.14 |  | 8.15 | 0.02 | 0.25 |  | 4.31 | 0.00 | 0.00 |  |
| RD: lw = -0.3 and gw = 1 | 6.30 | 0.01 | 0.16 |  | 6.92 | 0.01 | 0.14 |  | 8.17 | 0.02 | 0.24 |  | 4.32 | 0.00 | 0.00 |  |
| RD: lw = 1 and gw = 1 | 6.36 | 0.01 | 0.16 |  | 6.89 | 0.01 | 0.15 |  | 8.05 | 0.03 | 0.37 |  | 4.31 | 0.00 | 0.00 |  |

**Processing Methodsa Butanol Propanol Ethanol TSP**

a Lorentzian (lw) and Gaussian (gw) window functions are specified in Hz. "Only FT" refers to processing without any window function applied, while RD indicates processing with reference deconvolution.

**Table S34:** Average diffusion coefficients and their corresponding propagated errors (both expressed in *×*10*^−^*^10^ m^2^ s^-1^) for butanol, propanol, ethanol, and TSP under various processing conditions. All processing parameters were selected based on the experimental line-width at half height of the TSP

signal (0.5 Hz). The signal fitting was performed in the spectral region from 3.4 to 3.8 ppm for the alcohols and at 0.0 ppm for TSP.

|  | Diff. | Error | % Error |  | Diff. | Error | % Error |  | Diff. | Error | % Error |  | Diff. | Error | % Error |  |
| --- | --- | --- | --- | --- | --- | --- | --- | --- | --- | --- | --- | --- | --- | --- | --- | --- |
| Only FT | 6.35 | 0.20 | 3.15 |  | 6.98 | 0.26 | 3.72 |  | 8.31 | 0.53 | 6.38 |  | 4.42 | 0.22 | 4.98 |  |
| lw = 0.5 | 6.34 | 0.09 | 1.48 |  | 6.93 | 0.13 | 1.82 |  | 8.16 | 0.18 | 2.17 |  | 4.34 | 0.07 | 1.68 |  |
| gw = 0.5 | 6.35 | 0.10 | 1.58 |  | 6.94 | 0.15 | 2.21 |  | 8.21 | 0.20 | 2.49 |  | 4.34 | 0.08 | 1.94 |  |
| lw = -0.5 and gw = 0.5 | 6.34 | 0.22 | 3.48 |  | 6.98 | 0.29 | 4.09 |  | 8.31 | 0.46 | 5.54 |  | 4.40 | 0.19 | 4.24 |  |
| lw = 0.5 and gw = 0.5 | 6.33 | 0.07 | 1.08 |  | 6.94 | 0.08 | 1.12 |  | 8.14 | 0.10 | 1.28 |  | 4.32 | 0.04 | 1.02 |  |
| RD: lw = 0.5 | 6.31 | 0.01 | 0.15 |  | 6.93 | 0.01 | 0.18 |  | 8.11 | 0.02 | 0.29 |  | 4.31 | 0.00 | 0.09 |  |
| RD: gw = 0.5 | 6.30 | 0.01 | 0.15 |  | 6.93 | 0.01 | 0.18 |  | 8.13 | 0.02 | 0.27 |  | 4.31 | 0.00 | 0.09 |  |
| RD: lw = -0.5 and gw = 0.5 | 6.28 | 0.01 | 0.18 |  | 6.93 | 0.02 | 0.30 |  | 8.13 | 0.03 | 0.36 |  | 4.31 | 0.00 | 0.09 |  |
| RD: lw = 0.5 and gw = 0.5 | 6.32 | 0.01 | 0.15 |  | 6.92 | 0.01 | 0.14 |  | 8.10 | 0.02 | 0.27 |  | 4.31 | 0.00 | 0.09 |  |

**Processing Methodsa Butanol Propanol Ethanol TSP**

a Lorentzian (lw) and Gaussian (gw) window functions are specified in Hz. ”Only FT” refers to processing without any window function applied, while RD indicates processing with reference deconvolution.

**Table S35:** Average diffusion coefficients and their corresponding propagated errors (both expressed in *×*10*^−^*^10^ m^2^ s^-1^) for butanol, propanol, and ethanol under different phasing corrections. The signal fitting was performed in the spectral region from 3.4 to 3.8 ppm, processed with a Gaussian window

function of 1 Hz.

**Phasing Procedure Butanol Propanol Ethanol**

Diff. Error % Error Diff. Error % Error Diff. Error % Error

Global Correction 6.33 0.05 0.73 6.92 0.05 0.77 8.21 0.07 0.90

Individual Correction 6.33 0.05 0.73 6.95 0.06 0.80 8.16 0.07 0.91

**Table S36:** Average diffusion coefficients and their corresponding propagated errors (both expressed in *×*10*^−^*^10^ m^2^ s^-1^) for butanol, propanol, ethanol, and TSP under the processing conditions of a Gaussian window function of 1 Hz and various automatic baseline correction orders. The signal

fitting was performed in the spectral region from 3.4 to 3.8 ppm for the alcohols and at 0.0 ppm for TSP.

|  | Diff. | Error | % Error |  | Diff. | Error | % Error |  | Diff. | Error | % Error |  | Diff. | Error | % Error |  |
| --- | --- | --- | --- | --- | --- | --- | --- | --- | --- | --- | --- | --- | --- | --- | --- | --- |
| 0 | 6.32 | 0.06 | 0.97 |  | 6.94 | 0.07 | 0.96 |  | 8.17 | 0.08 | 1.00 |  | 4.32 | 0.03 | 0.79 |  |
| 1 | 6.32 | 0.06 | 0.97 |  | 6.94 | 0.07 | 0.96 |  | 8.17 | 0.08 | 1.00 |  | 4.32 | 0.03 | 0.79 |  |
| 3 | 6.32 | 0.06 | 0.97 |  | 6.94 | 0.07 | 0.96 |  | 8.17 | 0.08 | 1.00 |  | 4.31 | 0.03 | 0.79 |  |
| 5 | 6.32 | 0.06 | 0.97 |  | 6.94 | 0.07 | 0.96 |  | 8.17 | 0.08 | 1.00 |  | 4.31 | 0.03 | 0.79 |  |
| 8 | 6.32 | 0.06 | 0.97 |  | 6.94 | 0.07 | 0.96 |  | 8.17 | 0.08 | 1.00 |  | 4.32 | 0.03 | 0.79 |  |

**Automatic Baseline Order Butanol Propanol Ethanol TSP**

**Table S37:** Average diffusion coefficients and their corresponding propagated errors (both expressed in *×*10*^−^*^10^ m^2^ s^-1^) for butanol, propanol, ethanol, and TSP under the processing conditions of a Gaussian window function of 1 Hz and various manual baseline correction orders. The signal fitting

was performed in the spectral region from 3.4 to 3.8 ppm for the alcohols and at 0.0 ppm for TSP.

|  | Diff. | Error | % Error |  | Diff. | Error | % Error |  | Diff. | Error | % Error |  | Diff. | Error | % Error |  |
| --- | --- | --- | --- | --- | --- | --- | --- | --- | --- | --- | --- | --- | --- | --- | --- | --- |
| 0 | 6.32 | 0.06 | 0.97 |  | 6.94 | 0.07 | 0.96 |  | 8.17 | 0.08 | 1.00 |  | 4.32 | 0.03 | 0.79 |  |
| 1 | 6.32 | 0.06 | 0.97 |  | 6.94 | 0.07 | 0.96 |  | 8.17 | 0.08 | 1.00 |  | 4.32 | 0.03 | 0.79 |  |
| 3 | 6.32 | 0.06 | 0.97 |  | 6.94 | 0.07 | 0.96 |  | 8.17 | 0.08 | 1.00 |  | 4.32 | 0.03 | 0.79 |  |
| 5 | 6.32 | 0.06 | 0.97 |  | 6.94 | 0.07 | 0.96 |  | 8.17 | 0.08 | 1.00 |  | 4.32 | 0.03 | 0.79 |  |
| 8 | 6.33 | 0.06 | 0.97 |  | 6.95 | 0.07 | 0.96 |  | 8.19 | 0.08 | 0.99 |  | 4.32 | 0.03 | 0.79 |  |

**Manual Baseline Order Butanol Propanol Ethanol TSP**

**Table S38:** Average diffusion coefficient differences (∆*D* = *D*_alcohol_ *− D*_TSP_) and their associated propagated errors (both in *×*10*^−^*^10^ m^2^ s^-1^) for butanol, propanol, and ethanol under various processing conditions.

**Processing Methodsa Butanol Propanol Ethanol**

∆*D* Error ∆*D* Error ∆*D* Error

Only FT 1.93 0.30 2.56 0.34 3.89 0.57

lw = 1 2.04 0.06 2.58 0.07 3.82 0.10

lw = 2 2.10 0.03 2.53 0.03 3.92 0.05

gw = 1 2.00 0.07 2.60 0.08 3.90 0.09

gw = 2 2.07 0.03 2.56 0.03 3.91 0.04

lw = 1 and gw = 1 2.06 0.03 2.56 0.04 3.91 0.05

RD: lw = 1 2.03 0.01 2.59 0.01 3.78 0.03

RD: gw = 1 2.00 0.01 2.60 0.01 3.84 0.02

RD: lw = -0.3 and gw = 1 1.99 0.01 2.61 0.01 3.85 0.02

RD: lw = 1 and gw = 1 2.05 0.01 2.58 0.01 3.73 0.03

a Lorentzian (lw) and Gaussian (gw) window functions are specified in Hz. "Only FT" refers to processing without any window function applied, while RD indicates processing with reference deconvolution.

**Table S39:** Average diffusion coefficient differences (∆*D* = *D*_alcohol_ *− D*_TSP_) and their associated propagated errors (both in *×*10*^−^*^10^ m^2^ s^-1^) for butanol, propanol, and ethanol under various processing conditions. All processing parameters were selected based on the experimental line-width at half

height of the TSP signal (0.5 Hz).

**Processing Methodsa Butanol Propanol Ethanol**

∆*D* Error ∆*D* Error ∆*D* Error

Only FT 1.93 0.30 2.56 0.34 3.89 0.57

lw = 0.5 2.00 0.12 2.59 0.15 3.82 0.19

gw = 0.5 2.01 0.13 2.60 0.17 3.86 0.22

lw = -0.5 and gw = 0.5 1.94 0.29 2.58 0.34 3.91 0.50

lw = 0.5 and gw = 0.5 2.01 0.08 2.62 0.09 3.82 0.11

RD | lw = 0.5 2.00 0.01 2.62 0.01 3.80 0.02

RD | gw = 0.5 1.99 0.01 2.62 0.01 3.82 0.02

RD | lw = -0.5 and gw = 0.5 1.97 0.01 2.63 0.02 3.82 0.03

RD | lw = 0.5 and gw = 0.5 2.01 0.01 2.61 0.01 3.79 0.02

a Lorentzian (lw) and Gaussian (gw) window functions are specified in Hz. “Only FT” refers to processing without any window function applied, while RD indicates processing with reference deconvolution.

**Table S40:** Average diffusion coefficient differences (∆*D* = *D*_alcohol_ *− D*_TSP_) and their associated propagated errors (both in *×*10*^−^*^10^ m^2^ s^-1^) for butanol, propanol, and ethanol. All processing was performed with a Gaussian window function of 1 Hz and various automatic baseline correction orders.

**Automatic Baseline Order Butanol Propanol Ethanol**

∆*D* Error ∆*D* Error ∆*D* Error

0 2.00 0.07 2.62 0.07 3.85 0.09

1 2.00 0.07 2.62 0.07 3.86 0.09

3 2.00 0.07 2.62 0.07 3.86 0.09

5 2.01 0.07 2.62 0.07 3.86 0.09

8 2.00 0.07 2.62 0.07 3.86 0.09

**Table S41:** Average diffusion coefficient differences (∆*D* = *D*_alcohol_ *− D*_TSP_) and their associated propagated errors (both in *×*10*^−^*^10^ m^2^ s^-1^) for butanol, propanol, and ethanol. All processing was performed with a Gaussian window function of 1 Hz and various manual baseline correction orders.

**Manual Baseline Order Butanol Propanol Ethanol**

∆*D* Error ∆*D* Error ∆*D* Error

0 2.00 0.07 2.62 0.07 3.85 0.09

1 2.00 0.07 2.62 0.07 3.85 0.09

3 2.00 0.07 2.62 0.07 3.85 0.09

5 2.00 0.07 2.62 0.07 3.85 0.09

8 2.01 0.07 2.63 0.07 3.87 0.09

**Figure S47: A)** Diffusion coefficients of ethanol, propanol, butanol, and TSP in their mixture in deuterated water, along with their associated errors across ten different processing methods. **B)** The

trends in the relative diffusion differences (∆*D*ethanol, propanol, butanol = *D*ethanol, propanol, butanol *− D*TSP) and

the corresponding propagated errors between ethanol and TSP, propanol and TSP, and butanol and

TSP across the different processing methods.

The average diffusion coefficients and propagated errors presented here were calculated following the procedures described in the literature.[^1^](#_bookmark39)^,^[^2^](#_bookmark40)

# Effect of Reference Deconvolution on the TSP Signal

**(A)** No Reference Deconvolution **(B)** Reference Deconvolution

Zoom

x 6.5

**Figure S48:** 1H NMR (400 MHz) signal of TSP **A)** before and **B)** after the application of Reference Deconvolution using a Lorentzian window function (LW) of 1 Hz.

1T. L. G. Cabral, G. Dal Poggetto, J. P. da Silva, M. Nilsson, C. F. Tormena, Determining the absolute configuration of small molecules by diffusion NMR experiments, *Angew. Chem. Int. Ed.*, **2025**, 137, e202418508.

2T. L. G. Cabral, J. P. da Silva, C. F. Tormena, M. Stein, Molecular Recognition and Chiral Discrimination from NMR

and Multi-Scale Simulations, *Chem. Eur. J.*, **2025**, 31, e202404694.
